# Mutational patterns and clonal evolution from diagnosis to relapse in pediatric acute lymphoblastic leukemia

**DOI:** 10.1038/s41598-021-95109-0

**Published:** 2021-08-06

**Authors:** Shumaila Sayyab, Anders Lundmark, Malin Larsson, Markus Ringnér, Sara Nystedt, Yanara Marincevic-Zuniga, Katja Pokrovskaja Tamm, Jonas Abrahamsson, Linda Fogelstrand, Mats Heyman, Ulrika Norén-Nyström, Gudmar Lönnerholm, Arja Harila-Saari, Eva C. Berglund, Jessica Nordlund, Ann-Christine Syvänen

**Affiliations:** 1 Department of Medical Sciences, Molecular Medicine and Science for Life Laboratory, Uppsala University, Box 1432, 75144 Uppsala, Sweden; 2 Department of Physics, Chemistry and Biology, National Bioinformatics Infrastructure Sweden, Science for Life Laboratory, Linköping University, Linköping, Sweden; 3 Department of Biology, National Bioinformatics Infrastructure Sweden, Science for Life Laboratory, Lund University, Lund, Sweden; 4 Department of Oncology-Pathology, Karolinska Institutet, Stockholm, Sweden; 5 Department of Pediatrics, Institute of Clinical Sciences, Sahlgrenska Academy at University of Gothenburg, Gothenburg, Sweden; 6 Department of Laboratory Medicine, Institute of Biomedicine, Sahlgrenska Academy at University of Gothenburg, Gothenburg, Sweden; 7 Department of Clinical Chemistry, Sahlgrenska University Hospital, Gothenburg, Sweden; 8 Childhood Cancer Research Unit, Karolinska University Hospital, Stockholm, Sweden; 9 Department of Clinical Sciences and Pediatrics, University of Umeå, Umeå, Sweden; 10 Department of Women’s and Children’s Health, Uppsala University, Uppsala, Sweden; 11 For the Nordic Society of Pediatric Hematology and Oncology, Stockholm, Sweden

**Keywords:** Cancer, Acute lymphocytic leukaemia, Genomics, Next-generation sequencing, RNA sequencing, Cancer genomics, Clinical genetics

## Abstract

The mechanisms driving clonal heterogeneity and evolution in relapsed pediatric acute lymphoblastic leukemia (ALL) are not fully understood. We performed whole genome sequencing of samples collected at diagnosis, relapse(s) and remission from 29 Nordic patients. Somatic point mutations and large-scale structural variants were called using individually matched remission samples as controls, and allelic expression of the mutations was assessed in ALL cells using RNA-sequencing. We observed an increased burden of somatic mutations at relapse, compared to diagnosis, and at second relapse compared to first relapse. In addition to 29 known ALL driver genes, of which nine genes carried recurrent protein-coding mutations in our sample set, we identified putative non-protein coding mutations in regulatory regions of seven additional genes that have not previously been described in ALL. Cluster analysis of hundreds of somatic mutations per sample revealed three distinct evolutionary trajectories during ALL progression from diagnosis to relapse. The evolutionary trajectories provide insight into the mutational mechanisms leading relapse in ALL and could offer biomarkers for improved risk prediction in individual patients.

## Introduction

Acute pediatric lymphoblastic leukemia (ALL) is a malignancy with marked heterogeneity in molecular and clinical phenotypes. With protocol-based aggressive combined chemotherapy regimens the 5-year survival rates in pediatric ALL are now approaching 90%^1^. Despite such remarkable improvements, approximately 10–20% of patients suffer from relapse or resistant disease or experience adverse drug effects during treatment^2^. In patients treated according to the Nordic Society for Pediatric Hematology and Oncology (NOPHO) ALL-2008 protocol the frequency of severe adverse effects was as high as 60%^3^. After relapse, the 5-year survival rate of the patients drops to ~ 40–70%, depending on treatment protocol and molecular subtype^4^. Novel biomarkers are needed to improve the prediction of which patients could benefit from intensified and novel targeted treatments. On the genetic level, pediatric ALL is grouped into subtypes characterized by large-scale chromosomal aberrations that are believed to be the disease-initiating lesions. Together, structural variations and acquisition of mutations in key genes and pathways contribute to leukemogenesis^2^.

Clonal heterogeneity is common in ALL. One of the first studies using genome-wide SNP genotyping in relapsed ALL indicated that clones present at relapse were derived from clones that were ancestral relative to the diagnostic clone^5^. By harnessing the improved resolution from whole genome sequencing (WGS), a more recent study demonstrated that the clones dominating at ALL relapse likely evolve from a minor clone present at diagnosis and that relapse-associated mutations are concomitant with chemotherapy resistance^6^. It is well known that relapse-associated mutations in ALL are specifically selected upon by chemotherapy, exemplified by mutations in *KRAS, NRAS* and *PTPN11*, which illustrates a chemotherapy driven selection mechanism for clonal evolution^7^. The mutational dynamics differ between patients with early and late relapse, with earlier relapses showing an overrepresentation of mutations in DNA repair genes^8^. Collectively, studies on relapsed ALL have uncovered potentially actionable target mutations in tyrosine kinases^9^ and the RAS pathway^10^, which could be options for future individualized treatment. Although chromosomal rearrangements that lead to fusion genes are well known in ALL, our understanding of large-scale and medium sized structural mutations in ALL is still incomplete. Most studies on somatic point mutations have focused on protein-coding single nucleotide variants (SNVs) and small insertion/deletions (indels), while the role of somatic mutation that affect regulatory regions of the genome has not yet been explored in ALL. We therefore determined the genome-wide patterns of somatic mutations in Nordic pediatric ALL patients using WGS and RNA-sequencing (RNA-seq). Our aim was to identify the somatic mutations with the largest impact on the development of relapse in ALL, and to define the clonal evolution patterns from diagnosis to relapse based on a comprehensive set of somatic mutations.

## Results

To determine the mutational patterns leading to relapse and to delineate the clonal evolution of the ALL cells due to somatic point mutations and structural aberrations, we sequenced 96 genomes from matched diagnostic, relapse and remission samples from 29 Nordic patients with pediatric ALL (Table 1). Somatic point mutations and large structural aberrations were identified in the ALL samples by comparison with the matched germline samples.

**Table 1:** Clinical information for 29 patients with acute lymphoblastic leukemia (ALL) included in the study.

Patient Id^a^	Immuno-phenotype	Genetic subtype at diagnosis	Revised subtype^b^	Risk group^c^	Time to first relapse (months)	First relapse on or off therapy^d^	Time to second relapse (months)^e^	Dead or alive^f^
ALL_5*	BCP-ALL	*KMT2A*-rearranged	*KMT2A*-rearranged	Infant	28	Off	40	D
ALL_202	BCP-ALL	*KMT2A*-rearranged	*KMT2A*-rearranged	Infant	5	On	na	D
ALL_680	BCP-ALL	dic(9;20)	dic(9;20)	HR	35	Off	na	A
ALL_205	BCP-ALL	normal	*DUX4*-rearranged^**+**^	IR	65	Off	na	A
ALL_831	BCP-ALL	HeH	HeH	HR	55	Off	na	A
ALL_832*	BCP-ALL	HeH	HeH	SR	39	Off	48	D
ALL_837	BCP-ALL	HeH	HeH	SR	40	Off	na	A
ALL_48	BCP-ALL	HeH	HeH	IR	38	Off	na	A
ALL_210	BCP-ALL	HeH	HeH	SR	48	Off	na	A
ALL_839	BCP-ALL	HeH	HeH	HR	20	On	na	A
ALL_380	BCP-ALL	HeH	HeH	IR	47	Off	na	A
ALL_464	BCP-ALL	HeH	HeH	HR	28	Off	na	D
ALL_317	BCP-ALL	iAMP21	iAMP21	IR	44	Off	na	A
ALL_838	BCP-ALL	normal	B-other group^**+**^	IR	45	Off	na	D
ALL_24	BCP-ALL	B-other group	B-other group	HR	27	Off	na	D
ALL_128	BCP-ALL	B-other group	B-other group	IR	38	Off	61	D
ALL_244	BCP-ALL	B-other group	*MEF2D*-rearranged^**+**^	HR	10	On	19	D
ALL_827	MPAL	ALL/AML	MPAL	HR	18	On	na	D
ALL_834	BCP-ALL	normal	B-other group^**+**^	SR	37	Off	64	D
ALL_27	BCP-ALL	normal	B-other group^**+**^	IR	85	Off	100	D
ALL_833	T-ALL	T-ALL	T-ALL	HR	5	On	9	D
ALL_836	T-ALL	T-ALL	T-ALL	HR	18	On	22	A
ALL_358	T-ALL	T-ALL	T-ALL	HR	18	On	na	D
ALL_388	T-ALL	T-ALL	T-ALL	HR	14	On	Na	A
ALL_835	BCP-ALL	t(12;21)	t(12;21)	SR	53	Off	Na	A
ALL_109*	BCP-ALL	t(12;21)	t(12;21)	HR	24	Off	45	D
ALL_124	BCP-ALL	t(12;21)	t(12;21)	SR	35	Off	Na	D
ALL_257	BCP-ALL	normal	*ZNF384*-rearranged^**+**^	IR	38	Off	Na	A
ALL_8	BCP-ALL	B-other group	*ZNF384*-rearranged^**+**^	IR	40	Off	Na	A

^a^Bone marrow transplantated after first relapse "*".

^b^The karyotypes of the ALL samples had been assigned according to the International System for Human Cytogenetic Nomenclature^29^. Revised subtype based on whole genome sequencing data marked with ‘+’; HeH, high hyperdiploidy (51–67 chromosomes); t(12;21),translocation between chromosomes t(12;21)(p13;q22) *ETV6-RUNX1*; iAMP21, intrachromosomal amplification of chromosome 21; dic(9;20), dicentric chromosome (9;20)(p13;q11); *ZNF384*-rearranged, translocation between *ZNF384* and fusion partner genes; *KMT2A*-rearranged, translocation between *KMT2A* and fusion partner genes; *DUX4*-rearranged, translocation between *DUX4* and fusion partner genes; *MEF2D*-rearranged, translocation between *MEF2D* and fusion partner genes; B-other group, unclassified, non-recurrent clonal aberrations; T-ALL, T-cell immuno-phenotype; MPAL, mixed phenotype acute leukemia; ALL_834 and ALL_27 are patients with Down's syndrome in the B-other group.

^c^High risk (HR), standard risk (SR), intermediate risk (IR), infant risk groups.

^d^SR patients treated 30 months, HR patients 24 months, IR patients 24 or 30 months^35^.

^e^Time to second relapse from diagnosis.

^f^Deceased (D) or Alive (A).

### Somatic single nucleotide variants and small insertion/deletions

**Figure 1:**
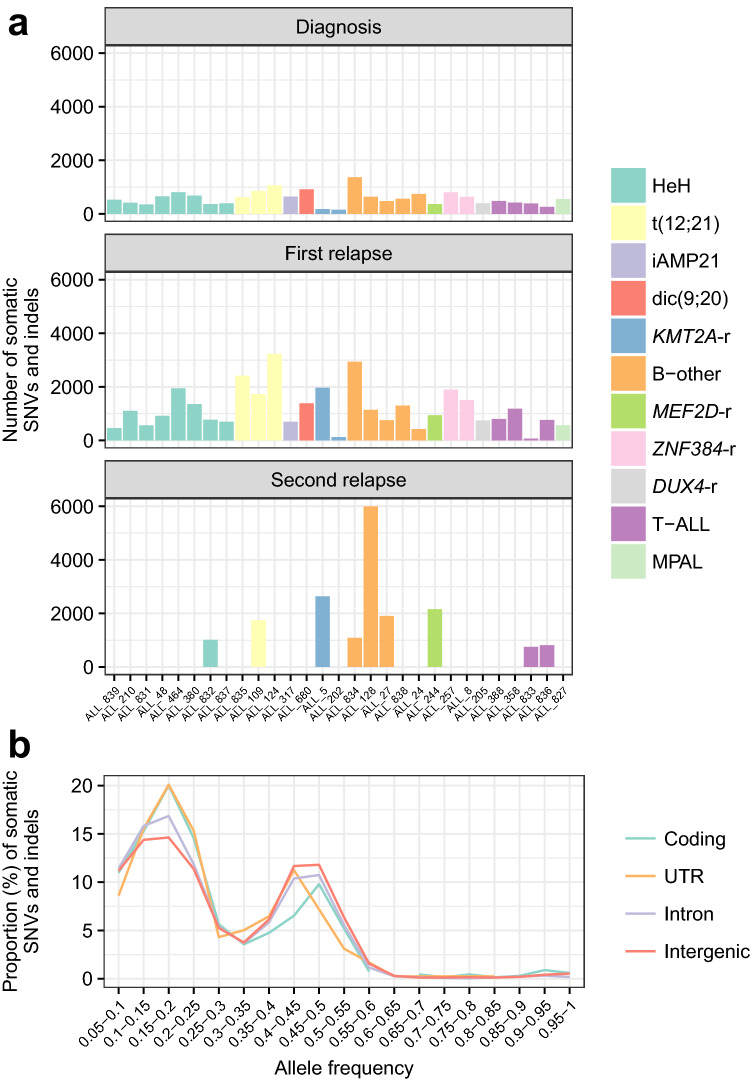
Distribution of somatic mutations in 29 patients with pediatric ALL. **(a)** Number of somatic single nucleotide variants (SNVs) and insertion/deletions in 29 ALL patients at diagnosis, first and second relapse are shown. The genetic subtypes are color-coded. **(b)** Allele frequency (AF) distribution of somatic mutations across genomic regions. Each region displays an AF peak between 0.35 and 0.50, which indicates mutations in the major clone, together with an AF peak between 0.1 and 0.25, which indicates subclonal mutations (*UTR* untranslated region).

We identified a median of 524 somatic point mutations per patient at diagnosis (range 152–1343), a median of 923 mutations at first relapse (range 68–3156), and a median of 1566 mutations (range 730–5839) at second relapse. The distribution of somatic mutations differed largely between the ALL subtypes and between individual samples within a subtype (Fig. 1a, Supplementary Tables S1, S2). The allele frequency (AF) distribution of the mutations showed major peaks at AF 0.35–0.5, corresponding to mutations present in the main leukemic clone, and at AF 0.1–0.25 reflecting subclonal mutations (Fig. 1b).

### Single base substitution mutational signatures

**Figure2:**
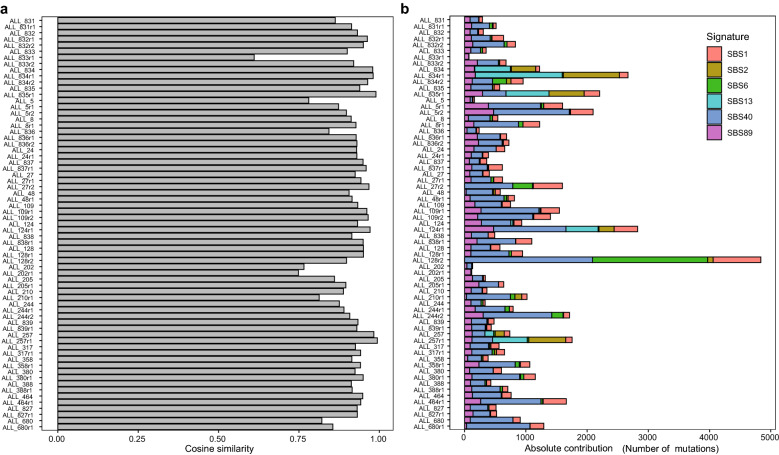
Contribution of single-base mutational signatures to individual ALL samples. **(a)** The cosine similarities between observed mutations and mutations reconstructed using the set of six COSMIC mutational signatures (SBS1, SBS2, SBS6, SBS13, SBS40 and SBS89) for each of the 67 individual ALL genomes. The four samples with the lowest cosine similarities (ALL_833r1, ALL_202r1, ALL_202, ALL_5; cosine similarity < 0.8) are the four samples with the lowest number of mutations. **(b)** The contributions of six COSMIC signatures to 67 ALL samples. ALL sample identities are shown on the vertical axis, with first and second relapse shown by r1 and r2. The number of mutations in each sample is shown on the horizontal axis, and mutations are colored according to the associated COSMIC mutational signatures in the figure insert. Six samples at diagnosis or first relapse (ALL_834, ALL_834r1, ALL_835r1, ALL_124r1, ALL_257 and ALL257r1) harbored large numbers of mutations associated with the AID/APOBEC mutational signatures (SBS2 and SBS13). Three patients (ALL_834r2, ALL_27r2 and ALL_128r2) harbored mutations associated with SBS6 indicative of defective DNA mismatch repair at second relapse.

To investigate which of the well-characterized single base substitution (SBS) signatures in the Catalogue Of Somatic Mutations In Cancer (COSMIC) database (v3.1)^11^ that match our samples, we examined the trinucleotide context of the detected somatic SNVs. To avoid over-fitting to a large number of COSMIC signatures, we first identified three de novo single base mutational signatures (Supplementary Fig. S1a,b), which were sufficient to reconstruct the mutations in the 67 ALL genomes sequenced here with a high median cosine similarity of 0.95 (Supplementary Fig. S1c). Next, we compared our de novo signatures to the COSMIC signatures (Supplementary Fig. S1d) and selected the smallest set of COSMIC signatures that reconstructs the observed mutations equally well as our de novo signatures. Through this process (see the Methods section for steps of the process), we identified the following six COSMIC signatures that are candidates for describing the underlying mutational processes in the 67 ALL genomes: SBS1, SBS2, SBS6, SBS13, SBS40 and SBS89^11–13^. This limited set of COSMIC signatures reconstructs the observed mutations in the ALL samples with a median cosine similarity = 0.93, which was not significantly different from that of the three de novo signatures (t-test, p = 0.17, Supplementary Fig. S1c). Reassuringly, most of our samples are reconstructed well by the COSMIC signatures, and the samples that are not well reconstructed contain few mutations (Fig. 2a). The signature SBS89 contributed only to a low extent to description of the original mutations, and could be replaced with another similar COSMIC signature (Supplementary Fig. S1e) with comparable performance.

Armed with a set of six COSMIC signatures, we next investigated their contribution to the mutational load in each sample (Fig. 2b). SBS1, SBS40 and SBS89 were present in essentially all samples. SBS1 is “clock-like” in that the number of mutations correlates with the age of the individual, while SBS40 and SBS89 are of unknown etiology.

SBS2 and SBS13 are attributed to activity of the AID/APOBEC family of cytidine deaminases. Four patients harbored numerous mutations of these signatures at first relapse, and two of the patients harbored them already at diagnosis (Fig. 2b). SBS6 is attributed to defective DNA mismatch repair. Three ALL patients in the B-other group harbored a large number of SBS6 mutations at second relapse.

### ALL driver genes

Twice as many potentially functional non-silent mutations in protein-coding regions were identified in ALL samples at first relapse than at diagnosis, and at second relapse the number of mutations increased ~ twofold (Supplementary Table S3). Genes carrying non-silent somatic mutations at diagnosis and/or relapse were considered as driver genes critical for the development of ALL if the mutations occurred more frequently than expected from background mutational rates^14^, or if signals of positive selection supported their involvement in cancer development^15^. These criteria underlie the MutSigCV and Oncodrive-fml softwares used in our analysis^14,15^. In addition, genes carrying non-silent mutations were defined as driver genes if they carried a recurrent clonal or subclonal, non-silent somatic SNV or indel, unless a mutation was located in a known ALL driver gene, in which case a single patient was considered sufficient^6,16,17^. In our Nordic patients this analysis of SNVs and indels at diagnosis and relapse(s) replicated 29 genes previously established to be drivers of ALL^6,16–22^. Nine genes were recurrently mutated at diagnosis and/or relapse in two to ten patients and 20 genes carried driver mutations in one sample only (Supplementary Fig. S2a). The changes from diagnosis to relapse(s) in AF for non-silent somatic SNVs and indels with AF > 0.05 in the ALL driver genes are shown in Supplementary Fig. S2b. Most of the mutations that were enriched at first relapse persisted to second relapse, with the exception of *NRAS* mutations at diagnosis, which had often been eradicated at first relapse. Moreover, a mutant SNV allele was required to be expressed in at least one of the ALL samples according to RNA-seq to call a driver gene. Supplementary Fig. S2c illustrates the allele-specific expression of the mutant alleles of the analyzed ALL samples and predicted driver genes at diagnosis and relapse(s). Twenty eight out of the 29 driver genes showed allele-specific expression of the mutant SNV or indel in at least one of the samples containing the somatic mutation. Nineteen driver genes for ALL were mutated at both diagnosis and relapse (Supplementary Fig. S2), including genes encoding the epigenetic regulator proteins HDAC2 and KDM6A, members of important signaling pathways, like NRAS, KRAS, PTPN11, NOTCH1 and FBXW7, and genes encoding the B-cell developmental factors ETV6 and IKZF1. This group also includes *CREBBP*, which confers resistance to treatment with glucocorticoids and is recurrent at relapse^23^. Eight known ALL driver genes carried mutations exclusively in the relapse samples (Supplementary Fig. S2). The *PTEN* and *IL7R* genes were mutated only at diagnosis.

### Non-coding variants in regulatory regions

**Figure 3:**
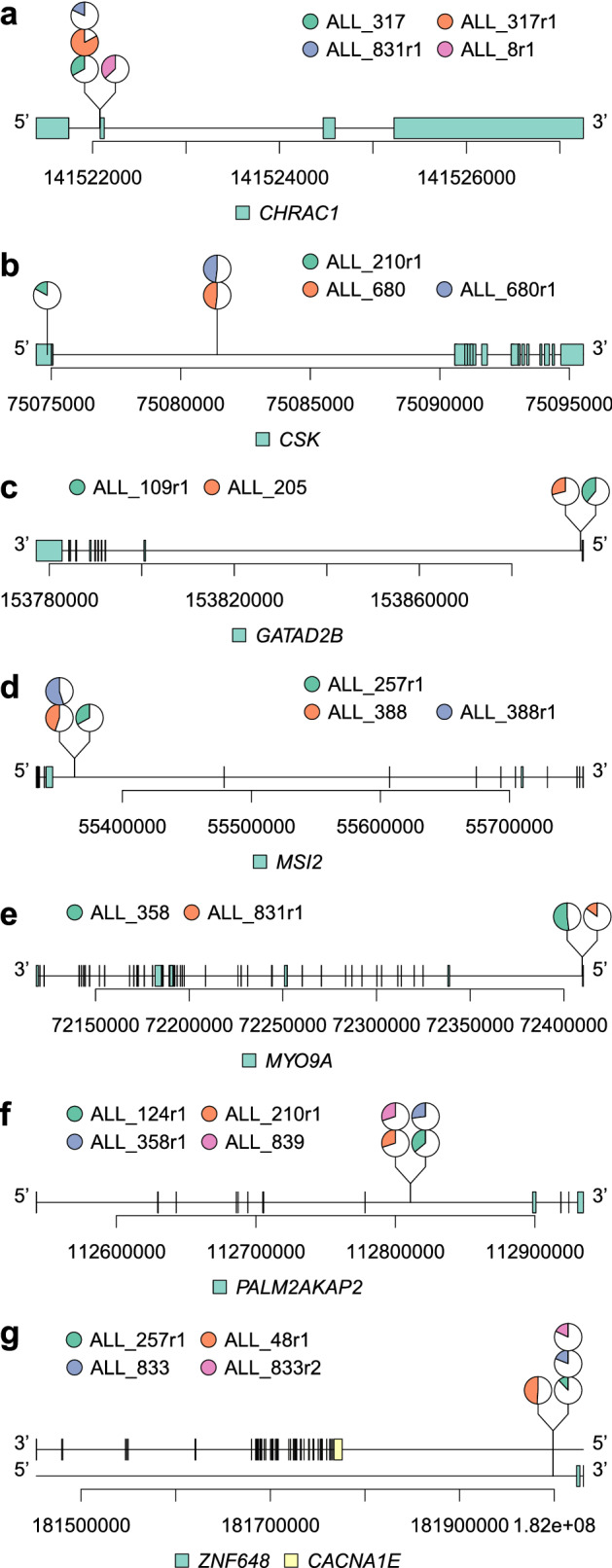
Non-coding single-nucleotide variants (SNVs) in regulatory regions of seven genes in patients with ALL. (**a**–**g**) Lollipop plots showing pie charts with the mutant allele frequencies and genomic positions of each mutation. Pie charts stacked above each other illustrate that the mutation in each sample is located at the same nucleotide position. The gene names and genome coordinates are shown at the bottom of each panel. Coding exons are indicated as vertical bars and/or turquoise boxes. The samples carrying the mutations, with first and second relapses marked with r1 and r2, respectively, are shown with the same color-code as the pie charts in the top part of each panel. With the exception of the SNVs in the *CSK* gene (**b**), the somatic SNVs in each gene are located within a small fragment of 5–19 bp.

For our study we selected somatic non-coding mutations in genomic regions marked as regulatory active by open chromatin (DNase1-HS), active promoters (H3K4me3) or enhancers (H3K4me1 and H3K27ac) or in conserved transcription factor binding sites (Supplementary Table S4) as defined in lymphoblastoid cell lines (LCLs) or in primary ALL cells^24,25^. Non-coding somatic SNVs and indels were considered to be in the same genomic region if they were present within the same 100 base pair (bp) region in at least two patients with ALL. This analysis detected potential *cis*-acting SNVs in regulatory regions of eight genes in 18 patients at diagnosis and/or relapse (Fig. 3, Supplementary Table S4). Ten of the SNVs were located in the first intron/5’UTR of *CHRAC1*, *GATAD2B, MYO9A*, *CSK* or *PALM2-AKAP2*, two SNVs were located in the fifth intron of *MSI2*, and two SNVs were located in an intergenic region 23 kb and 234 kb downstream of *ZNF648* and *CACNA1E*, respectively (Fig. 3). With the exception of the *CSK* gene*,* the putative regulatory SNVs were located within a < 20 bp hotspot region (size range 5–19 bp) in each gene, which suggests that they are part of the same regulatory element (Fig. 3). Four of the non-coding SNVs were mutated in two patients at exactly the same genomic position. These SNVs were in the *CHRAC1, PALM2-AKAP2* and *ZNF648/CACNAE1* genes (Fig. 3), which further supports a functional role for them in ALL.

Since driver genes often are disrupted by different mechanisms in different patients, coding mutations identified in other patients add support to the functional effect of mutations in *cis*-acting regulatory elements of a gene. The relevance of *CACNA1E* was in our cohort supported by a missense mutation in one patient in addition to the three patients with non-coding mutations. Moreover, more than 2% of ~ 5000 samples from patients with haematopoietic and lymphoid cancers included in the COSMIC Release v94 database carried coding mutations in *MYO9A, PALM2-AKAP2, MSI2 or CACNA1E* (range 121–202 mutated samples per gene) (Supplementary Table S4).

Each of the seven genes with non-coding somatic mutations were expressed in the patient carrying the putative regulatory non-coding variant with log2 of FPKM (fragments per kilobase per million mapped reads) value > 1 (Supplementary Table S4). We used the *cis*- eXpression (cis-X) tool^26^ to further evaluate the regulatory role of the non-coding variants in the affected samples, and report allele specific expression (ASE) and outlier high expression (OHE). ASE and OHE did not provide additional support for a *cis*-acting role for non-coding variants in our samples (Supplementary Table S4).

### Large-scale genetic alterations in ALL

Since large-scale structural aberrations are a major hallmark of ALL, we utilized the WGS data to reconstruct precise digital karyotypes for the 67 ALL genomes (Supplementary Table S5). The digital karyotypes allowed validation of the cytogenetic karyotype in all 29 diagnostic samples and identification of previously undetected somatic CNAs in 22 of the 29, diagnostic samples. The size of the novel CNAs varied from a few exons to full-length chromosomes. Frequently occurring deletions of chromosome 9p21.3 and the majority of CNAs on the other chromosomes persisted from diagnosis to relapse, with the exception of CNAs in three patients (ALL_48, ALL_124 and ALL_831) whose ALL genomes had altered ploidy states at relapse. In seven of the patients, we observed CNAs that were not present at diagnosis, but occurred de novo at relapses. The digital karyotypes determined by WGS allowed identification of the exact genomic breakpoints of expressed fusion genes, and the exons involved in the gene fusions were verified in the RNA-seq data (Supplementary Table S6). Herein, we describe a novel in-frame balanced translocation t(7;12)(p15;p13) that results in expression of the *ETV6-TSL* fusion gene at diagnosis and relapse in patient ALL_827 with mixed phenotype acute leukemia (MPAL). Split reads between the *ETV6* (exon 2) and *TSL* (exon 2) in RNA-seq data and identification of the genomic breakpoints giving rise to this fusion gene in the WGS data at chr7:27,536,526 (120 kb upstream of *TSL*) and in intron 2 of *ETV6* on chr12:11,905,675 confirm the presence of the *ETV6-TSL* fusion gene at diagnosis and relapse in this patient (Supplementary Fig. S3).

**Figure 4:**
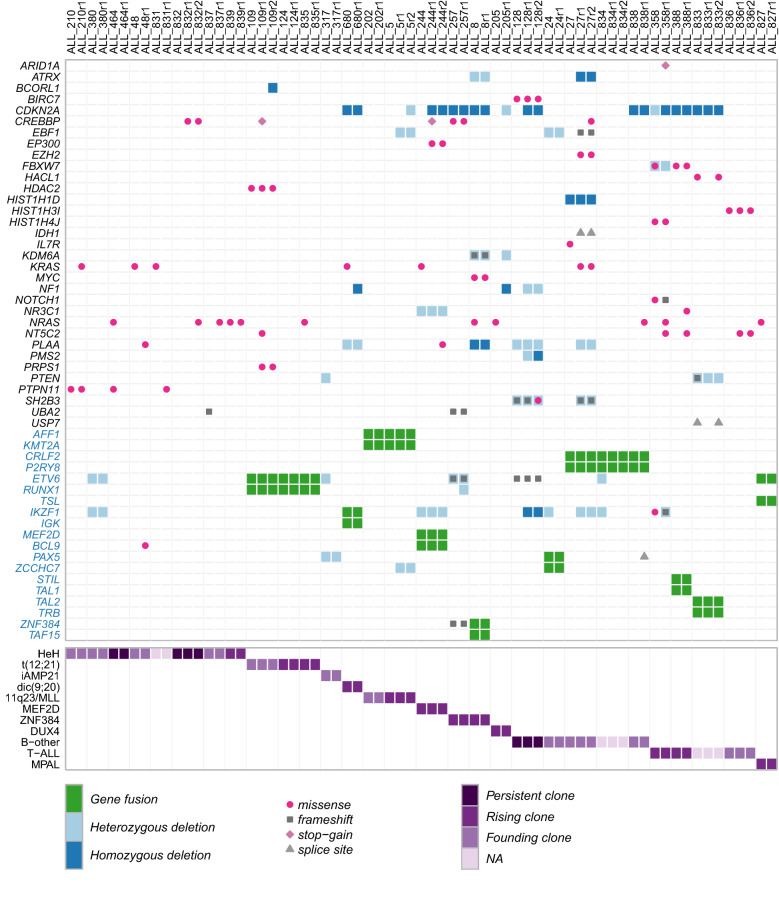
Distribution of somatic driver mutations, copy number losses and gene fusions at diagnosis and relapse. Genes are included in the figure if they were defined as driver genes in the present study based on somatic SNVs and indels (gene acronyms in black), or if they are listed in the OncoKB database and contain copy number loss in at least one analysed sample (gene acronyms black), or if they are part of gene fusions that define ALL subtypes (fusion partner gene acronyms in blue). Columns represent samples at diagnosis and relapse(s) from the 29 ALL patients. Sample names are shown above the top panel, and genetic subtypes as well as the assigned trajectories of clonal evolution are indicated in the bottom panel. The type of genomic alteration detected in each sample is color coded as indicate at the bottom of the panel.

In total, 11 patients had a homozygous deletion of at least one of seven tumor suppressor genes (Fig. 4). Genes affected by deletions of one allele combined with a non-silent protein-coding mutation on the other allele are critical candidate genes for affecting ALL. This type of biallelic loss of function was observed in *ETV6, IKZF1, KDM6A*, *PTEN, SH2B3,* and *FBXW7* in six patients (Fig. 4).

### Clonal evolution trajectories

The changes in AF of somatic SNVs were examined to determine the clonal and subclonal evolutionary patterns of the 29 ALL genomes from diagnosis to relapse. We assigned a monoclonal evolutionary consensus model for 26 of the ALL patients, with a probability score above 0.8 for 20 of the patients (Table 2, Supplementary Fig. S4). The remaining three patients (ALL_831, ALL_833 and ALL_834) were not analyzed for clonal evolution trajectories due to either low estimated tumor content in the relapse samples or an independent tumor development. Of the 26 patients, for whom an evolutionary trajectory was assigned, four patients were assigned evolutionary trajectories with low probability scores (< 0.65). The subtypes of these patients were HeH (n = 3) and dic(9;20) (n = 1). The probability scores for the individual patients are provided in Table 2.

**Table 2:** Characteristics of clonal evolutionary trajectories for patients with acute lymphoblastic leukemia (ALL).

Trajectory	Subtype or subgroup^a^	Patient ID	Probablity score^c^	Fusion gene	Critical genes with recurrent mutations^d^		
					Diagnosis	First relapse	Second relapse
Persistent clone	B-other	ALL_128	1	na	*BIRC7, SH2B3, ETV6, PLAA*	*CDKN2A, BIRC7, SH2B3, ETV6, PLAA, PMS2, NF1, IKZF1*	*CDKN2A, BIRC7, SH2B3, ETV6, PLAA, PMS2, NF1, IKZF1*
	HeH	ALL_464	0.1	na	*PTPN11,NRAS*	*na*	*na*
	HeH	ALL_832	0.8	na	*na*	*CREBBP*	*CREBBP, NRAS*
Rising clone	t(12;21)	ALL_124	1	*ETV6-RUNX1*	*na*	*na*	*na*
	*DUX4-rearranged*	ALL_205	1	*DUX4-IGH*	*NRAS, GATAD2B*	*CDKN2A, KDM6A, NF1*	*na*
	*MEF2D-rearranged*	ALL_244	1	*MEF2D-BCL9*	*KRAS, IKZF1, NR3C1*	*CDKN2A, CREBBP, EP300, IKZF1, NR3C1*	*CDKN2A, EP300, IKZF1, NR3C1, PLAA*
	*ZNF384-rearranged*	ALL_257	1	na	*CDKN2A, ETV6,CREBBP, ZNF384, UBA2, MSI2*	*CDKN2A, ETV6,CREBBP, ZNF384, UBA2, MSI2*	*na*
	T-ALL	ALL_358	1	na	*IKZF1, NOTCH1, HIST1H4J, FBXW7, MYO9A*	*CDKN2A, IKZF1, NT5C2, NRAS, ARID1A, NOTCH1, HIST1H4J, PALM2*	*na*
	T-ALL	ALL_388	1	*STIL-TAL1*	*CDKN2A, FBXW7, MSI2*	*CDKN2A, FBXW7, NT5C2, NR3C1, MSI2*	*na*
	*KMT2A-rearranged*	ALL_5	0.99	*AFF1-KMT2A*	*na*	*EBF1, ZCCHC7*	*CDKN2A, EBF1, ZCCHC7*
	dic(9;20)	ALL_680	0.4	*IKZF1-IGK*	*CDKN2A, KRAS, PLAA, CSK*	*CDKN2A,PLAA, NF1, CSK*	*na*
	*ZNF384-rearranged*	ALL_8	1	*ZNF384-TAF15*	*CDKN2A, KDM6A, NRAS, ATRX, PLAA, CHRAC1, MYC*	*CDKN2A, KDM6A, ATRX, PLAA, CHRAC1, MYC*	*na*
	MPAL	ALL_827	1	*ETV6-TSL*	*NRAS*	*na*	*na*
	t(12;21)	ALL_835	1	*ETV6-RUNX1*	*NRAS*	*na*	*na*
	HeH	ALL_839	0.6	na	*NRAS, PALM2*	*NRAS*	*na*
Founding clone	t(12;21)	ALL_109	1	*ETV6-RUNX1*	*HDAC2*	*HDAC2, CREBBP, NT5C2, GATAD2B, PRPS1*	*HDAC2, BCORL1,PRPS1*
	*KMT2A-rearranged*	ALL_202	0.7	*AFF1-KMT2A*	*na*	*na*	*na*
	HeH	ALL_210	0.07	na	*PTPN11,CSK*	*PTPN11,KRAS, CSK, PALM2*	*na*
	B-other group	ALL_24	1	*PAX5-ZCCHC7*	*IKZF1, EBF1*	*EBF1*	*na*
	B-other	ALL_27	0.98	*CRLF2-P2RY8*	*HIST1H1D, IL7R*	*KRAS, SH2B3, EZH2, IDH1, EBF1, PLAA, IKZF1, ATRX, HIST1H1D*	*KRAS, SH2B3, CREBBP, EZH2, IDH1, EBF1, PLAA, IKZF1, ATRX, HIST1H1D*
	iAMP21	ALL_317	1	na	*ETV6, PTEN, PAX5, CHRAC1*	*PAX5, CHRAC1*	*na*
	HeH	ALL_380	1	na	*IKZF1, ETV6*	*IKZF1, ETV6*	*na*
	HeH	ALL_48	1	na	*KRAS*	*PLAA*	na
	T-ALL	ALL_836	1	na	*HIST1H3I*	*HIST1H3I, NT5C2*	*HIST1H3I, NT5C2*
	HeH	ALL_837	1	na	*UBA2*	*NRAS*	na
	B-other	ALL_838	1	*CRLF2-P2RY8*	*CDKN2A*	*CDKN2A, PAX5, NRAS*	na
Undetermined^b^	B-other group	ALL_834	na	*CRLF2-P2RY8*	*IKZF1, ETV6*	*na*	*na*
	HeH	ALL_831	na	na	*KRAS*	*PTPN11, MYO9A, CHRAC1*	*Na*
	T-ALL	ALL_833	na	*TAL2-TRB*	*CDKN2A, PTEN, PALM2, USP7*	*CDKN2A, PALM2, PTEN*	*CDKN2A, PTEN,PALM2, USP7*

^a^Genetic subtype revised based on whole genome sequencing (WGS) data; see Table 1 for more information,

^b^Undetermined evolutionary trajectory due to either low tumor content in relapse sample (ALL_833) or polyclonal origin (ALL_831, ALL_834).

^c^Probability score assigned to consensus model using ClonEvol software; score < 0.65 due to high rate of subclonal mutations or copy number imbalances.

^d^Genes with recurrent mutations (Supplementary Tables S3,S4); *na* not applicable.

We identified three distinct trajectories of clonal evolution in our sample set. They are denoted as “persistent”, “rising” and “founding” clone trajectories.

**Figure 5:**
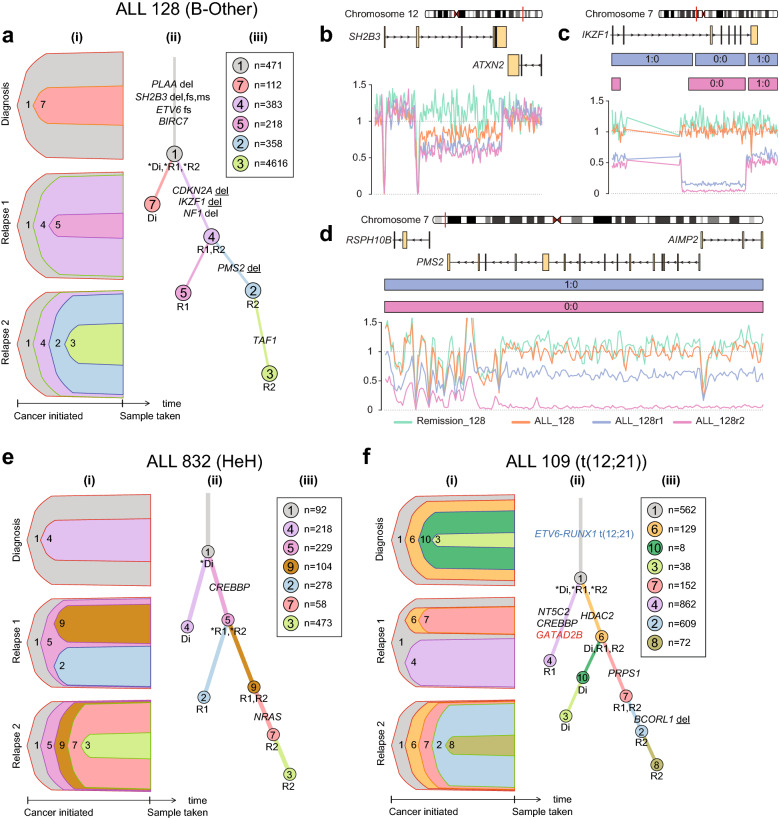
Clonal evolution in ALL patients displaying the “persistent clone” or “founding clone” trajectories. Panels a, e and f illustrate the two clonal evolutionary trajectories in three representative ALL patients. In each of the panels a, e, f section **(i)** shows the clones and subclones identified at diagnosis, first and second relapse, where clone 1 in (grey) represents the founding clone. The height of the colored fields corresponds to the proportion of the clones in a sample. Section **(ii)** shows the clones and subclones identified at diagnosis (Di), first (R1) and second relapse (R2). Each color-coded branch shows the known and putative driver genes identified at each time point, with the gene names highlighted in blue for fusion genes and in red for putative regulatory non-coding variants. Section **(iii)** shows the total number of somatic SNVs present in each clone using the same color code as in sections (i) and (ii). The most important putative driver mutations, somatic alterations: including small insertion-deletions, putatively regulatory non-coding mutations, copy number alterations and structural variants detected in our study are shown on the relevant branches of the evolutionary trees. (*del* homozygous deletion; *del* heterozygous deletion; *fs* frameshift; *sg* stop gain; *ms* missense). **(a)** Consensus model for clonal evolution in patient ALL_128 in the B-other group illustrating the “persistent clone trajectory”. **(b)** Sequencing coverage plot for the *SH2B3* gene on chromosome 12 in patient ALL_128 at diagnosis, first and second relapse and at remission (germline). **(c)** Sequencing coverage plot for the *IKZF1* gene on chromosome 7 in patient ALL_128 at diagnosis, first and second relapse. **(d)** Sequencing coverage plot for the *PMS2* gene in patient ALL_128 at diagnosis, first and second relapse. **(e)** Consensus model for clonal evolution in patient ALL_832 with high hyperdiploid (HeH) BCP-ALL illustrating the “persistent clone trajectory”. **(f)** BCP-ALL patient ALL_109 with the t(12;21) *ETV6-RUNX1* translocation illustrates the “founding clone trajectory”. Additional details on the evolutionary trajectories are provided in the Supplementary File.

**Figure 6:**
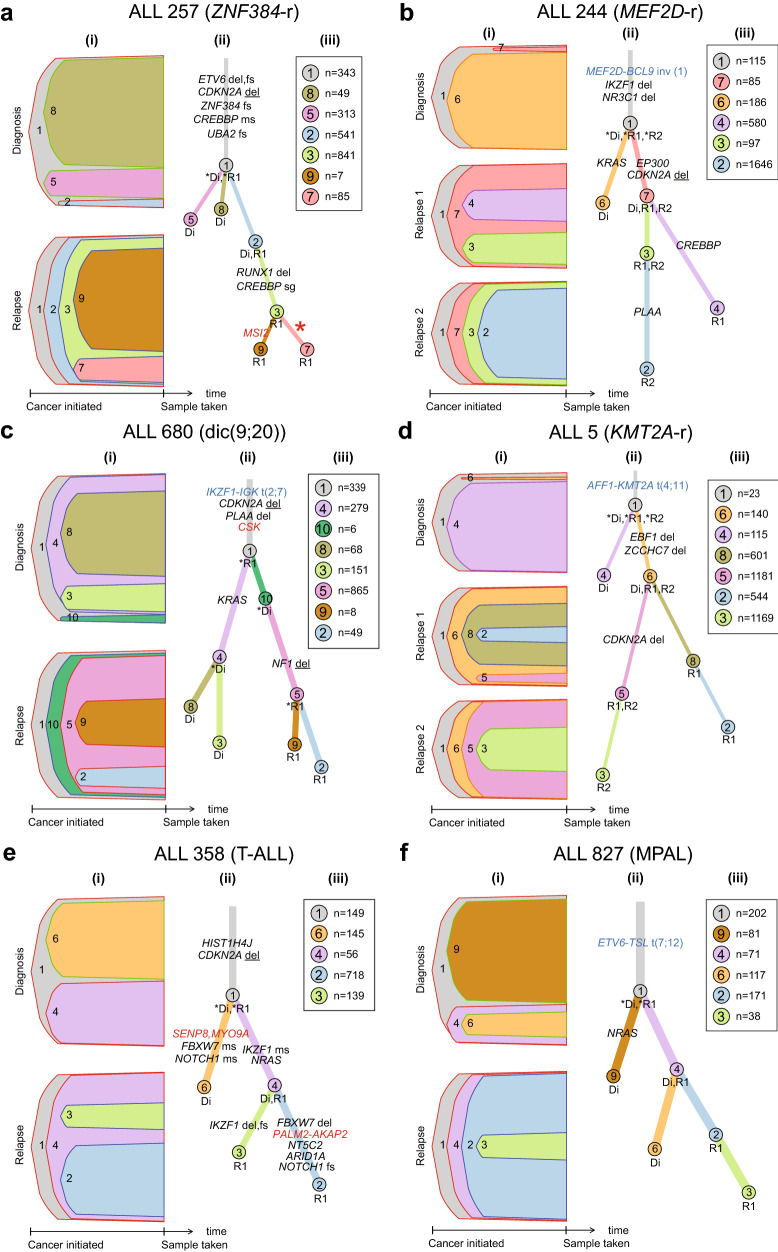
Clonal evolution from diagnosis to relapse for six representative ALL patients with the “rising clone trajectory”. **(a)** Consensus model for clonal evolution in BCP-ALL patient ALL_257 with *ZNF384*-rearranged **(b)** BCP_ALL patient ALL_244 with a *MED2F*-rearranged*,* **(c)** patient ALL_680 with the dic(9;20) subtype of BCP-ALL, **(d)** BCP-ALL patient ALL_5 with the *KMT2A*-rearranged*,* **(e)** T-ALL patient ALL_358, and **(f)** patient ALL_827 with mixed phenotype acute leukemia (MPAL). The most important putative driver mutations, somatic alterations, including small insertion-deletions, putatively regulatory non-coding mutations, copy number alterations and structural variants are shown on the relevant branches of the evolutionary trees. Details and explanations of sections denote (i–iii) are provided in the legend of Fig. 5. Additional details on the evolutionary trajectories are provided in the Supplementary File. (*del* homozygous deletion; *del* heterozygous deletion; *fs* frameshift; *sg* stop gain; *ms* missense).

In the “persistent clone trajectory”, the main clone at diagnosis persisted and had acquired additional mutations at relapse (Fig. 5a,e). In the “rising clone trajectory”, a subclone present at diagnosis expanded and acquired additional somatic mutations prior to emerging as the major clone at relapse (Fig. 6). In monoclonal evolutionary consensus models, founding clones harbor the mutations shared by all clones identified at diagnosis and relapse in each patient. In the “founding clone trajectory”, the clones at relapse were derived from the founding clone or from other clones that are ancestral relative to major or minor clones present at diagnosis (Fig. 5f). However, if the founding clone was the major clone at diagnosis, patients were assigned to the persistent clone trajectory (Fig. 5a,e).

Three of the patients were assigned to the “persistent clone trajectory”. Two patients were of the HeH subtype and one belonged to the B-other group. No expressed fusion genes were detected in these patients. Patient ALL_128 was of particular interest due to compound heterozygous aberrations in the *SH2B3* gene, where one allele was deleted and the other allele harbored a non-silent SNV that was present in all ALL cells at diagnosis, first and second relapse (Fig. 5a). Moreover, a homozygous deletion of *IKZF1* was observed at both relapses and a homozygous deletion of *PMS2*, which encodes a key component of the mismatch repair system, was observed at second relapse (Fig. 5b–d). This resulted in defective DNA mismatch repair (mutational signature SBS6) and hypermutation with ~ 6000 mutations. (Supplementary Fig. S1).

Eleven of the patients were assigned to the founding clone trajectory (Table 2, Fig. 5f).

In this trajectory the number of non-synonymous point mutations in driver genes increased from diagnosis to relapse; a total of 6 mutations at diagnosis and 18 mutations at first relapse were detected in these 11 patients (Fig. 4). This appears different from the rising clone trajectory (Fisher’s exact test, p = 0.06), but as the difference is not statistically significant it need to be evaluated in larger cohorts.

Twelve patients were assigned to the “rising clone trajectory”. A prominent feature of this trajectory is the high proportion of patients (9/12) who harbor large-scale structural aberrations resulting in expressed fusion genes (Table 2). Moreover, in this trajectory the number of non-synonymous point mutations in driver genes did not increase from diagnosis to relapse. A total of 18 driver mutations at diagnosis and 18 mutations at first relapse were detected in these 12 patients (Fig. 4). Deletions of the *CDKN2A* gene at diagnosis and/or relapse(s) is another characteristic of this trajectory observed in eight patients (8/12), compared to one patient (1/11) in the founding clone trajectory and one patient (1/3) in the persistent clone trajectory. Three of the patients in the rising clone trajectory carried heterozygous deletions of one allele together with a non-silent mutation in the other allele in the *KDM6A, IKZF1* and *ETV6* genes.

BCP-ALL patients in the high risk (HR/infant) treatment group and each of the T-ALL patients which typically suffer from severe ALL, relapsed within three years from diagnosis (Supplementary Fig. S5). However, the difference in time to relapse between the three trajectories was not statistically significant.

#### Verification of clonal evolution trajectory assignments

A potential risk with using 90 × WGS data to assign clonal trajectories is failure to detect rare subclones, which could lead to incorrect assignment of samples in the rising clone trajectory to the founding clone trajectory. To validate the trajectory assignments, we used previously generated targeted sequencing data at an average 600 × coverage (Haloplex), which was available for 16 of our 29 diagnostic samples^19^. In this data we examined if mutations that were uniquely identified in relapse samples when sequenced to 90 × were detectable in the corresponding diagnostic samples when the coverage was 600x. For 15 of the patients with available Haloplex data, the targeted sequencing did not reveal any presence of mutations in diagnostic samples that were only detected in relapse samples at 90 × coverage. Hence, targeted sequencing yielded trajectory assignments that were consistent with those at 90 × coverage for these patients. However, one mutation in the *BRINP3* gene, which was detected at relapse only in ALL_205 when sequenced to 90 × coverage, was present with an allele frequency of 0.02 in the diagnostic sample when sequenced to 600 × coverage. Based on this finding we reassigned patient ALL_205 from the founding clone to the rising clone trajectory.

#### Clonal evolution of trajectories from first to second relapse

In each of the seven patients who experienced a second relapse (Table 1) and for whom an evolutionary trajectory was assigned (Table 2), we observed either the persistent clone or rising clone trajectory from first relapse to second relapse. Two patients following the persistent clone trajectory from diagnostic to first relapse, also followed the persistent clone trajectory from the first to second relapse. This observation supports the notion that ALL which is resistant to treatment at diagnosis, will persist as resistant from first to second relapse (Supplementary Fig. S4). Four out of five patients, who followed the rising or founding clone trajectory from diagnosis to first relapse, also followed the rising or founding clone trajectory from first to second relapse. This observation is consistent with an emerging minor clone at first relapse that gives rise to the second relapse as a common feature in ALL.

## Discussion

Here we have described three categories of evolutionary trajectories and the most critical genes leading to relapse in 29 Nordic pediatric ALL patients. In line with previous studies^2,5,7,18,22^, we observe a rather silent mutational landscape at diagnosis in most patients, and that the mutational burden almost doubled successively at first and second relapse. In addition, we observed increasing genomic instability from diagnosis to first relapse in terms of an increased burden of CNAs and SVs, however the patients with the largest number of SNVs did not also have the largest mutational burden of CNAs.

We did not identify any previously unknown ALL driver genes carrying protein-coding somatic mutations, perhaps due to the small size of our sample set and the recent rapid progress in identification of driver genes in pediatric ALL. We observed the same relapse-specific driver genes (*CREBBP, NT5C2* and *PRPS1*) in our study as previous studies^23,27,28^. We and others have shown that mutations in these genes are typically acquired at relapse, but if they occur at diagnosis they remain at subsequent relapses. Reassuringly, with our limited sample set there were only small differences in the frequency of the identified relapse- and diagnosis-specific driver genes compared to other studies^6,18,22^. However, digital karyotypes constructed based on the WGS data allowed us to uncover previously unknown somatic CNAs in the majority of the ALL samples at diagnosis and relapse. We also detected homozygously deleted tumor suppressor genes and tumor suppressor genes with biallelic loss of gene function and a novel expressed fusion gene *ETV6-TSL* in a sample with mixed lineage leukemia.

Moreover, we used our genome-wide data in an exploratory search for recurrently mutated non-coding regions that hence could have cis-acting regulatory effect in ALL. Recurrent mutations across multiple patients indicate that they may have undergone positive selection during tumorigenesis. Therefore, we mapped mutational hotspots in the non-coding genomic regions and identified seven non-coding mutational hotspots linked to new genes of potential relevance for ALL. We detected 12 bp mutational hotspots in the first intron of the *MSI2* and *GATAD2B* genes, which may be particularly interesting since they contain highly conserved transcription factor or RNA polymerase binding sites in B-cells^24^. The MSI2 protein is a transcriptional regulator that targets genes involved in development and cell cycle regulation. MSI2 up-regulates expression of *FLT3* in acute myeloid leukemia via the *RUNX3* transcription factor binding site^29^. The *GATAD2B* gene encodes a subunit of the nucleosome remodeling complex and has an important role in lymphoid cell function and development^30^. To explore the impact on gene expression of the non-coding variants, we calculated ASE and OHE for our candidate genes, but did not observe significant ASE or OHE for the candidate genes carrying non-coding somatic variants. However, our data set is not optimal for investigating OHE since it is a mixture of heterogeneous ALL subtypes, and the ASE results were limited in our samples by low abundance of heterozygous SNV markers in the candidate genes. The genes presented here with non-coding variants were frequently affected by somatic coding variants in other hematopoietic and lymphoid cancers, supporting that they may be important for ALL, and that it could be interesting to study the regulatory role of these non-coding variants further.

By analyzing the somatic SNVs in a trinucleotide context we found that the mutations in our samples were accurately reconstructed by six of the well-characterized COSMIC mutational signatures^11^. The patient with the highest mutational load, ALL_128r2 with more than 6000 mutations, displayed the defective DNA mismatch repair signature (SBS6) at second relapse, most likely due to the homozygous deletion of the *PMS2* mismatch repair gene observed in this sample. A high mutational load at relapse caused by homozygous inactivation of mismatch repair genes has also been observed in previous studies of ALL^6,18,22^. Mismatch repair-deficient cancers with their large number of mutations have been suggested to be recognized by the immune system^31^. Thus, ALL patients displaying this mutational signature may be candidates to be susceptible to immunotherapy. Four patients displayed APOBEC signature mutations (SBS2/SBS13) in line with previous findings for ALL^32^.

Besides providing insight into the mutational spectrum of ALL genomes, we examined the clonal architecture of ALL and how cell populations evolve from diagnosis to relapse. Firstly, the persistent clone trajectory seems to be rare with only three patients in our sample set, suggesting that most of the pediatric ALL patients respond at least partly to the antileukemic treatments provided at diagnosis. The most common trajectory in our sample set was the rising clone trajectory followed by 12 patients. This type of trajectory is of potential clinical importance since these patients carry subclonal mutations that may confer a selective advantage or resistance to antileukemic therapy already at diagnosis and could therefore be useful for treatment stratification. The second most common trajectory in our sample set was the founding clone trajectory followed by 11 patients. ALL genomes in this trajectory experienced a three-fold increase in driver mutations from diagnosis to relapse. This contrasts the observation in the rising clone trajectory, where the number of driver mutations did not increase from diagnosis to relapse in our sample set.

Collectively these observations add further support for the hypothesis that rising clones are pre-resistant to antileukemic therapy, while the founding clones acquire additional mutations to emerge as a major clone at relapse. In both models relapse originates from clones or cells that resist therapy, but the contribution of specific driver mutations to relapse remains to be demonstrated. The utility of driver mutations for diagnosis and treatment stratification depends on the abundance of specific driver mutations at diagnosis in non-relapsed samples. Further studies in larger sample sets are required to investigate the relationship between rising and founding clone trajectories in relation to the contribution of driver genes to relapse and as potential drug targets. The potential of driver genes as drug targets is demonstrated by the fact that already at diagnosis the majority of the patients in this study displayed mutations in driver genes that encode proteins in pathways which are clinically actionable by approved drugs^33^ or are being investigated as candidate drug targets^34^. A personalized treatment strategy could be particularly beneficial for ALL patients, for whom it is today difficult to predict which patients will respond to repeated and intensified treatment.

## Patients and methods

### Patient samples

The ALL patients included in the study were diagnosed during 1993–2008 and treated at Swedish Pediatric Oncology centers (Uppsala, Umeå, Stockholm and Gothenburg) according to NOPHO ALL protocols^35^. Bone marrow or blood samples collected at diagnosis, remission and relapse from 29 patients with ALL were analyzed, including two sequential relapse samples from nine patients. Leukemic cells were isolated from the samples by density-gradient centrifugation and the proportion of leukemic cells was estimated on May-Grünwald-Giemsa-stained cytocentrifugate preparations, using light microscopy. The estimated blast count in the bone marrow and/or blood was ~ 85–95% at diagnosis and ~ 70–95% at relapse. Cell pellets were frozen and stored at − 70 °C in established institutional tissue banks until extraction of DNA and RNA. DNA and RNA was extracted from the cell pellets using the AllPrep DNA/RNA Mini Kit (Qiagen), including a DNase digestion step using RNase-Free DNase. DNA and RNA samples were stored at − 80° until analysis. Prior to sequencing, the DNA and RNA quality and concentration were measured using the Qubit dsDNA or RNA Broad‐Range assay (Invitrogen). The integrity of the RNA was assessed by capillary electrophoreses on a Bioanalyzer.

The ALL patients included in the study represent eight BCP-ALL subtypes, the B-other subgroup and T-ALL. Table 1 provides clinical information on the patients. The parents and/or the patients provided written informed consent to the study. The study was approved by the Regional Ethical Board in Uppsala, Sweden and conducted according to the Helsinki Declaration.

### Whole genome sequencing (WGS)

Sequencing libraries were prepared with 100 ng of input DNA from each patient sample using the TruSeq Nano protocol (Illumina Inc). The WGS libraries from diagnosis and relapse (except the sample ALL_244r2) were prepared in three technical replicates followed by sequencing on a HiSeqX instrument (Illumina Inc.) with paired-end 150 base pair (bp) reads and ~ 90 × coverage. Of the variant allele calls in each sample, 94.8% (n = 61,558) were concordant between three libraries and an additional 4.5% of the allele calls (n = 2941) were concordant in two libraries, evidencing for the high quality of the library preparation and the sequencing process. This allele count distribution per samples is shown in Supplementary Fig. S6. The favorable distribution of allale counts indicates robust varaiant calling and that the false positive rate is < 0.07%.

A single library was prepared from the germline (remission) samples, which were sequenced to ~ 30 × coverage. Data were processed using the Sarek workflow v1.1^36^. Sequence reads were aligned to the human reference genome hg19 using Burrows Wheeler Algorithm (BWA)^37^ and duplicate reads were marked using Picard MarkDuplicates^38^. Local realignment of regions flanking indels and recalibration of base quality scores were performed using the Genome Analysis ToolKit (GATK) version 3.7^39^.

### Detection of somatic point mutations

Somatic single nucleotide variants (SNVs) were called in the WGS data by Mutect1^40^. SNVs and small insertions and deletions (indels) were also called by Strelka version 1.0.15^41^ by running both programs in the tumor-germline mode. Additionally, Manta version 1.0.3^42^ was used to call small indels to curate the small indels from Strelka. The union of SNV calls by Mutect1 and Strelka was used for the downstream filtering and analysis. A downstream filter was used to exclude somatic mutations if (1) the sequencing depth was threefold higher than the chromosomal mean depth in each germline sample; or (2) the AF was below 5%, except in three patients at second relapse, where a bone marrow transplantation was performed prior to second relapse, or in samples where more than 90% of the SNVs overlapped SNVs in dbSNP by an AF below 10% (Table 1); or (3) the SNVs overlapped repeated regions in the University of California Santa Cruz (UCSC) browser; or (4) the SNVs overlapped or flanked homopolymers of seven base pairs or longer; or (5) the SNVs were within five base pairs from an indel called by Strelka; or (6) the SNVs were present in the SweGen^43^ or the 1000 Genomes databases^44^ containing normal genetic variation in the Swedish and European populations. Somatic mutations that passed the above criteria were annotated using the Ensembl Variant Effect Predictor (VEP)^45^. A list of somatic variants detected by WGS is provided as a supplementary data file in the aggregated variant caller (VCF) format (Supplementary data file).

### Detection of mutational signatures

Mutational signature analysis was performed using the MutationalPatterns package in R. De novo mutational signatures were identified in the ALL samples using non-negative matrix factorization (NMF)^13^. The optimal number of mutational signatures was selected based on the residual sum of squares criterion^13^. We compared our de novo signatures to the 72 single base substitution (SBS) mutational signatures in the COSMIC database (v3.1)^11^ using cosine similarity as a distance measure. With a limited number of samples de novo signatures typically resemble a mixture of COSMIC signatures, and therefore a set of COSMIC signatures similar to our signatures based on a relatively relaxed threshold of cosine similarity > 0.65 was used. This criterion resulted in a set of 12 COSMIC signatures (Supplementary Fig. S1d)^11–13^ that were sufficient to reconstruct the mutations in the 67 ALL genomes with a median cosine similarity of 0.94 (Supplementary Fig. S1c). Next we reduced the number of 12 COSMIC signatures to the smallest set that could reconstruct the mutations in the 67 ALL genomes equally well as the three de novo signatures. The 12 COSMIC signatures are similar to each other (Supplementary Fig. S1e). Three COSMIC signatures that were most similar to each of our de novo signatures were initially selected (Supplementary Fig. S1d). These three signatures (SBS6, SBS13, SBS40) reconstructed the observed mutations with a median cosine similarity = 0.83, which was significantly lower compared the three de novo signatures (*t*-test, p = 1e−15, Supplementary Fig. S1c). Next the set of COSMIC signatures was expanded by the signatures within each branch at dendrogram that was most similar to our de novo signatures (Supplementary Fig. S1d,e). This added SBS1 and SBS2 to our set. These five signatures (SBS1, SBS2, SBS6, SBS13, SBS40) reconstructed the observed mutations with median cosine similarity = 0.92, which was lower compared to the three de novo signatures (t-test, p = 0.04, Supplementary Fig. S1c). Finally, we added SBS89 to our set. SBS89 was relatively different to the other included signatures (Supplementary Fig. S1e) and this resulted in a set with two COSMIC signatures for each de novo signature (Supplementary Fig. S1d). This final set (SBS1, SBS2, SBS6, SBS13, SBS40 and SBS89) reconstructed the observed mutations with median cosine similarity = 0.93, which was not significantly different compared to using the three de novo signatures (t-test, p = 0.17, Supplementary Fig. S1c). This process to avoid overfitting our data, resulted in a limited set of six COSMIC signatures that are candidates for explaining the underlying mutational processes in the 67 ALL genomes.

### Detection of driver genes

Putative driver genes carrying non-silent somatic single nucleotide variants (SNVs) and insertion and deletions (indels) at diagnosis and/or relapse(s) were identified using the MutSigCV^14^ and OncodriveFML^15^ software. Genes were defined as drivers of ALL in diagnostic and/or relapse samples based on somatic mutations (SNVs and indels) if : (1) at least one of the bioinformatic tools MutSigCV^14^ or Oncodrive-fml^15^ reported a gene as a potential driver with a p-value <  = 0.05; and (2) the mutations were recurrent among the ALL patients ; and (3) the mutations were putative non-silent somatic variants; and (4) the genes had clonal allelic variants with AF >  = 0.25; and (5) the mutant allele of a SNV was expressed at a detectable level in RNA in at least one of the same sample that carried the putative functional variant. For genes already known as ALL driver genes in the literature we required that the criteria 3–5 were fulfilled.

### Detection of non-coding somatic single nucleotide variants

Putative regulatory non-coding somatic mutations were annotated to predicted regulatory genomic regions with histone marks for active promoters (H3K4me3), active enhancers (H3K4me1, H3K27ac), or regions of open chromatin (DNase1 hypersensitivity) or transcription factor binding sites in the lymphoblastoid cell line GM12878 in the ENCODE^25^ database and/or in four primary ALL blasts cells from the BLUEPRINT project^24^. In addition to the criteria listed in the section “Detection of somatic point mutations” above, the genome sequences of the remission (germline) samples from those 18 patients who carried candidate non-coding somatic variants were compared to the Genome Aggregate Database (GnomAD v2.1.1)^46^ which contains quality controlled WGS data from 12 populations. The variant alleles of the putative non-coding somatic variants in the 18 patients were not present in 7800 samples from the European population in the GnomAD v2.1.1 database. The absence of the somatic variant alleles in the large set of population samples provides further evidence for the somatic nature of the somatic candidate regulatory non-coding variants. The somatic candidate variants were evaluated for evolutionary conservation based on overlaps with the PhastCons score using the “phastConsElements46way” tool in the UCSC browser^47^. To identify non-protein coding regions with recurring mutations, candidate variants were clustered hierarchically, and partitioned to have a maximal distance between variants within a cluster smaller than 100 bp. For the non-coding mutations in Supplementary Table S4, we applied the cis-X tool^26^ on 1 Mb regions surrounding the non-coding variants to calculate the outlier high expression (OHE) and allele specific expression (ASE). For OHE we used the following cis-X results: size of the whole reference cohort, cohort rank and p-value in outlier test using the whole reference cohort. For each candidate gene in Supplementary Table S4, ASE was evaluated based on the following cis-X results: number of heterozygous markers in each gene and sample, number of heterozygous markers showing ASE per sample, combined p-value for the ASE test and average variant allele frequency difference between RNA and DNA for all markers. For the ASE analysis in our samples, we were not able to generate an expression reference with a sufficient number of biallelic candidates for the T-ALL samples, and we therefore resorted to using the T-ALL expression reference provided with cis-X instead of our own T-ALL samples.

### Detection of structural variants

Somatic copy number alterations (CNAs) ranging from ~ 1 kb to full length chromosome arms and complete chromosomes were identified using the Allele-Specific Copy number Analysis of Tumors (ASCAT) software v2.5^48^, applying the “gc” correction and “aspcf” segmentation to define complete digital karyotypes. LogR and B-allele frequency (BAF) values were computed using AlleleCount version 2.2.0. Large-scale somatic structural variants (SVs) ranging from 50 bp to several Mb in size were identified using the Manta software^42^, which uses the split-read and read-pair orientation for the detection of translocations, inversions, deletions and duplications. The somatic SVs were further filtered out (1) if they were present in the SweGen^43^ database of normal variation with 50% fraction overlap; or (2) if they overlapped any 10 kb genomic window with average coverage < 10 reads in the matched normal sample or in the UCSC assembly gap regions; or (3) if a deletion overlapped a duplication in the same sample. SVs were annotated with the Ensembl Variant Effect Predictor (VEP)^45^. SVs that overlap the T-cell receptor, human leukocyte antigen and immunoglobulin gene (*TARP HLA IGH*) were removed. SVs with known driver genes or fusion genes according to the ALL literature were not removed using the above filtering criteria.

The identified large-scale CNVs and SVs were overlaid with the tumor suppressor genes and oncogenes present in the OncoKB database^49^, followed by manual inspection of the identified candidates for coverage of heterozygous and homozygous deletions in the Integrated Genome Browser (IGV) to identify affected cancer genes^50^.

### RNA sequencing

RNA-sequencing (RNA-seq) libraries were prepared from RNA available for 22 ALL patients at diagnosis, 25 patients at first relapse, seven patients at second relapse and from control B-cells (CD19 +) and T-cells (CD3 +) from five healthy Swedish individuals (Supplementary Table S7)^51^. Depending on the quality (RIN > 7) and amount of available RNA (> 100 ng), the TruSeq stranded total RNA protocol (Illumina, Inc.) was applied with 300 ng of input RNA. For the remaining samples, the TruSeq RNA access protocol was used with 30–100 ng of input RNA (Illumina Inc.). Thirty million sequence read pairs (paired-end, 65 bp) per sample were generated per library on a HiSeq2500 instrument followed by mapping to the human reference genome hg19 using STAR^52^. For putative variants located in driver genes or genes flanking putative regulatory non-coding variants, gene expression levels were calculated in the samples containing the mutation and the log_2_ of a FPKM (fragments per kilobase per million mapped reads) value > 1 was used as a criterion for gene expression. To detect allele-specific expression of mutant alleles we used GATK for SNVs to calculate the mutant allele frequency and confirmed expression of the mutant allele manually for indels using IGV in RNA data^39,50^.

### Assignment of clonal evolution patterns

The somatic SNVs were clustered based on their allele frequency (AF) at diagnosis and relapse(s) using the PyClone software v0.13.1^53^. PyClone parameters were set according to PyClone manual and default recommendations (https://github.com/Roth-Lab/pyclone). For density estimates, PyClone_beta_binomial was used. Tumor purities were estimated by ASCAT. Cluster and locus tables were prepared with the maximal number of clusters set to 10, except for ALL_832 where 11 was used. Clonal models were built using the ClonEvol software v0.99.10^54^ in the R package for clonal ordering, visualization and interpretation. AFs were adjusted manually to correct for tumor purity and clusters were manually adjusted to fit the most probable parent clusters (Supplementary Fig. S4). Functionally important somatic indels, CNAs and SVs were assigned manually to the clonal evolutionary trees. Finally, we used previously generated targeted sequencing data at an average 600 × coverage (Haloplex)^19^, which was available from 16 of our 29 diagnostic samples to validate the trajectory assignments.

### Ethics approval and consent to participate

The study was performed in accordance with guidelines of the Helsinki Declaration. The study was approved by the Regional Ethical Board in Uppsala, Sweden (Approvals Dnr 2010/128; Dnr 2013/237; Dnr 2014/233). The samples were obtained with written informed consent for genetic research from the parents of the patients and/or the patients.

### Consent for publication

All authors have read the manuscript and approved its submission for publication.

## Supplementary Information

Supplementary Information 1.

Supplementary Information 2.

Supplementary Information 3.

## Data Availability

A list of all somatic variants detected in WGS is provided as Supplementary data and a gene expression count matrix for RNA data is available at the Gene Expression Omnibus (GEO) with accession number GSE163634. Additional data is available from the corresponding authors (A-CS, SS) on reasonable request.
