# Supplementary material for: Mutational patterns and clonal evolution from diagnosis to relapse in pediatric acute lymphoblastic leukemia
Source: Sci Rep. 2021 Aug 6;11:15988. doi: 10.1038/s41598-021-95109-0 (PMC8346595; doi:10.1038/s41598-021-95109-0)
Supplement: Supplementary file 2 — Supplementary Information 2. [file 41598_2021_95109_MOESM2_ESM.pdf]

# Mutational patterns and clonal evolution from diagnosis to relapse in pediatric acute lymphoblastic leukemia

Shumaila Sayyab[1*], Anders Lundmark[1], Malin Larsson[2], Markus Ringnér[3], Sara Nystedt[1], Yanara Marincevic-Zuniga[1], Katja Pokrovskaja Tamm[4], Jonas Abrahamsson[5,11], Linda Fogelstrand[6,7,11], Mats Heyman[8,11], Ulrika Norén-Nyström[9,11], Gudmar Lönnerholm[10], Arja Harila-Saari[10,11], Eva C. Berglund[1], Jessica Nordlund[1], Ann-Christine Syvänen[1*]

[1]Department of Medical Sciences, Molecular Medicine and Science for Life Laboratory, Uppsala University, Uppsala, Sweden

[2]Department of Physics, Chemistry and Biology, National Bioinformatics Infrastructure Sweden, Science for Life Laboratory, Linköping University, Linköping, Sweden

[3]Department of Biology, National Bioinformatics Infrastructure Sweden, Science for Life Laboratory, Lund University, Lund, Sweden

[4]Department of Oncology-Pathology, Karolinska Institutet, Stockholm, Sweden

[5]Department of Pediatrics, Institute of Clinical Sciences, Sahlgrenska Academy at University of Gothenburg, Gothenburg, Sweden

[6]Department of Laboratory Medicine, Institute of Biomedicine, Sahlgrenska Academy at University of Gothenburg, Gothenburg, Sweden

[7]Department of Clinical Chemistry, Sahlgrenska University Hospital, Gothenburg, Sweden

[8]Childhood Cancer Research Unit, Karolinska University Hospital, Stockholm, Sweden

[9]Department of Clinical Sciences and Pediatrics, University of Umeå, Umeå, Sweden

[10]Department of Women's and Children's Health, Uppsala University, Uppsala, Sweden

[11]For the Nordic Society of Pediatric Hematology and Oncology

Running head: Mutational patterns and clonal evolution in ALL

*corresponding authors: Shumaila Sayyab; Ann-Christine Syvänen

Molecular Medicine, Department of Medical Sciences

Uppsala University, Biomedical Centre

Box 1432, 75144 Uppsala, Sweden

Telephone: +46 728588905 (SS), +46 707813411 (A-CS)

Email: shumaila.sayyab@medsci.uu.se; Ann-Christine.Syvanen@medsci.uu.se

The authors have no competing interests to declare

**Supplementary File**

**Extended figure legends**

**Figure 5 a-f**

**Figure 6 a-f**

**Legends to Figs. S1-S6**

**Supplementary Figs. S1-S6**

## Extended legends for Fig. 5 and Fig. 6

**Figure 5. Clonal evolution from diagnosis to relapse representative patients illustrating the "persistent clone" and "founding clone" trajectories.** For each patient panels a, e and f, section (i) show the clones and subclones identified at diagnosis, first and second relapse, where clone 1 in (grey) represents the founding clone. The height of the colored fields corresponds to the proportion of the clones in a sample. Section (ii) shows the clones and subclones identified at diagnosis (Di), first (R1) and second relapse (R2). Each color-coded branch shows the known and putative driver genes identified at each time point, with the gene names highlighted in blue for fusion genes and in red for putative regulatory non-coding variants. Section (iii) shows the total number of somatic SNVs present in each clone using the same color code as in sections (i) and (ii).

**Figure 5a.** Consensus model for clonal evolution in ALL_128 (B-other group) illustrating the "persistent clone trajectory". Clone 1 (grey) displays a heterozygous deletion of *PLAA*, a ~32.5 kb heterozygous deletion (del) spanning the *SH2B3* gene, which combined with somatic frameshift (fs) and missense mutations (ms) in *SH2B3* cause biallelic loss of function, frameshift mutations in *ETV6*, and missense mutations in *BIRC7*. The minor subclone 7 (red) present at diagnosis disappeared at R1. At R1, two novel clones have appeared. Subclone 4 (purple) contains homozygous deletions of *CDKN2A* and ~50 kb deletion of exons 3-7 of the *IKZF1* gene, and heterozygous deletion of *NF1*and *PMS2*. This clone persists and expands at R2. Heterozygous deletion of the mismatch repair gene *PMS2* on chr7p at R1, which becomes homozygous at R2 likely leads to the hypermutated descendant clone 3 (green).

**Figure 5b.** Sequence coverage plot for the *SH2B3* gene in ALL_128 with a ~35.2 kb heterozygous deletion (Di, orange line; R1, blue line; R2, purple line). The relative coverage in

the leukemic samples (Di, R1 and R2) is 0.5 compared to the remission (germline) DNA sample with a coverage of 1 (green line).

**Figure 5c.** Sequence coverage plot for the *IKZF1* gene in ALL_128. The coverage plot shows a ~50 kb homozygous deletion of exons 3-7 in *IKZF1* with relative coverage of 0 at R1 and R2 (blue and purple lines), compared to relative coverage of 1 in the diagnosis (orange line) and remission (green line) samples.

**Figure 5d.** Coverage plot for the *PMS2* gene in patient ALL_128. The region containing a heterozygous deletion in *PMS2* is detected at R1 and a homozygous deletion at R2.

**Figure 5e.** Consensus model for clonal evolution in the patient ALL_832 (HeH subtype) illustrating the "persistent clone trajectory". The founding clone 1 (grey) with 52 chromosomes contains gains of six complete chromosomes (+4, +8,+9,+14,+21,+X ). Subclone 4 (purple) at containing 218 somatic mutations at Di, disappears at R1 while the main clone 5 (pink) gains mutations in the *CREBBP* gene, which remain at R2. Clone 5 gives rise to two new subclones (#2, blue and #9, brown). Subclone 2 disappears at R2, while subclone 9 containing 104 (new) mutations expands at R2. Subclone 9 gives rise to two new subclones (#7, red and #3, light green), which harbor a new *NRAS* mutation.

**Figure 5f.** Consensus model for clonal evolution in patient ALL_109 with the t(12;21) *ETV6-RUNX1* subtypes illustrates the "founding clone trajectory". The expressed driver fusion gene *ETV6-RUNX1* is present in the founding clone 1 (grey). Two subclones present at Di (#10, green and #3, light green) disappear at first relapse, while the main clone 6 (orange) containing a missense mutation in *HDAC2* persists at R1. At R1 a missense mutation in *NT5C2,* a stop-gain mutation in *CREBBP* and putative mutation in the intron of *GATAD2B* appear in clone 4 (purple), but this clone disappears at the R2. Subclone 6 with a missense mutation in *HDAC2*

gives rise to subclone 7 (red) with a missense mutation in *PRPS1*. Both subclones 6 and 7 expand at second relapse, giving rise to subclone 2 (blue) with a homozygous deletion (del) in *BCORL1*.

**Figure 6. Consensus model for clonal evolution from diagnosis to relapse for six ALL patients of different subtype in the "rising clone trajectory".** The sections denoted (i-iii) are as described in the legend of Fig. 5.

**Figure 6a.** Consensus model for clonal evolution in patient ALL_257 (*ZNF384*-rearranged). The founding clone 1 (grey) displays heterozygous deletions (del) and frameshift (fs) deletion of *ETV6* gene causing a biallelic affect and homozygous deletion of the regions containing *CDKN2A*, together with missense mutations (ms) in the *CREBBP* gene and frameshift (fs) indels in *ZNF384* and *UBA2* genes. At least three subclones (#2, blue, #5, pink and #8, green) were present at Di. Subclones 5 and 8 disappear at R1, while subclone 2, expands at R1 giving rise to subclone 3. In main clone 9 at R1 a second mutation causing a stop-gain mutation resulting in premature truncation of the encoded protein arises in the *CREBBP* gene along with a putative regulatory mutation in intron 5 of the *MSI2* gene. A new subclone (#7, red) appears at R1, containing a putative regulatory mutation in the enhancer region located ~24 kb downstream of *ZNF648* and ~222 kb downstream of *CACNA1E*.

**Figure 6b.** Consensus model for clonal evolution in patient ALL_244 (*MED2F-BCL9*) illustrating the "rising clone trajectory". The founding clone 1 (grey) has a non-recurrent rearrangement involving heterozygous deletions (del) of the regions containing the *IKZF1* and *NR3C1* genes and an inversion of ~9MB on chr 1 (inv1) that results in the expressed *MEF2D-BCL9* fusion gene. A mutation in the *KRAS* driver gene is observed in the major clone 6 (orange) at Di, which disappears at R1. Subclone 7 (red) at Di containing a driver mutation in *EP300*

expands at R1 and persists at R2. Subclone 3 (green), which appears at R1 expands at R2 by acquiring new mutations in the *PLAA* gene. Subclone 4 (purple), which appears at R1and contains a mutation at *CREBBP* has disappeared at second relapse.

**Figure 6c.** Consensus model for clonal evolution in patient ALL_680 (dic(9;20) subtype). This patient shows founding clone 1 containing t(2;7)(p12;p12) with an expressed fusion gene *IKZF1-IGK*, a homozygous deletion (del) in *CDKN2A*, a heterozygous deletion (del) of *PLAA*, and a non-coding mutation in the intron 1 of the *CSK* gene. At Di, the main clone 4 containing a mutation in *KRAS* and two subclones (#3, color and #8, color) disappear at R1. However, subclone 10 (dark green) present at Di expands at R1 and this clone gives rise to a new subclone 5 (color) with a homozygous deletion (del) of the *NF1* gene.

**Figure 6d.** Consensus model for clonal evolution in patient ALL_5 (*KMT2A*-rearranged). The canonical fusion gene *AFF1-KMT2A* is present in the founding clone. The main clone (#4, purple) at Di disappears at R1, while subclone 6 (orange) with heterozygous deletions in *EBF1* and *ZCCHC7* expands at R1 and persists at R2. At R1 the expanded clone 6 forms the major clone giving rise to three new subclones (#2, blue; #5, pink #8, green). Of these, subclone 5 expands R2, while subclones 2 and 8 disappear. Subclone 5 harbors a heterozygous deletion in *CDKN2A* gene. At R2, subclones 5 and 6 give rise to a new subclone (#3, light green).

**Figure 6e.** Consensus model for clonal evolution in patient ALL_358 (T-ALL). A homozygous deletion in the *CDKN2A* gene and a missense mutation in *HIST1H4J* are observed in the founding clone 1 (grey). The major subclone 6 (orange) at Di contains somatic mutations in the driver genes *FBXW7* and *NOTCH1* and a putative intronic of SENP8/*MYO9*, all of which disappear at R1. Subclone 4 (purple) at Di contains missense mutations in *IKZF1* and *NRAS,* which expand at R1 forming the major clone. An additional subclone 2 containing a missense

mutation in the driver gene *NT5C2,* a stop-gain mutation in *ARID1A,* a frameshift in *NOTCH1,* a heterozygous deletion in *FBXW7* and a regulatory mutation in *PALM2-AKAP2* appear at R1, while subclone 3 (green) with a frameshift mutation and heterozygous deletion in *IKZF1* are observed.

**Figure 6f**. Consensus model for clonal evolution in patient ALL_827 (MPAL). The founding clone 1 (grey) has the t(7;12) translocation resulting in the expression of the *ETV6-TSL1* fusion gene. The major clone 9 (brown) with an *NRAS* mutation at Di disappears at R1, while subclone 4 (purple) present at Di expands at R1 acquiring new mutations in subclone 2 (blue) and subclone 3 (green).

## Supplementary Figs. S1-S6

**Supplementary Fig. S1. Determination of mutational trinucleotide signatures in 67 ALL genomes.**

**(a)** *De novo* mutational signatures were extracted from the mutations identified in the ALL patient samples using non-negative matrix factorization (NMF). The parameter describing the number of mutational signatures (the factorization rank, horizontal axis) were evaluated using the residual sum of squares (RSS) quality criterion (vertical axis). With a given set of mutational signatures, the mutations identified in the samples can be reconstructed. RSS is a measure of the difference between the reconstructed mutations and the identified mutations, and hence it is a measure of how well all mutations in the patient samples are reconstructed using the mutational signatures. In the plot, RSS decreases substantially when the factorization rank is increased from 2 to 3, while the RSS decreases relatively little from the factorization rank 3 to higher ranks. Based on the RSS plot and by inspection of the resulting mutational signatures with different numbers of signatures, we chose to extract three mutational signatures for our analyses. To robustly extract more than three mutational signatures would most likely require more than 67 ALL samples.

**(b)** Three *de novo* mutational signatures identified based on the mutations in the 67 ALL genomes. The trinucleotide context of the six possible somatic mutation types are shown on the horizontal axis and the relative contributions of the trinucleotide signatures are shown on the vertical axis.

**(c)** Cosine similarities (vertical axis) between observed mutations and mutations reconstructed from different sets of COSMIC mutational signatures (horizontal axis) for the 67 ALL genomes. The three *de novo* mutational signatures identified in our sample set and twelve COSMIC

signatures that had a cosine-similarity > 0.65 to one of the three *de novo* signatures reconstruct the observed mutations well (median cosine similarities of 0.95 and 0.94, respectively). The six signatures (SBS1, SBS2, SBS6, SBS13, SBS40 and SBS89) reconstruct the observed mutations with a median similarity =0.93, which was not significantly different compared to the three *de novo* signatures (*t*-test, $p$ = 0.17). Therefore, we used these six signatures in our analyses.

**(d)** Cluster analysis of the three *de novo* mutational signatures (vertical-axis) based on their cosine similarity to twelve known COSMIC signatures (horizontal-axis). Cosine-similarities between *de novo* mutational signatures in the ALL patients and the COSMIC signatures are shown. Twelve COSMIC signatures had a cosine-similarity > 0.65 to one of the three *de novo* signatures in ALL and were used in the analysis.

**(e)** Hierarchical clustering of twelve COSMIC signatures. The average agglomeration method and a distance measure (height) based on cosine similarity were used.

# Figure S1

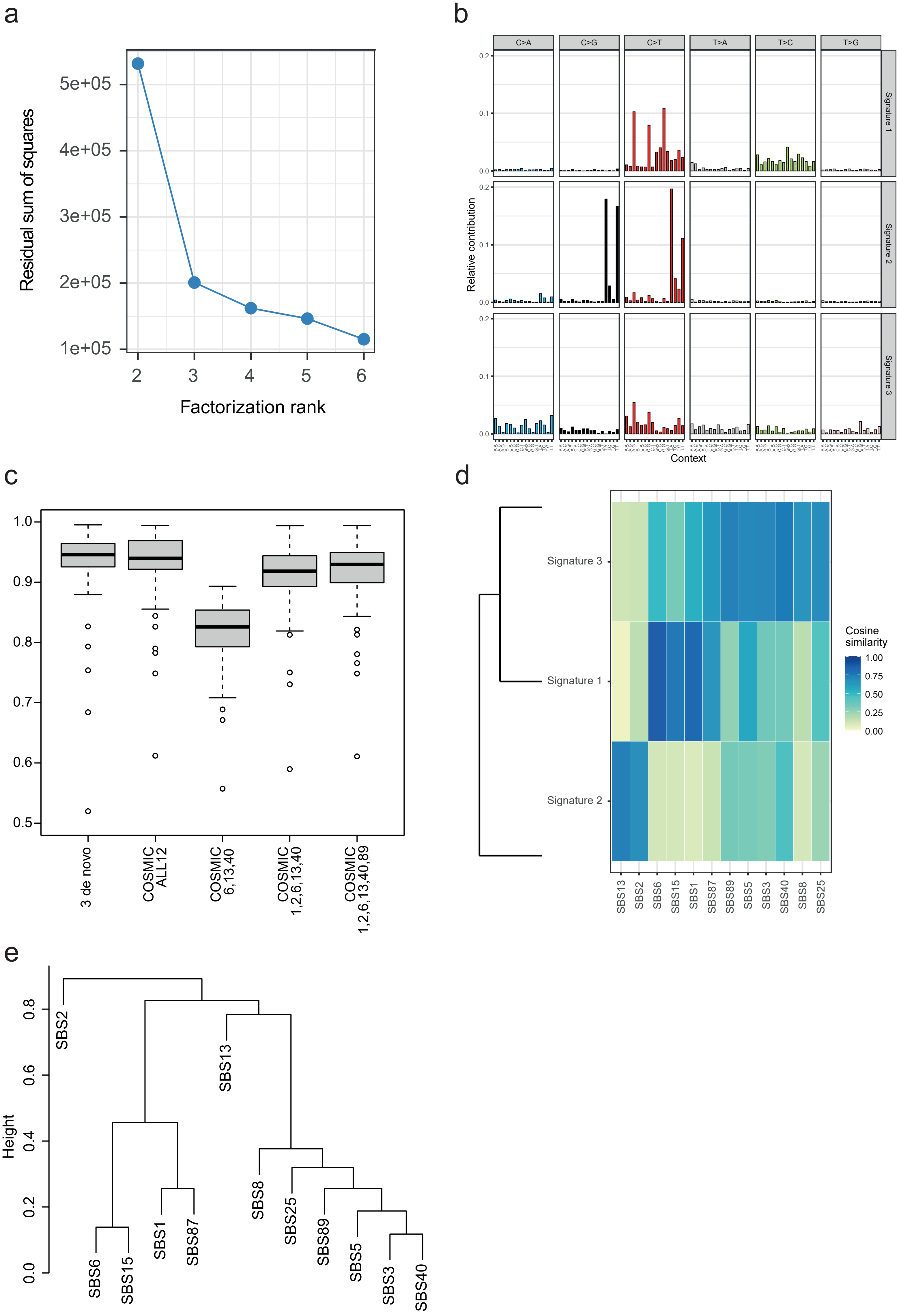

a
Residual sum of squares
5e+05
4e+05
3e+05
2e+05
1e+05
2
3
4
5
6
Factorization rank
b
C>A
C>G
C>T
T>A
T>C
T>G
Signature 1
Signature 2
Signature 3
Relative contribution
0.2
0.1
0.0
Context
c
1.0
0.9
0.8
0.7
0.6
0.5
3 de novo
COSMIC ALL12
COSMIC 6,13,40
COSMIC 1,2,6,13,40
COSMIC 1,2,6,13,40,89
d
Signature 3
Signature 1
Signature 2
Cosine similarity
1.00
0.75
0.50
0.25
0.00
SBS13
SBS2
SBS6
SBS15
SBS1
SBS87
SBS89
SBS5
SBS3
SBS40
SBS8
SBS25
e
Height
0.8
0.6
0.4
0.2
0.0
SBS2
SBS13
SBS6
SBS15
SBS1
SBS87
SBS8
SBS25
SBS89
SBS5
SBS3
SBS40

**Supplementary Fig. S2. Distribution of non-silent somatic mutations (SNVs, indels) in putative ALL driver genes**

**(a)** Putative driver genes containing non-silent point mutations (SNVs and indels) in the major clones and subclones in 29 patients with ALL. The vertical columns in the upper panel show the ALL samples from each patient with vertical lines to separate the samples at diagnosis (Di), first relapse (R1), and second relapse (R2) from each patient. The insert in the figure is a color code for putative driver genes detected at Di (blue), R1/R2 (pink) and both at Di and R1/R2 (grey). The genes with recurrent somatic mutations are shown with black letters on the left side of the panel and genes previously known in ALL from the literature and identified in a single patient in our study are shown with blue letters. Subclonal mutations with allele frequency (AF) < 0.25 are also included. The genetic subtypes of the patients are shown in the lower panel.

**(b)** AF distribution of non-silent somatic mutations (SNVs, indels) in putative ALL driver genes. Each of the 28 panels shows a driver gene containing somatic mutations (SNV, indicated by a dot, indel as a triangle) in ALL patients. The change in AF in each sample is shown by a line connecting the AFs at diagnosis and relapse(s). The driver genes observed in a single patient are shown in blue font. Subclonal mutations with an AF < 0.25 are also shown. The AF for the mutations are shown on the vertical -axis in each panel and the-horizontal axis in each panel indicates samples at Di, R1 and R2.

**(c)** Expression of the mutant allele of putative driver genes in the ALL samples. The vertical columns in the upper panel show the samples from each ALL patient with vertical lines to separate the samples at Di, R1 and D2 from each patient. The horizontal rows in the top panel indicate the driver genes detected using somatic mutations (SNVs as circle and indels as triangle). Each colored box represents the mutant allele frequency of SNVs in the RNA-

sequencing data in the sample containing the mutation according to the color key on the right side of the panel. For indels, the allele frequency is not provided and the expression of the mutant allele was manually confirmed in the integrated genome viewer (IGV) and shown with a colored box (green with triangle). The grey boxes represent very low mutant allele frequencies (0-0.05), to indicate that the reference (non-mutated) allele is detected at that position. Sample where RNA sequencing data is not available are shown with white columns. The vertical rows in the bottom panel (grey) show genetic subtypes. The driver genes observed in single patients are shown in blue font on the left side of the panel.

# Figure S2a

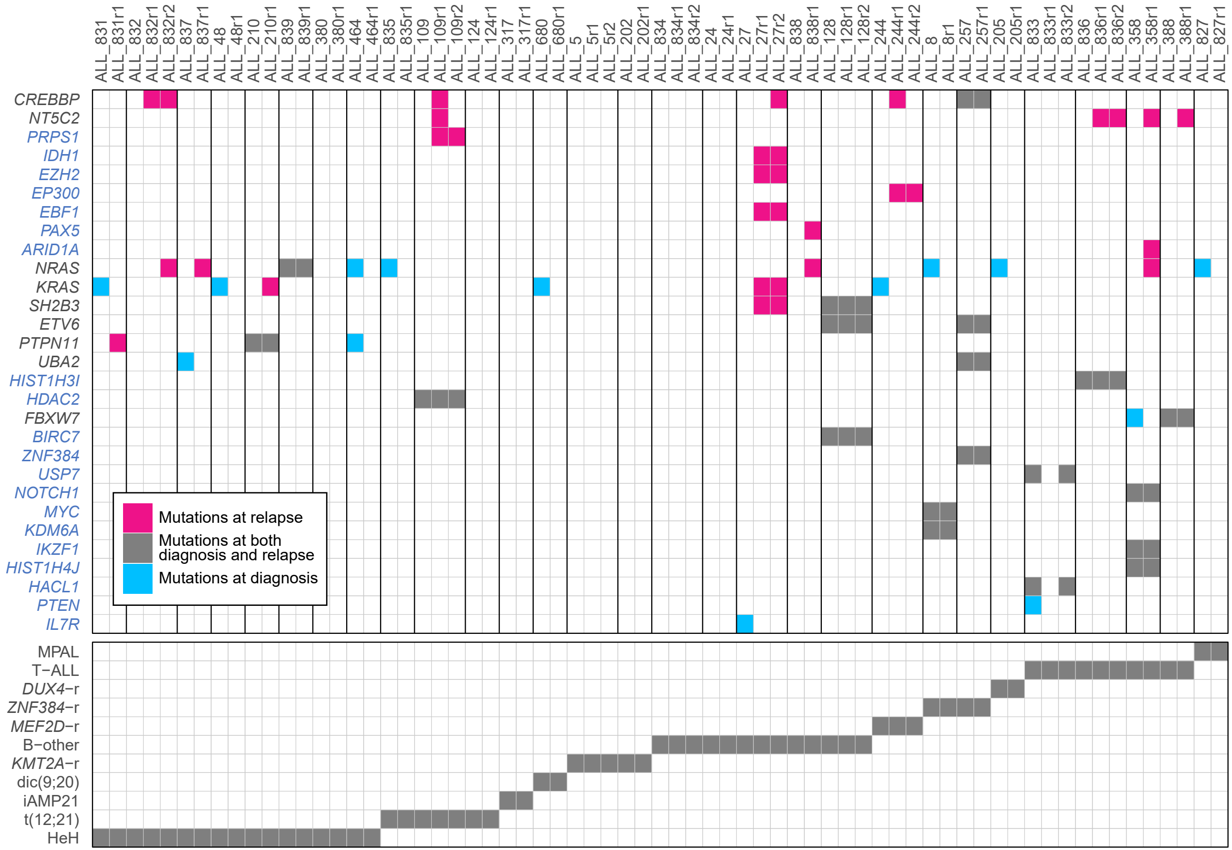

ALL_831
ALL_831r1
ALL_832
ALL_832r1
ALL_832r2
ALL_837
ALL_837r1
ALL_48
ALL_48r1
ALL_210
ALL_210r1
ALL_839
ALL_839r1
ALL_380
ALL_380r1
ALL_464
ALL_464r1
ALL_835
ALL_835r1
ALL_109
ALL_109r1
ALL_109r2
ALL_124
ALL_124r1
ALL_317
ALL_317r1
ALL_680
ALL_680r1
ALL_5
ALL_5r1
ALL_5r2
ALL_202
ALL_202r1
ALL_834
ALL_834r1
ALL_834r2
ALL_24
ALL_24r1
ALL_27
ALL_27r1
ALL_27r2
ALL_838
ALL_838r1
ALL_128
ALL_128r1
ALL_128r2
ALL_244
ALL_244r1
ALL_244r2
ALL_8
ALL_8r1
ALL_257
ALL_257r1
ALL_205
ALL_205r1
ALL_833
ALL_833r1
ALL_833r2
ALL_836
ALL_836r1
ALL_836r2
ALL_358
ALL_358r1
ALL_388
ALL_388r1
ALL_827
ALL_827r1
CREBBP
NT5C2
PRPS1
IDH1
EZH2
EP300
EBF1
PAX5
ARID1A
NRAS
KRAS
SH2B3
ETV6
PTPN11
UBA2
HIST1H3I
HDAC2
FBXW7
BIRC7
ZNF384
USP7
NOTCH1
MYC
KDM6A
IKZF1
HIST1H4J
HACL1
PTEN
IL7R
Mutations at relapse
Mutations at both diagnosis and relapse
Mutations at diagnosis
MPAL
T−ALL
DUX4−r
ZNF384−r
MEF2D−r
B−other
KMT2A−r
dic(9;20)
iAMP21
t(12;21)
HeH

# Figure S2b

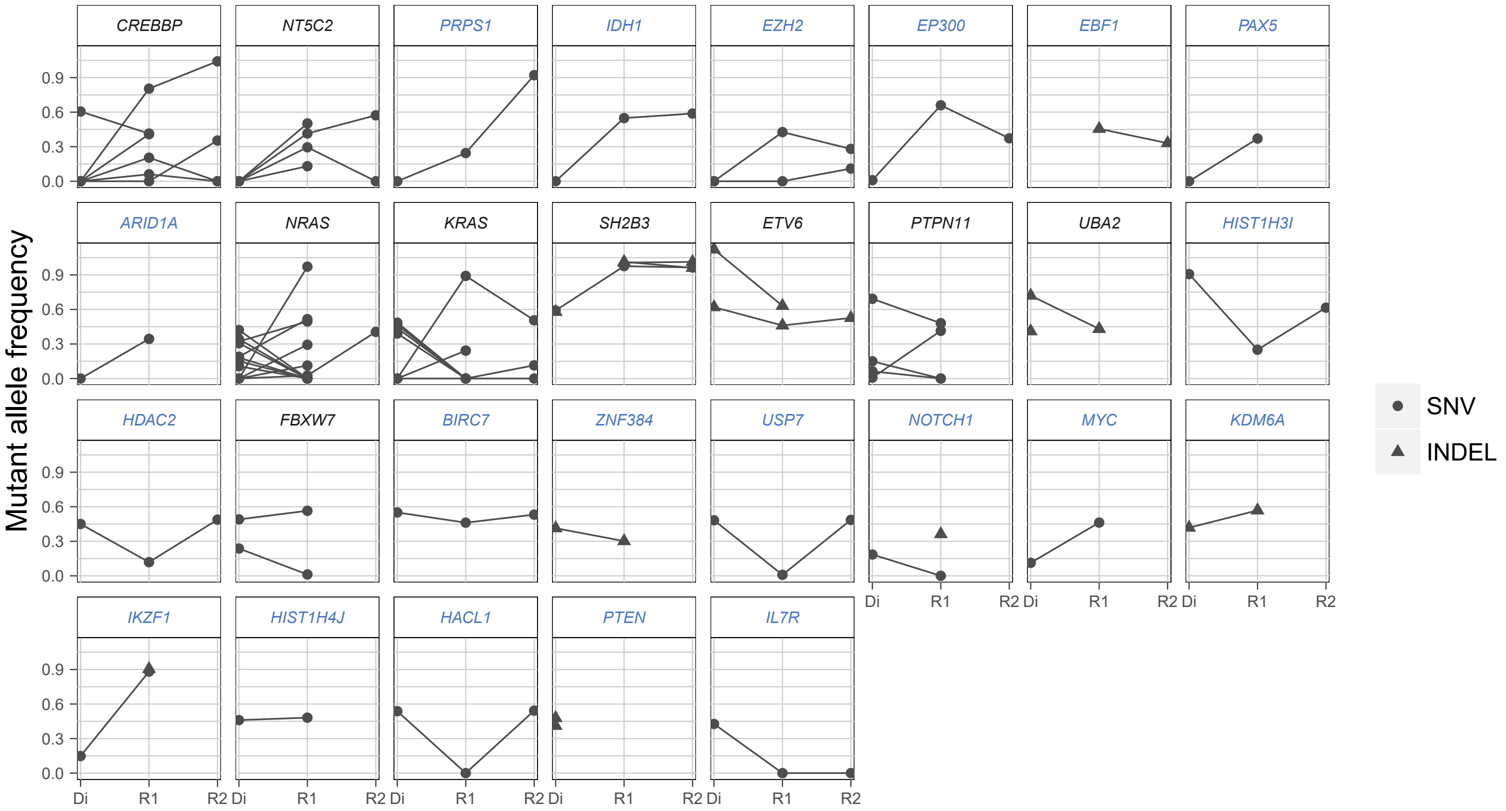
CREBBP
NT5C2
PRPS1
IDH1
EZH2
EP300
EBF1
PAX5
ARID1A
NRAS
KRAS
SH2B3
ETV6
PTPN11
UBA2
HIST1H3I
HDAC2
FBXW7
BIRC7
ZNF384
USP7
NOTCH1
MYC
KDM6A
IKZF1
HIST1H4J
HACL1
PTEN
IL7R
Mutant allele frequency
0.0
0.3
0.6
0.9
Di
R1
R2
SNV
INDEL

# Figure S2c

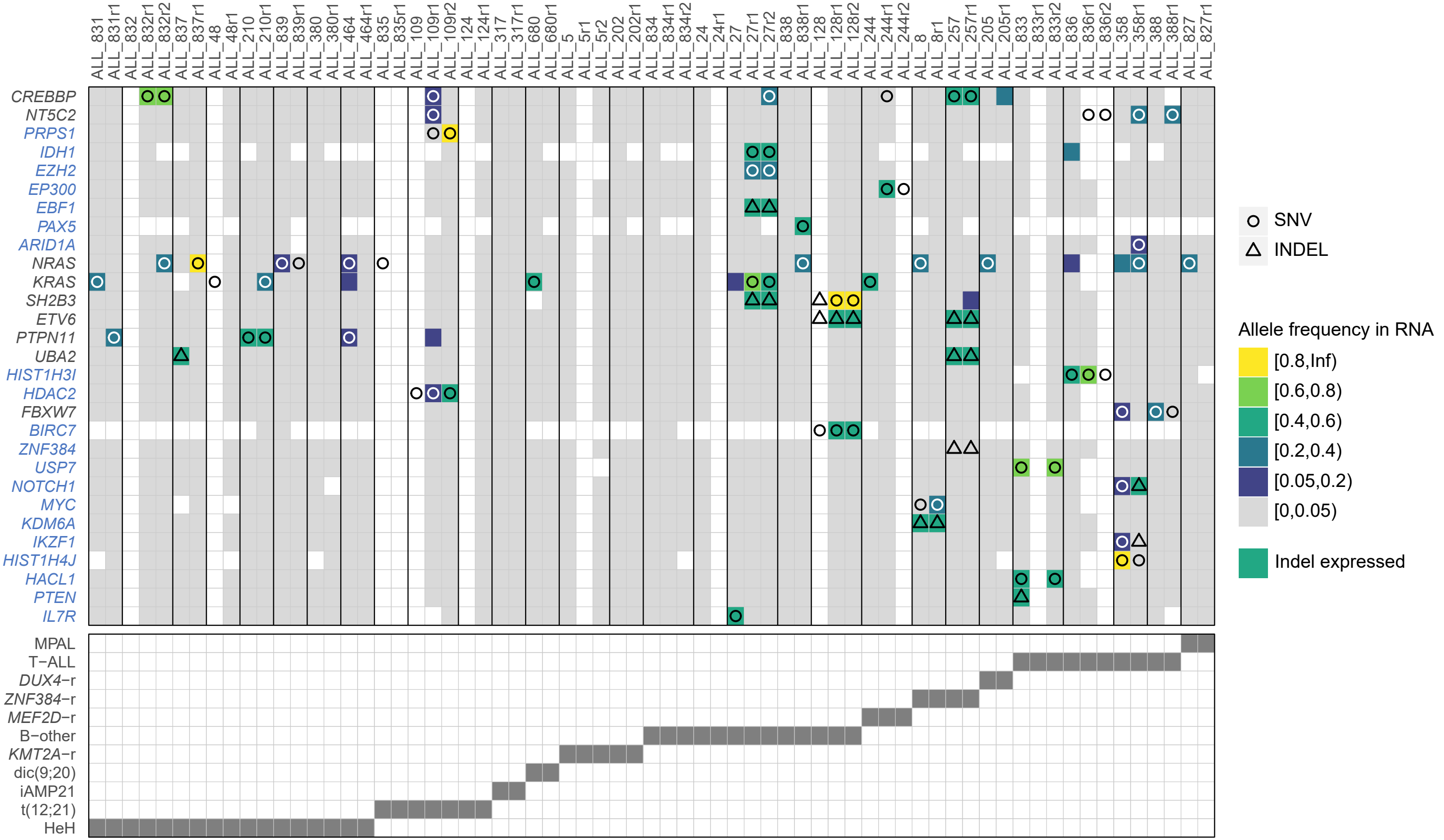
ALL_831
ALL_831r1
ALL_832
ALL_832r1
ALL_832r2
ALL_837
ALL_837r1
ALL_48
ALL_48r1
ALL_210
ALL_210r1
ALL_839
ALL_839r1
ALL_380
ALL_380r1
ALL_464
ALL_464r1
ALL_835
ALL_835r1
ALL_109
ALL_109r1
ALL_109r2
ALL_124
ALL_124r1
ALL_317
ALL_317r1
ALL_680
ALL_680r1
ALL_5
ALL_5r1
ALL_5r2
ALL_202
ALL_202r1
ALL_834
ALL_834r1
ALL_834r2
ALL_24
ALL_24r1
ALL_27
ALL_27r1
ALL_27r2
ALL_838
ALL_838r1
ALL_128
ALL_128r1
ALL_128r2
ALL_244
ALL_244r1
ALL_244r2
ALL_8
ALL_8r1
ALL_257
ALL_257r1
ALL_205
ALL_205r1
ALL_833
ALL_833r1
ALL_833r2
ALL_836
ALL_836r1
ALL_836r2
ALL_358
ALL_358r1
ALL_388
ALL_388r1
ALL_827
ALL_827r1
CREBBP
NT5C2
PRPS1
IDH1
EZH2
EP300
EBF1
PAX5
ARID1A
NRAS
KRAS
SH2B3
ETV6
PTPN11
UBA2
HIST1H3I
HDAC2
FBXW7
BIRC7
ZNF384
USP7
NOTCH1
MYC
KDM6A
IKZF1
HIST1H4J
HACL1
PTEN
IL7R
MPAL
T−ALL
DUX4−r
ZNF384−r
MEF2D−r
B−other
KMT2A−r
dic(9;20)
iAMP21
t(12;21)
HeH
SNV
INDEL
Allele frequency in RNA
[0.8,Inf)
[0.6,0.8)
[0.4,0.6)
[0.2,0.4)
[0.05,0.2)
[0,0.05)
Indel expressed

**Supplementary Fig. S3. Alignment plots showing RNA-sequencing and WGS reads supporting the *ETV6-TSL* fusion gene and genomic breakpoints in ALL_827.**

**(a)** RNA-seq reads in diagnostic and first relapse samples from the patient ALL_827 mapped to the genome result in split reads (shown in red) between *ETV6* (exon 2) and *TSL* (exon 2). The two panels show one end of the reads mapping to chr7 and the other end of the reads mapping to chr12.

**(b)** Genomic breakpoints for the *t(7;12)* translocation in bam files at diagnosis and first relapse from the patient ALL_827. The WGS data show the genomic breakpoints located at chr7:27536526 (120 kb upstream of *TSL*) and in intron 2 of *ETV6* on chr12:11905675 in ALL_827. The genomic breakpoints are in agreement with the resulting fusion gene detected in the RNA-seq data. The two panels with the read pairs (red color) on chr7 and mate pairs (red color) on chr12 indicate the translocation breakpoints.

# Figure S3a

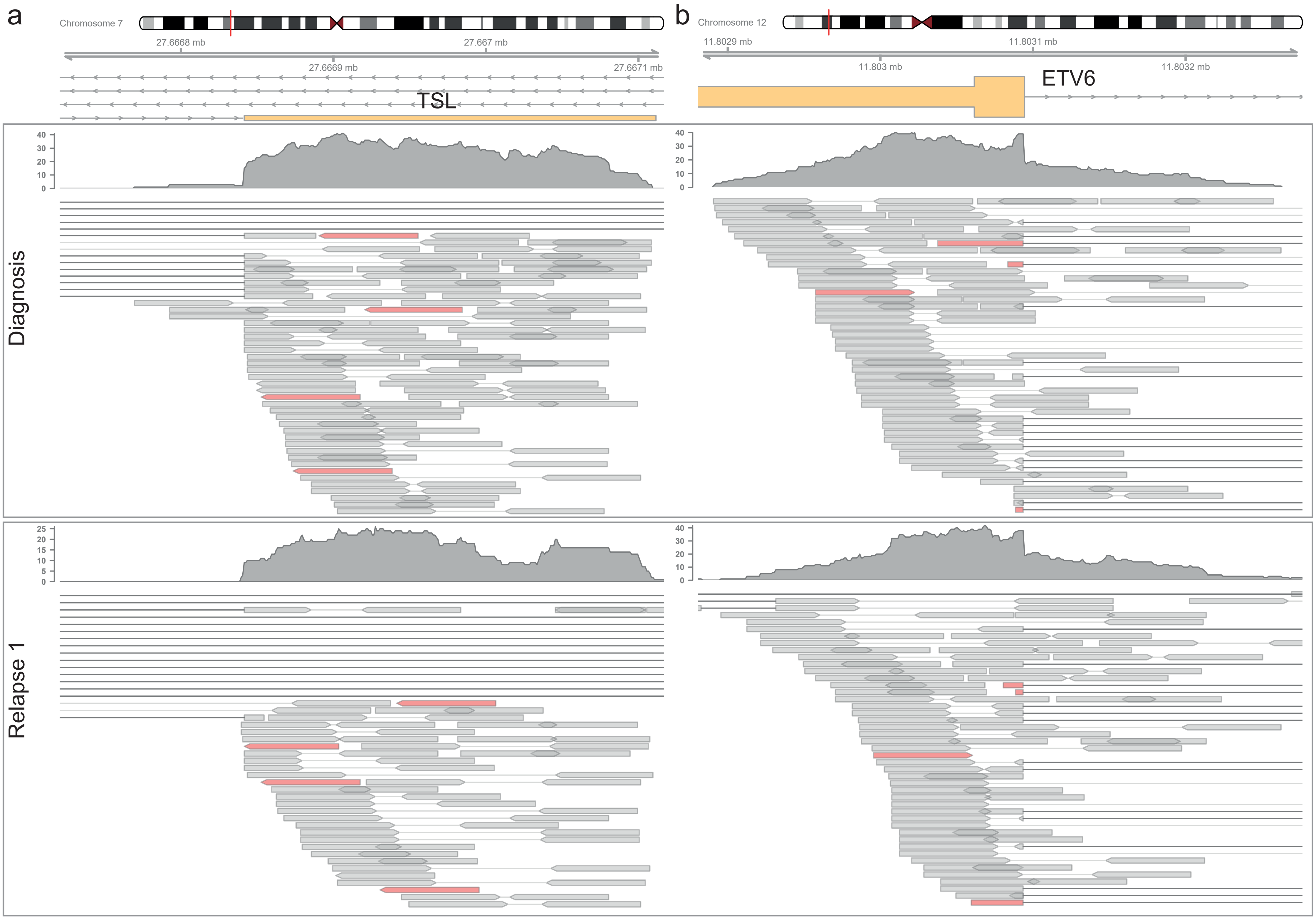
a
Chromosome 7
27.6668 mb
27.667 mb
27.6669 mb
27.6671 mb
TSL
b
Chromosome 12
11.8029 mb
11.8031 mb
11.803 mb
11.8032 mb
ETV6
Diagnosis
Relapse 1

Figure S3b

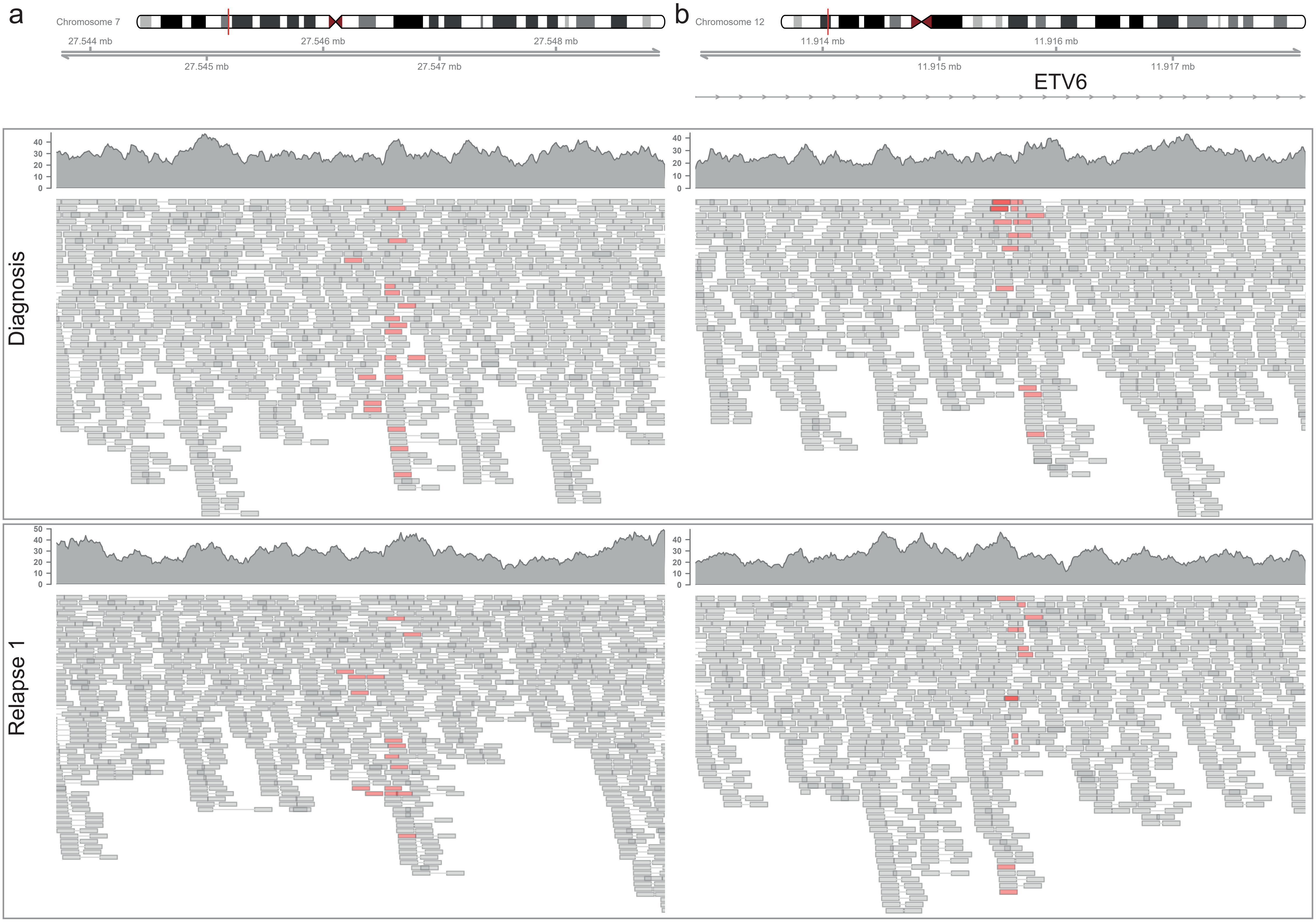

a
Chromosome 7
27.544 mb
27.546 mb
27.548 mb
27.545 mb
27.547 mb
b
Chromosome 12
11.914 mb
11.916 mb
11.915 mb
11.917 mb
ETV6
Diagnosis
Relapse 1

**Supplementary Fig. S4. Overview of the clonal evolution patterns from diagnosis to relapse in 26 ALL patients.** Each page shows the clonal evolution data for a single patient.

**(a)** The left box plot provides the original median variant allele frequencies (VAF) prior to correction for tumor purity and the right hand box plot shows the VAF modeled by Pyclone. The clusters excluded by Pyclone are listed (comma separated) in the top right hand corner. Manual adjustments required to cluster medians are described in the right hand corner of each page by denoting the sample where the adjustment was made (Di, R1, R2) followed by a single space, followed by the cluster id, followed by a colon and the adjustment made in VAF (1-100%). For example: Adjusting cluster medians: Di 4:−1 7:−1, R1 3:−0.5, R2 3:−0.5 means cluster 4 and 7 were shifted 1% down in the diagnostic sample, cluster 3 was shifted down half a percent at first relapse and second relapse

**(b)** The clones and subclones identified at diagnosis, first and second relapse, with the probability of the model given on top, where clone 1 in grey represents the founding clone and the probabilities for the model at diagnosis and relapse(s) are given at each timepoint. The height of the colored fields correspond to the proportion of the clones in a sample.

**(c)** The consensus model tree for the clonal evolution, in which each branch shows the known and putative driver genes identified at diagnosis and first and second relapse.

## Detailed examples to clarify determination of clusters to exclude and manual adjustment of allele frequencies

### Determination of clusters to exclude

For example in Supplementary Fig.S4 for ALL_837, cluster 6 on the x-axis in panel a – Original AF is excluded. Cluster 8 SNVs median VAF at Di is almost as high as that of the founding cluster 1. This means that the SNVs in cluster 6 must belong to a subclone having the SNVs in cluster 8, just like cluster 5, because clones with cluster 8 SNVs fills close to all available space at diagnosis, see panel b, top bell. At R1 the SNVs in cluster 8 and the subclonal SNVs in cluster 5 have disappeared, but cluster 6 remains at the same VAF as at Di. This is not possible if the clone having cluster 6 SNVs is a descendant from a clone with cluster 8 SNVs and model will fail. A closer look at such discrepant clusters revealed a mutational profile with an enrichment of T>G mutations in a G.G context and T>A in an A.A context. When examining the 20 base pairs of sequence surrounding these SNVs we found that they occur in polyA or polyG stretches, which points towards sequencing errors. Another property of such clusters is that their allele frequency seem unaffected by changes in sample tumor cell content. See Supplementary Fig.S4 panel a, with original AF Di, R1 and R2 panels for ALL_109. AF of the founding cluster 1 is low at R1 indicating low tumor content while the allele frequency of cluster 5 remains the same as at Di and R2. In addition to probable artefact clusters (n=52), clusters with very low SNV count (n=51) were excluded.

### Manual adjustment of cluster median allele frequency

For example, see Supplementary Fig.S4, panel a, adjusted AF in ALL_8. This panel is showing data adjusted and modeled by Pyclone before any manual cluster adjustment. Looking closely at adjusted AF in panel R1, the median AF of cluster 2 is higher than the median AF of cluster 3.

This indicates that the clone with cluster 3 SNVs is a descendant of a clone with cluster 2 SNVs, but because cluster 3 SNVs are present at diagnosis, while cluster 2 SNVs are not, this is not possible and model building will fail. We assume that the median AF estimate of cluster 2 is slightly wrong at R2 and therefore we adjust it downwards 3% to make it fit as a descendant of cluster 3, in order to make modeling possible.

# Figure S4

ALL_832 Persistent clone

Excluding clusters: 6, 8, 11, 10
Model: monoclonal

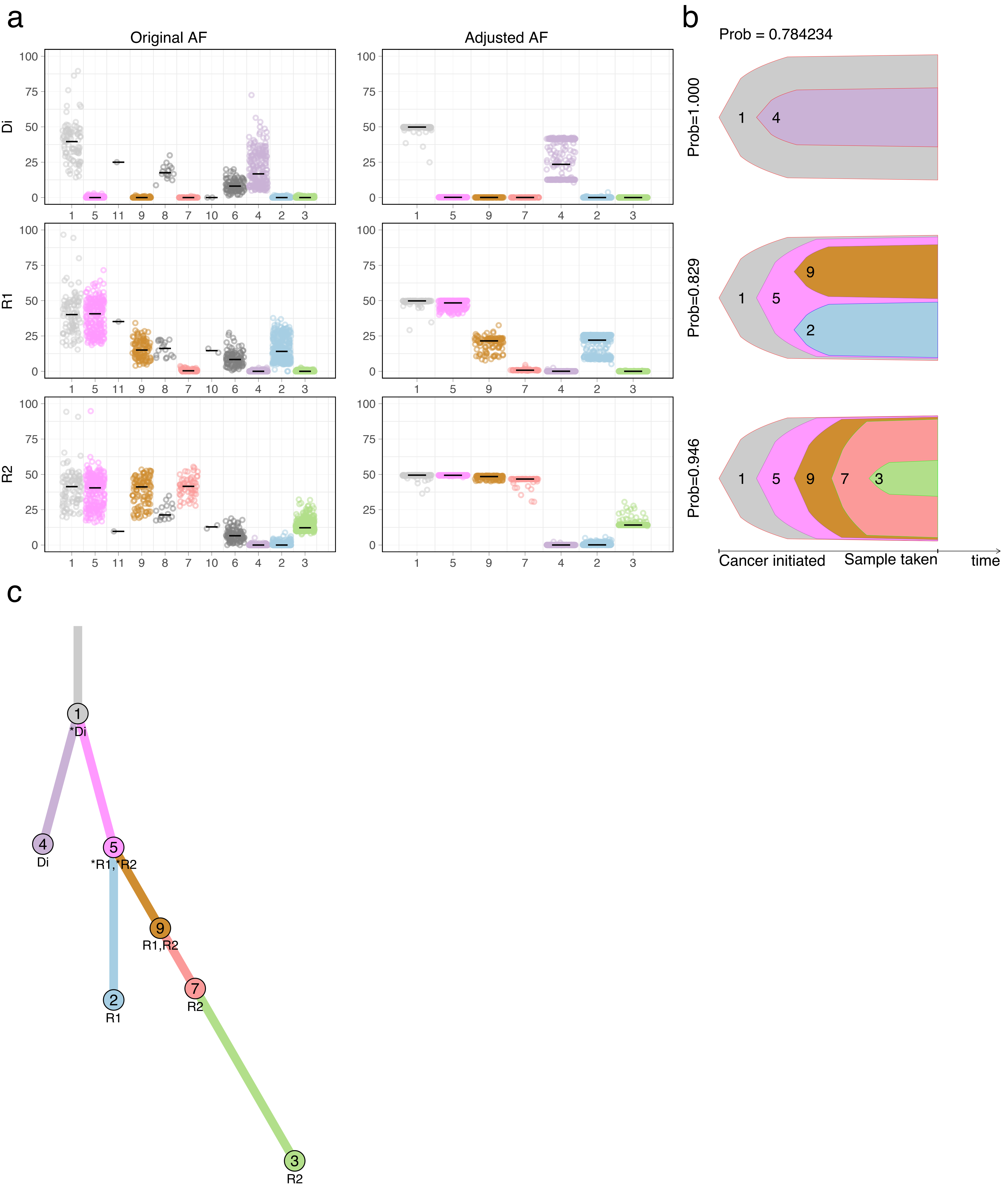

ALL_835 Rising clone

Excluding clusters: 6, 10, 8, 9
Model: monoclonal

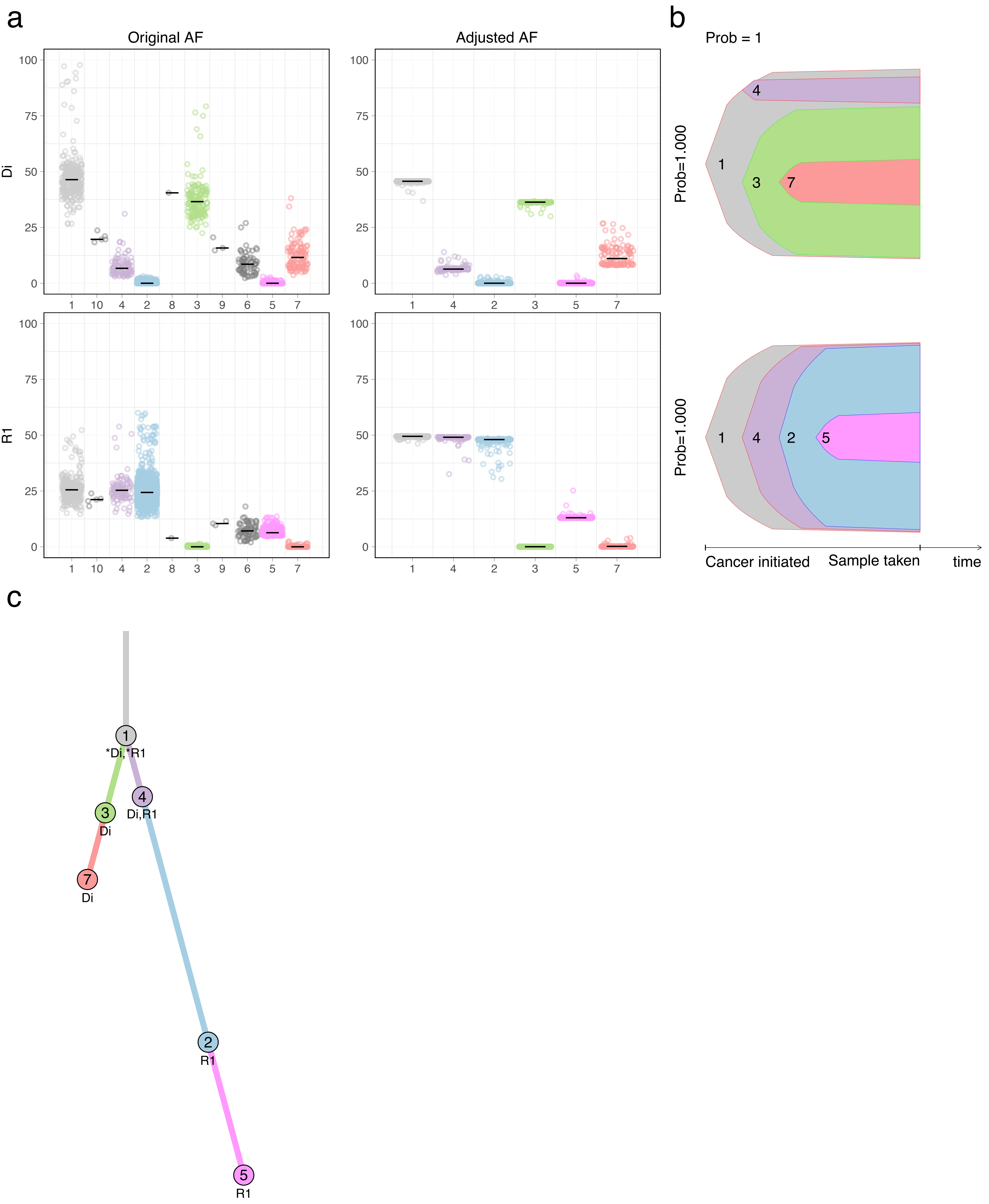

ALL_5 Rising clone

Excluding clusters: 10, 9, 7
Adjusting cluster medians: R1 6:−2
Model: monoclonal

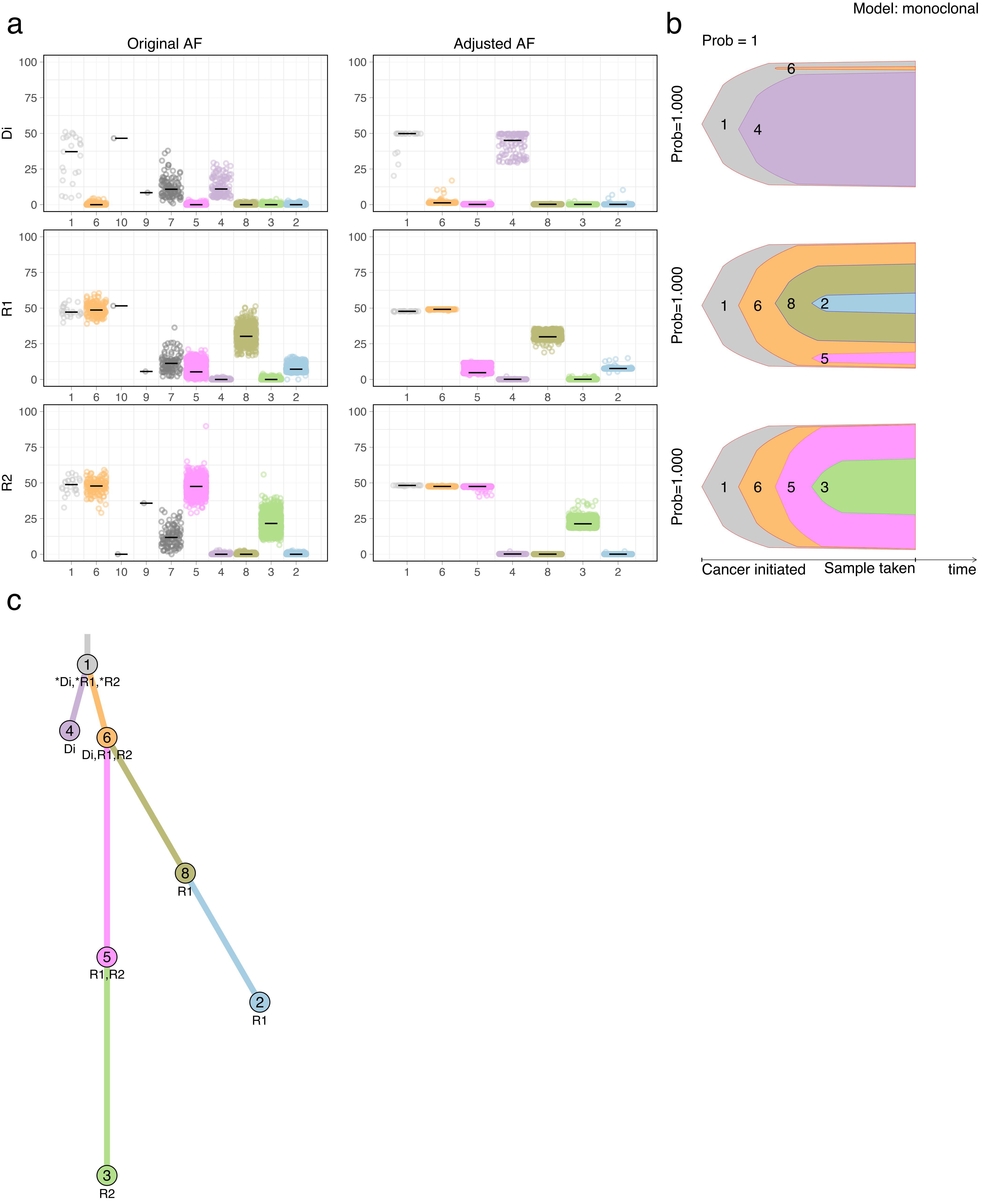

ALL_8 Rising clone

Excluding clusters: 10, 7, 4, 6
Adjusting cluster medians: R1 2:-3
Model: monoclonal

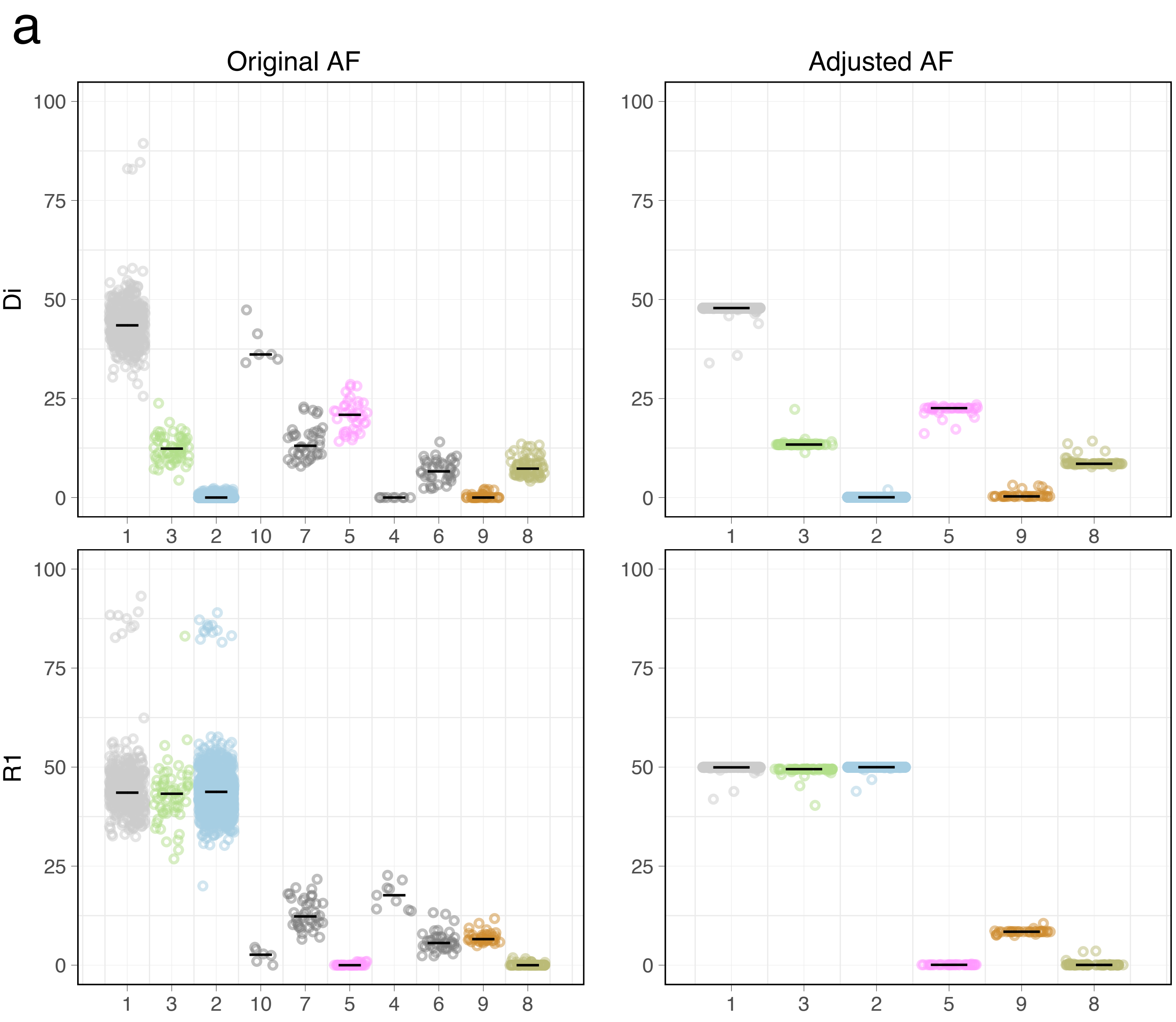


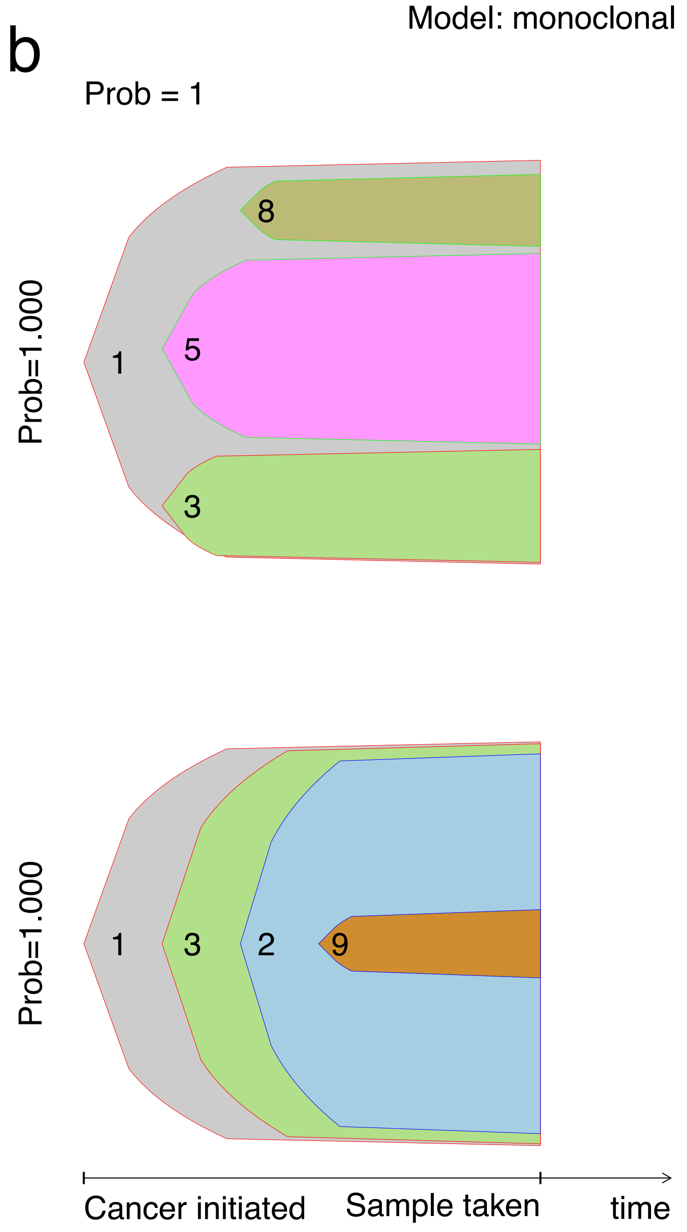


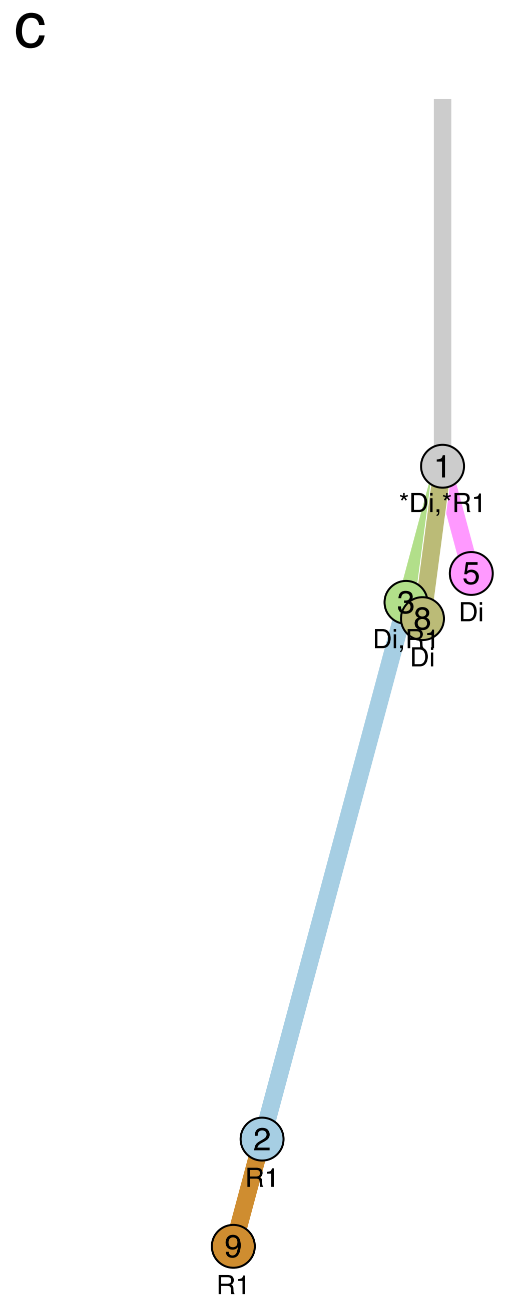

ALL_836 Founding clone

Excluding clusters: 10, 4, 7

Adjusting cluster medians: R1 5:−0.5

Model: monoclonal

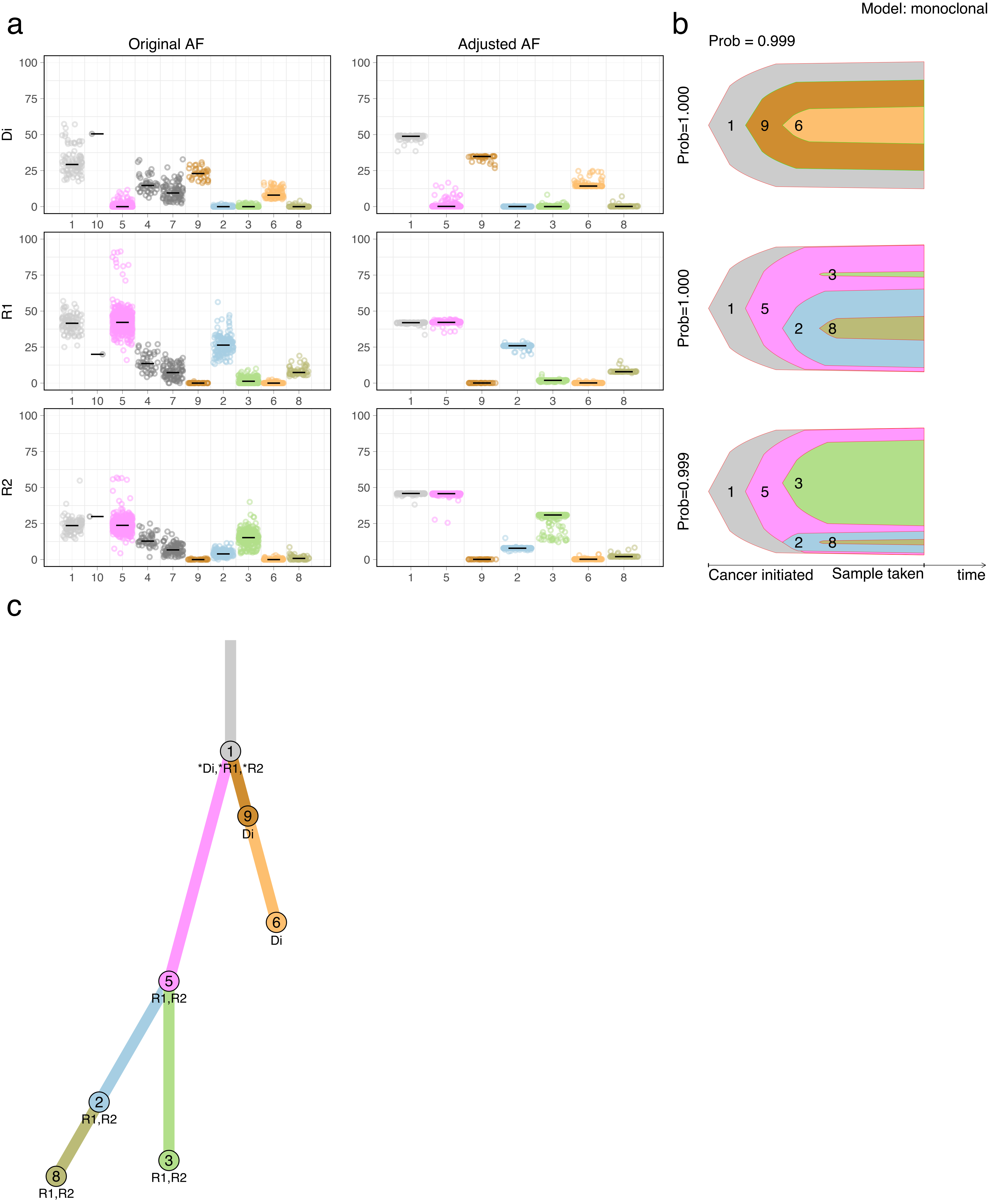

ALL_24 Founding clone

Excluding clusters: 6, 10, 8, 4, 7, 9
Adjusting cluster medians: R1 2:−1.2
Model: monoclonal

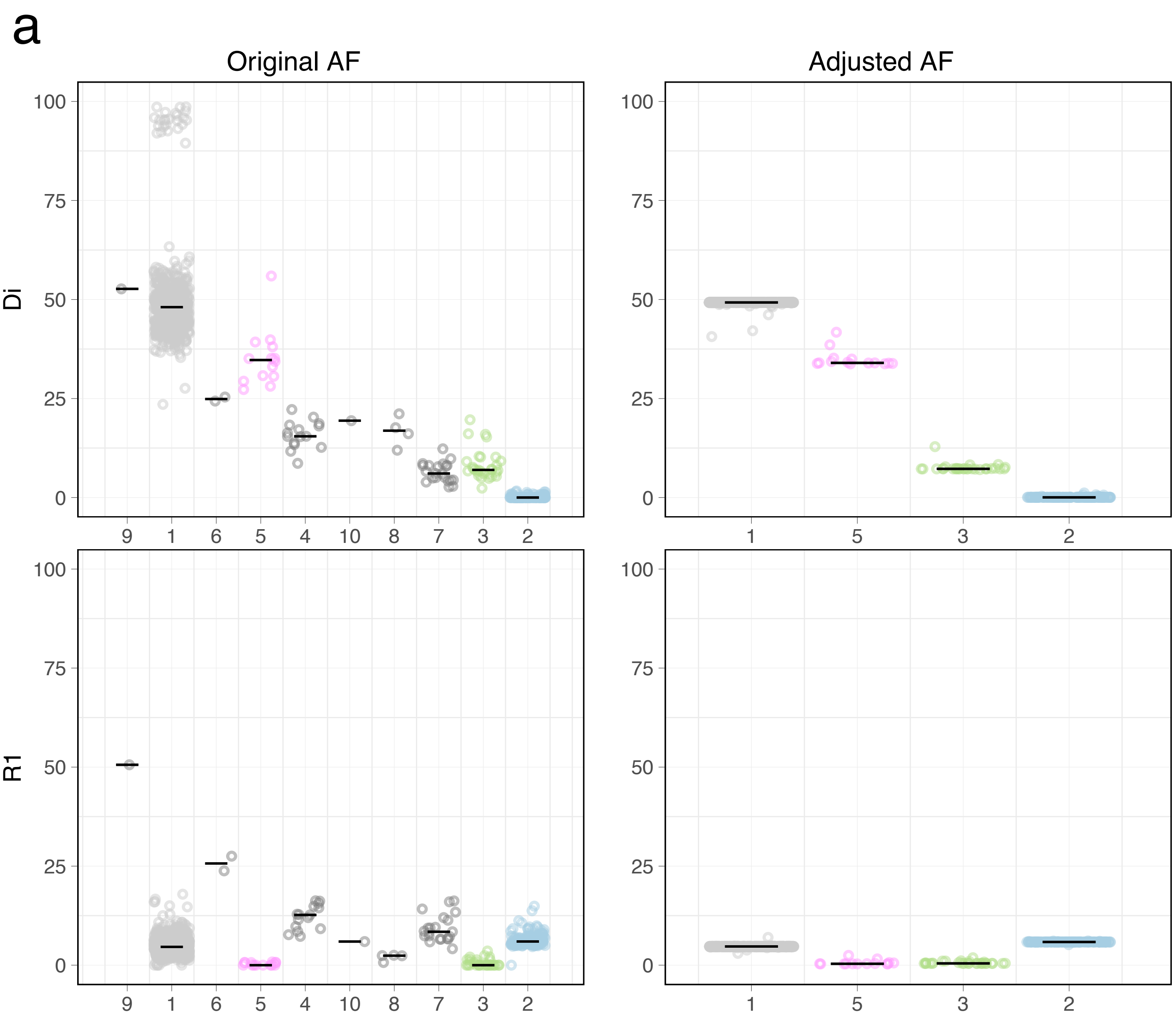


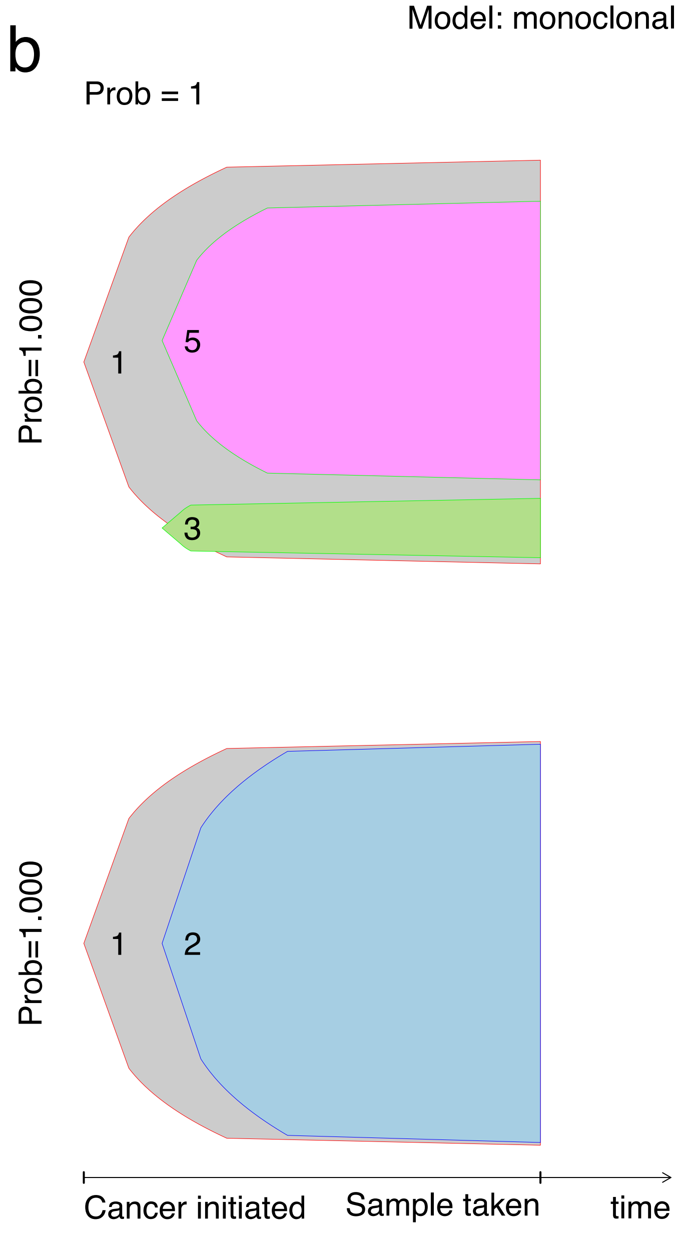


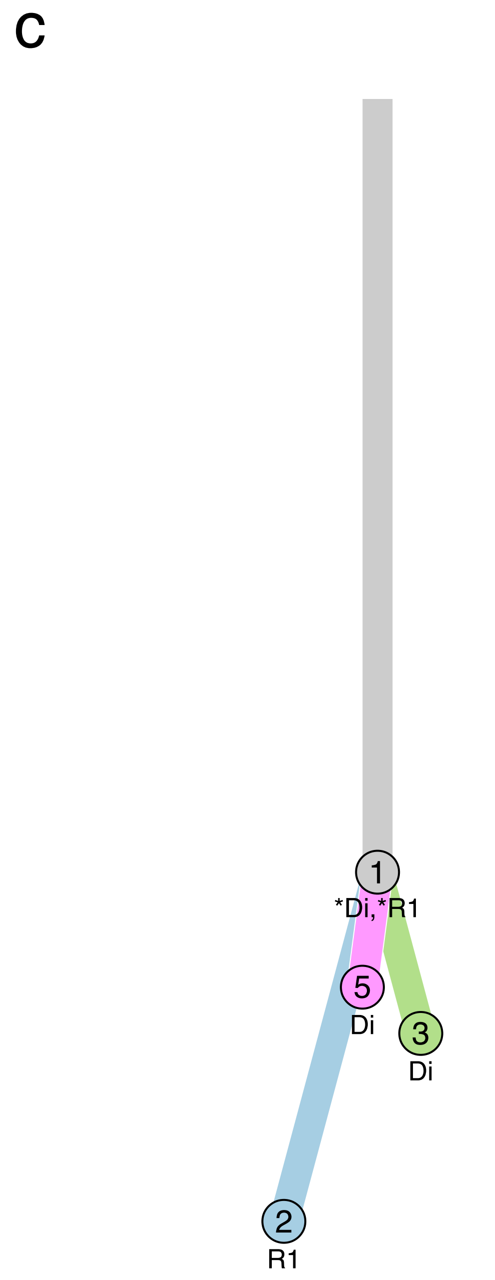

ALL_837 Founding clone

Excluding clusters: 7, 9, 10, 6
Model: monoclonal

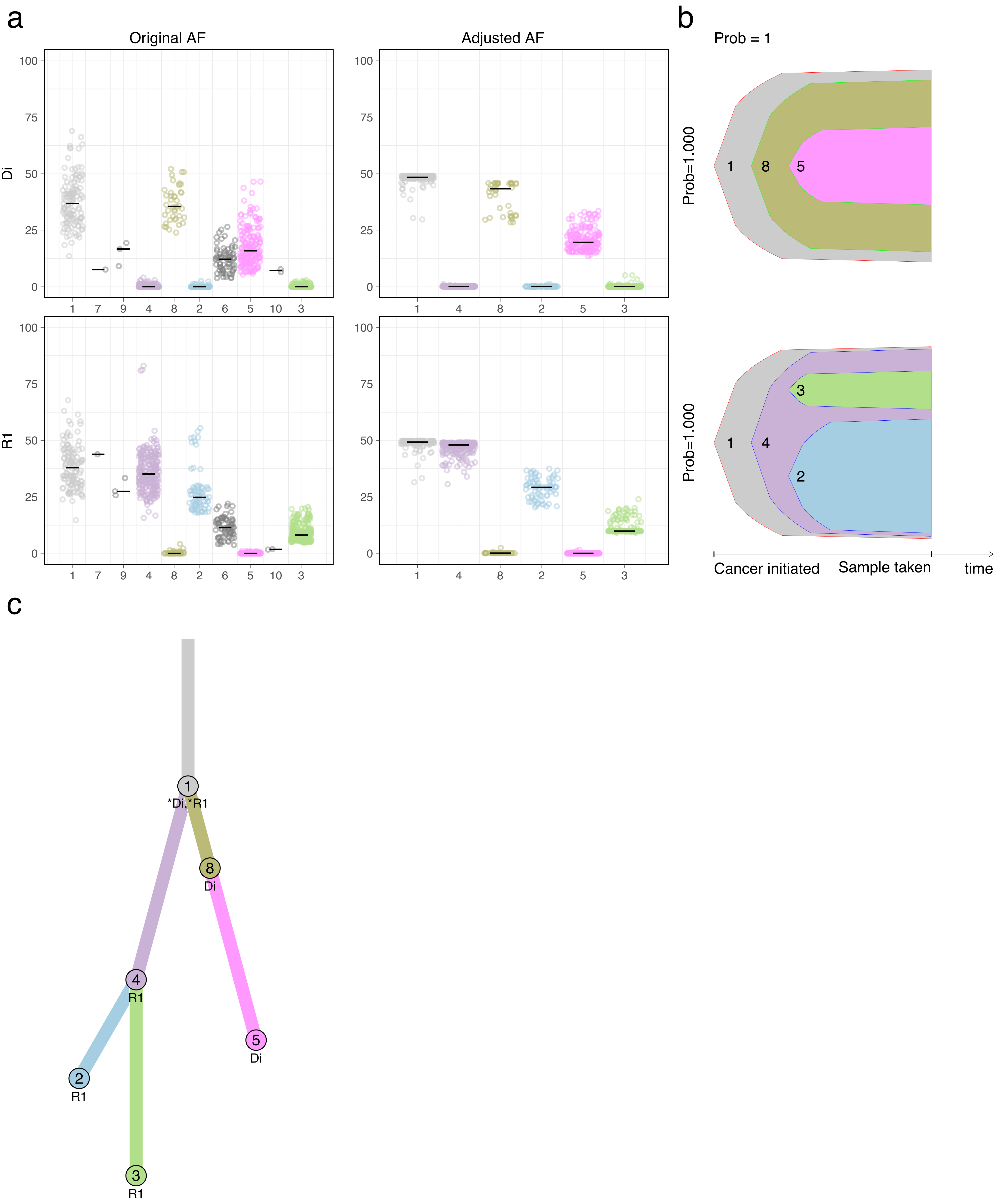

ALL_27 Founding clone

Excluding clusters: 9, 10, 2
Adjusting cluster medians: Di 4:−1 7:−1, R1 3:−0.5, R2 3:−0.5
Model: monoclonal

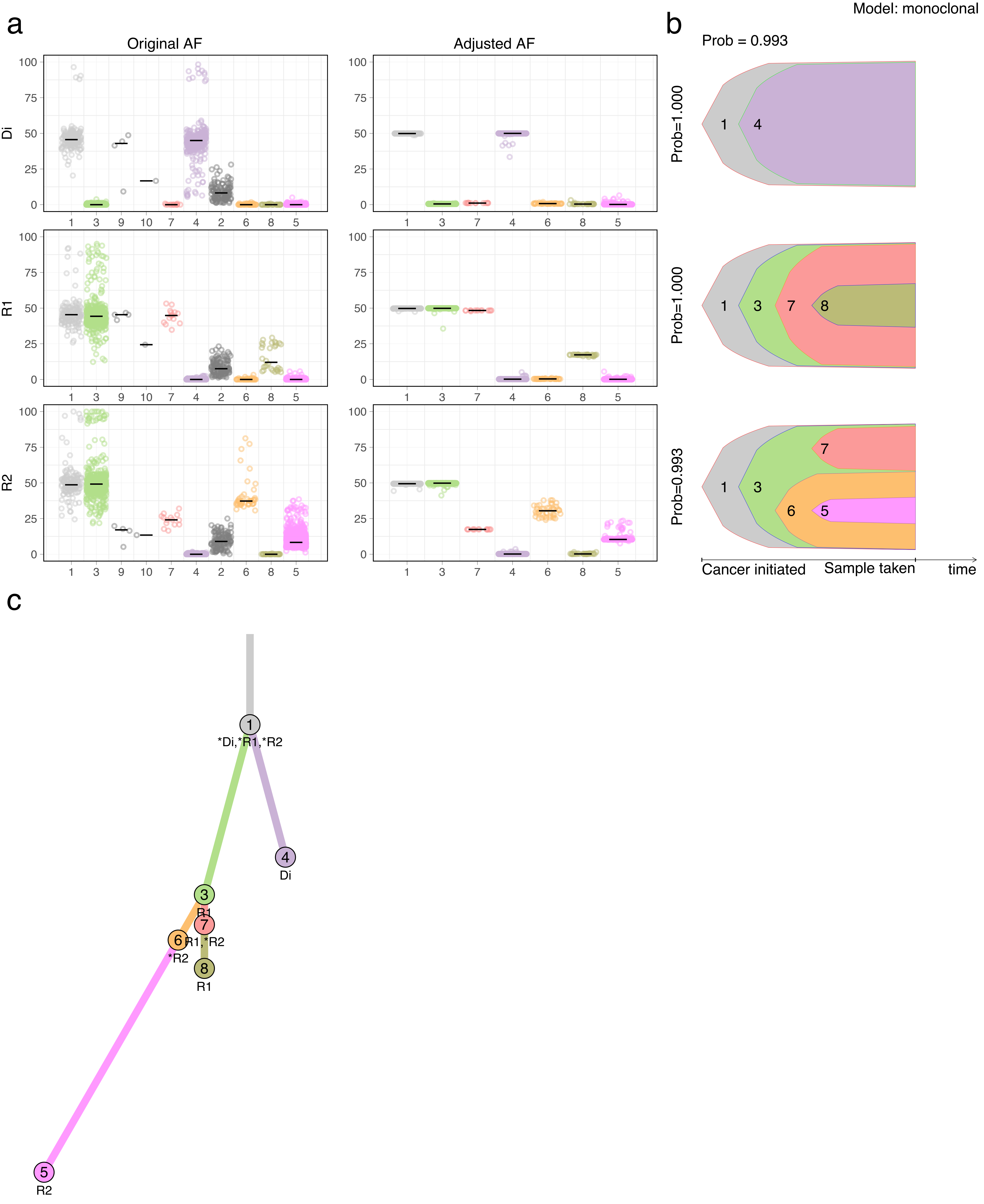

ALL_48 Founding clone

Excluding clusters: 4, 8, 5, 9, 10, 7
Adjusting cluster medians: R1 3:−0.9
Model: monoclonal

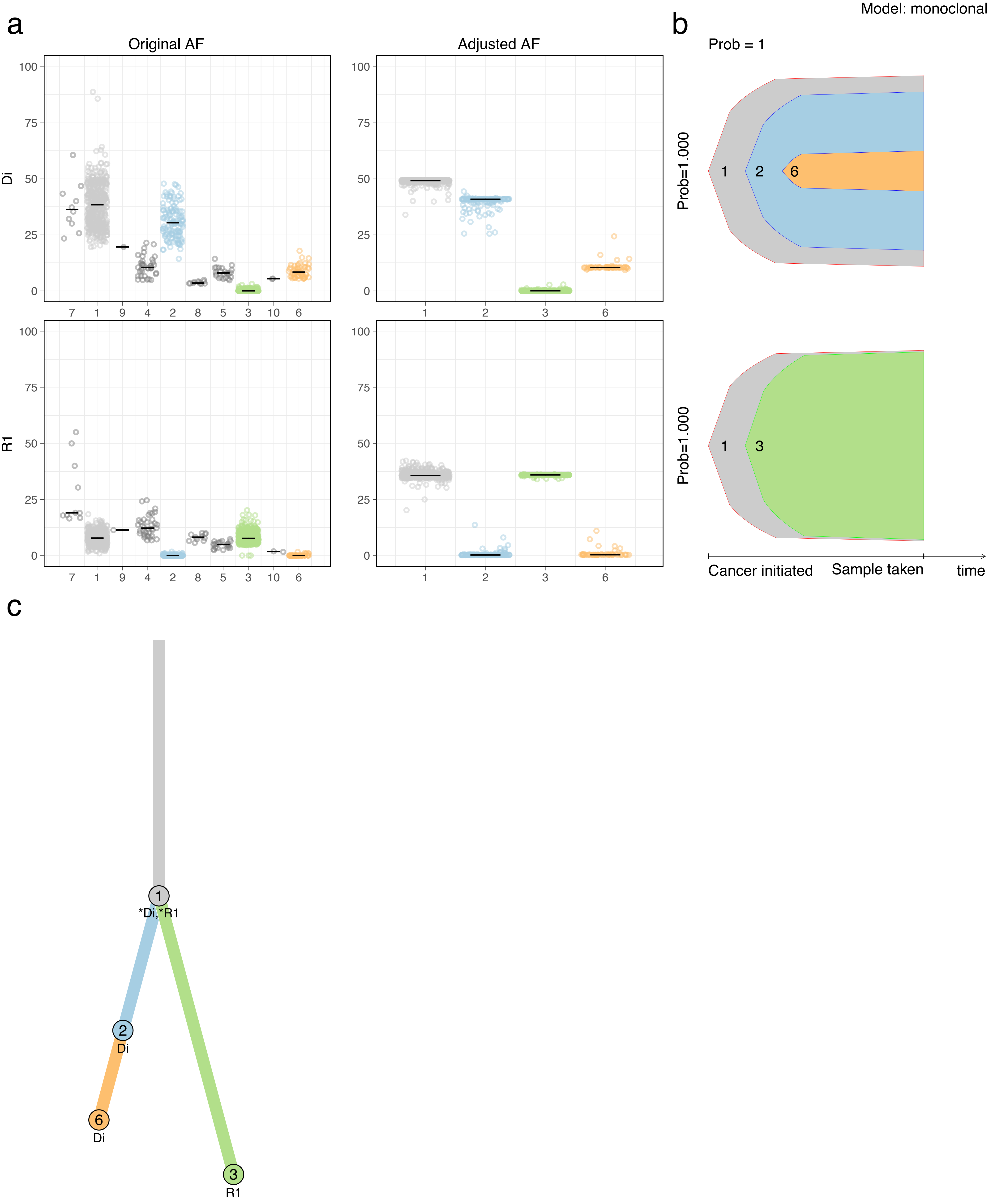

ALL_109 Founding clone

Excluding clusters: 5, 9
Adjusting cluster medians: R2 7:−1
Model: monoclonal

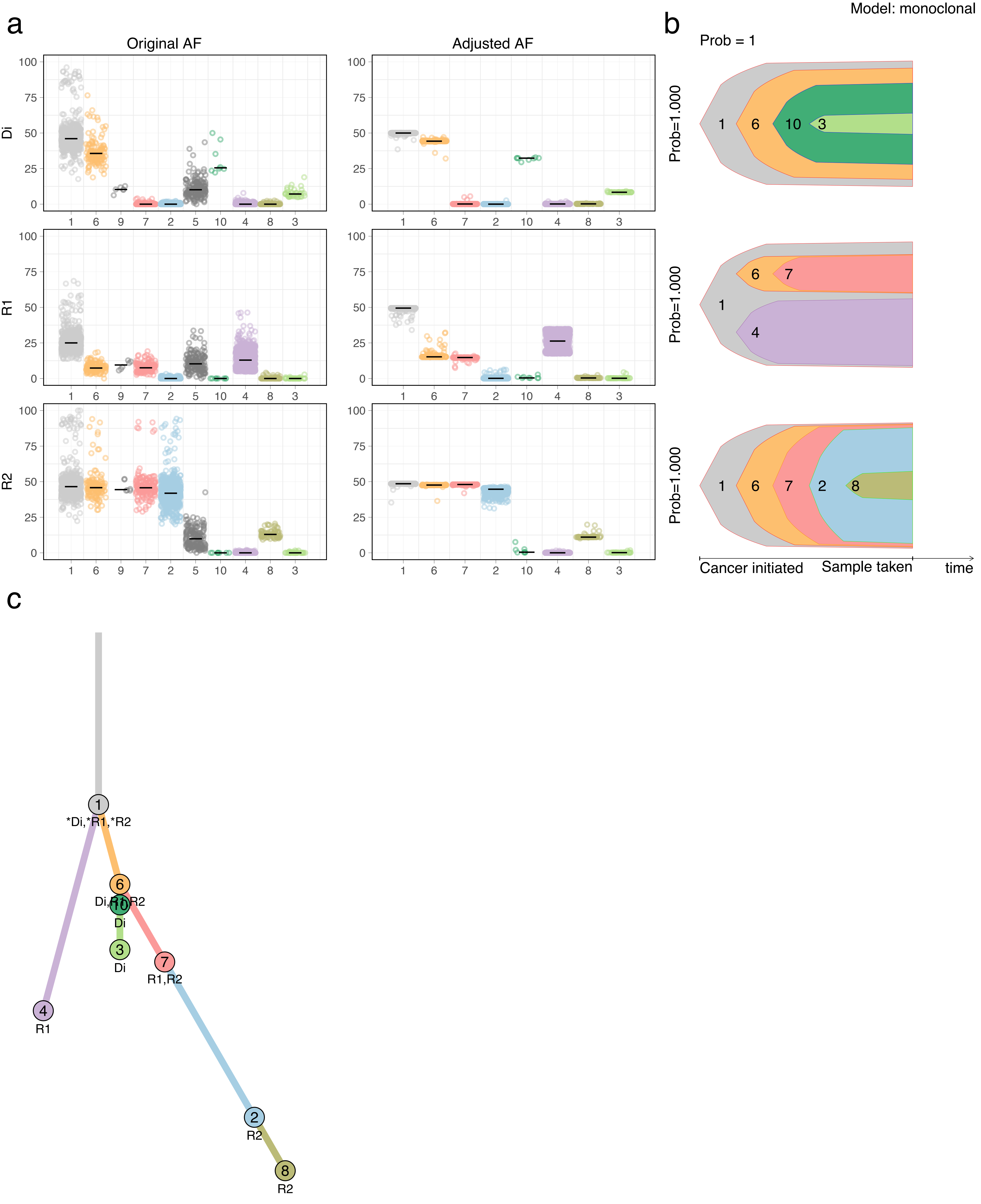

ALL_124 Rising clone

Excluding clusters: 9, 10, 5

Adjusting cluster medians: R1 7:−1, Di 8:−4

Model: monoclonal

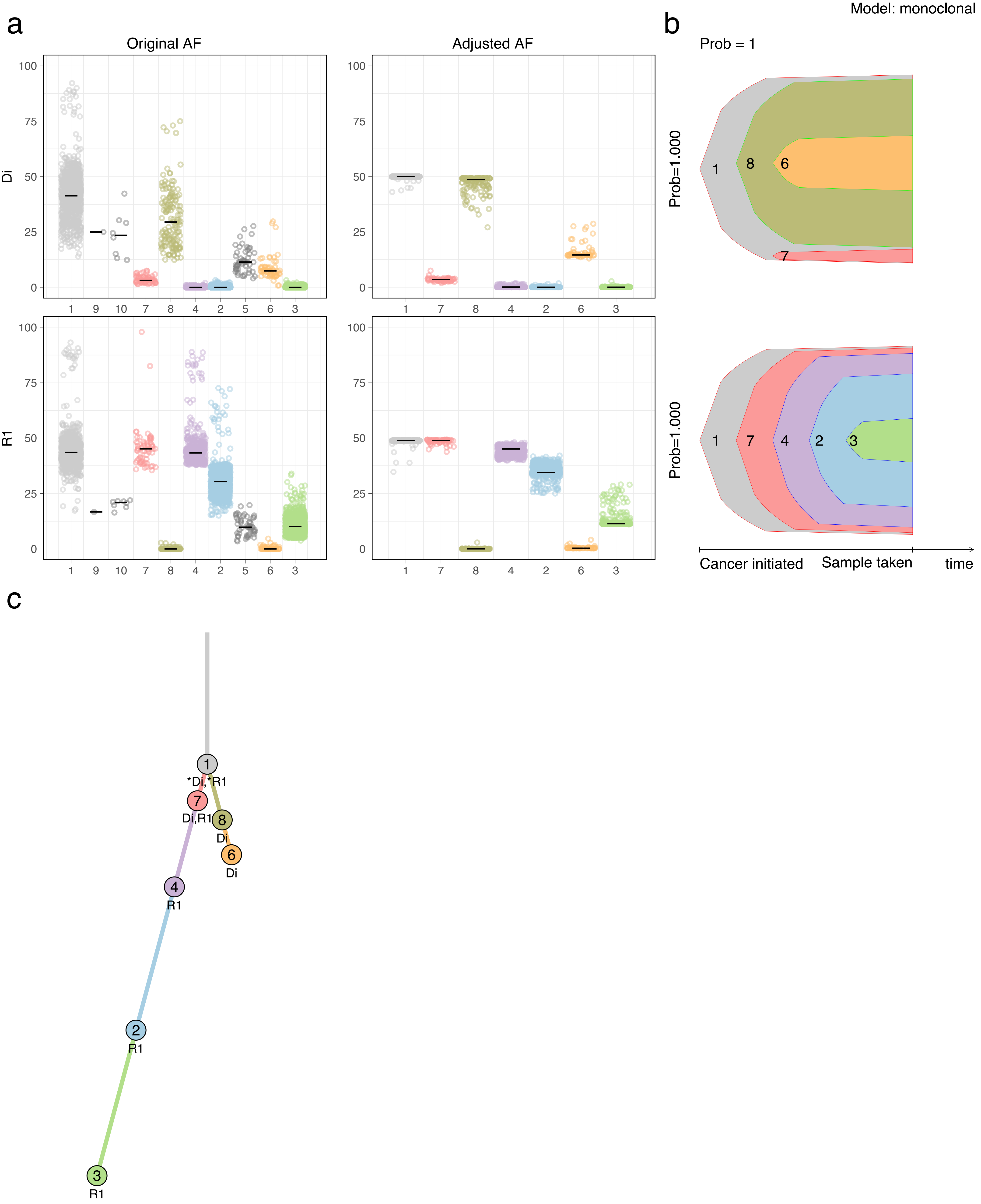

ALL_838 Founding clone

Excluding clusters: 4, 7, 6, 10, 9
Model: monoclonal

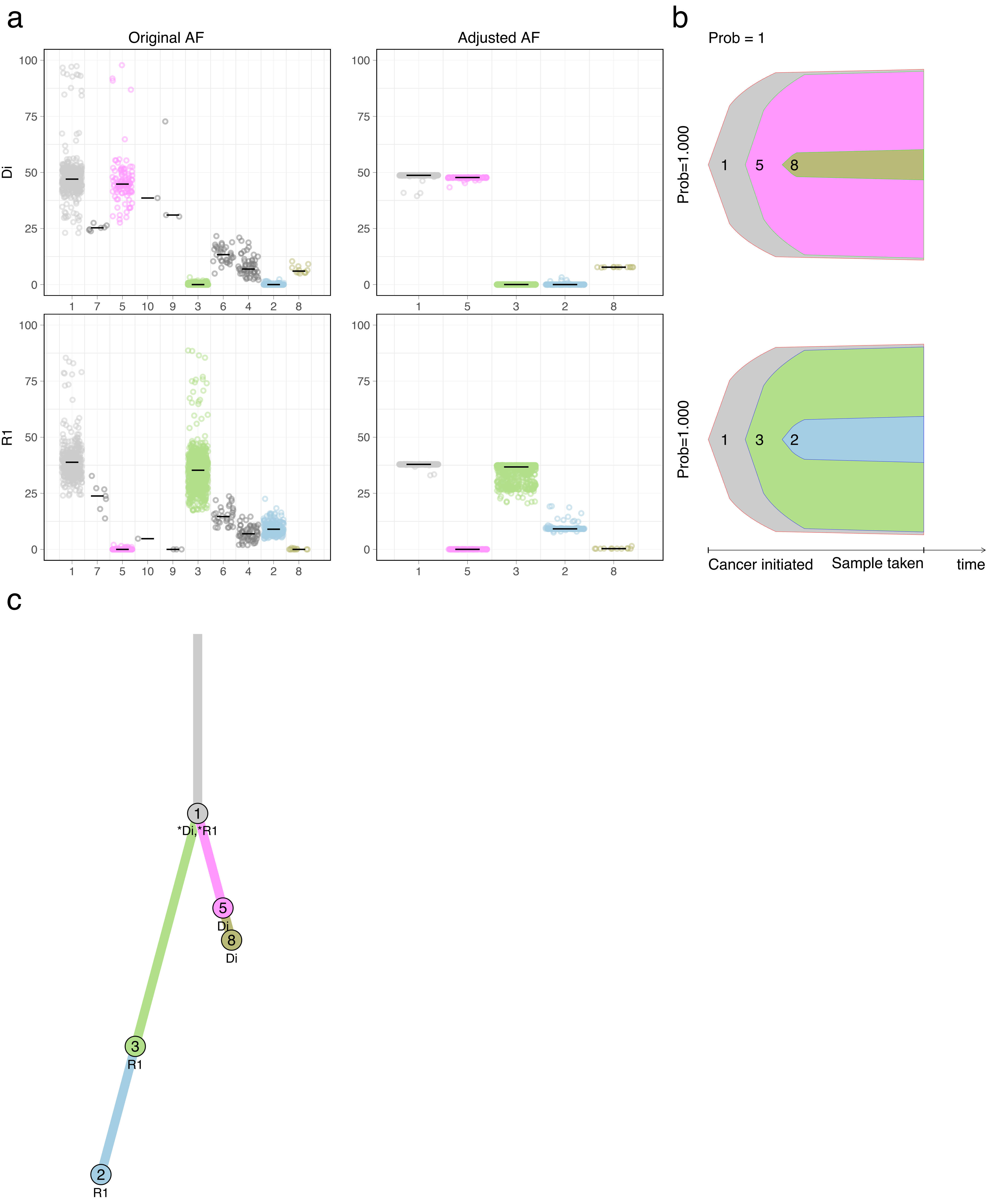

ALL_128 Persistent clone

Excluding clusters: 8, 10, 9, 6
Model: monoclonal

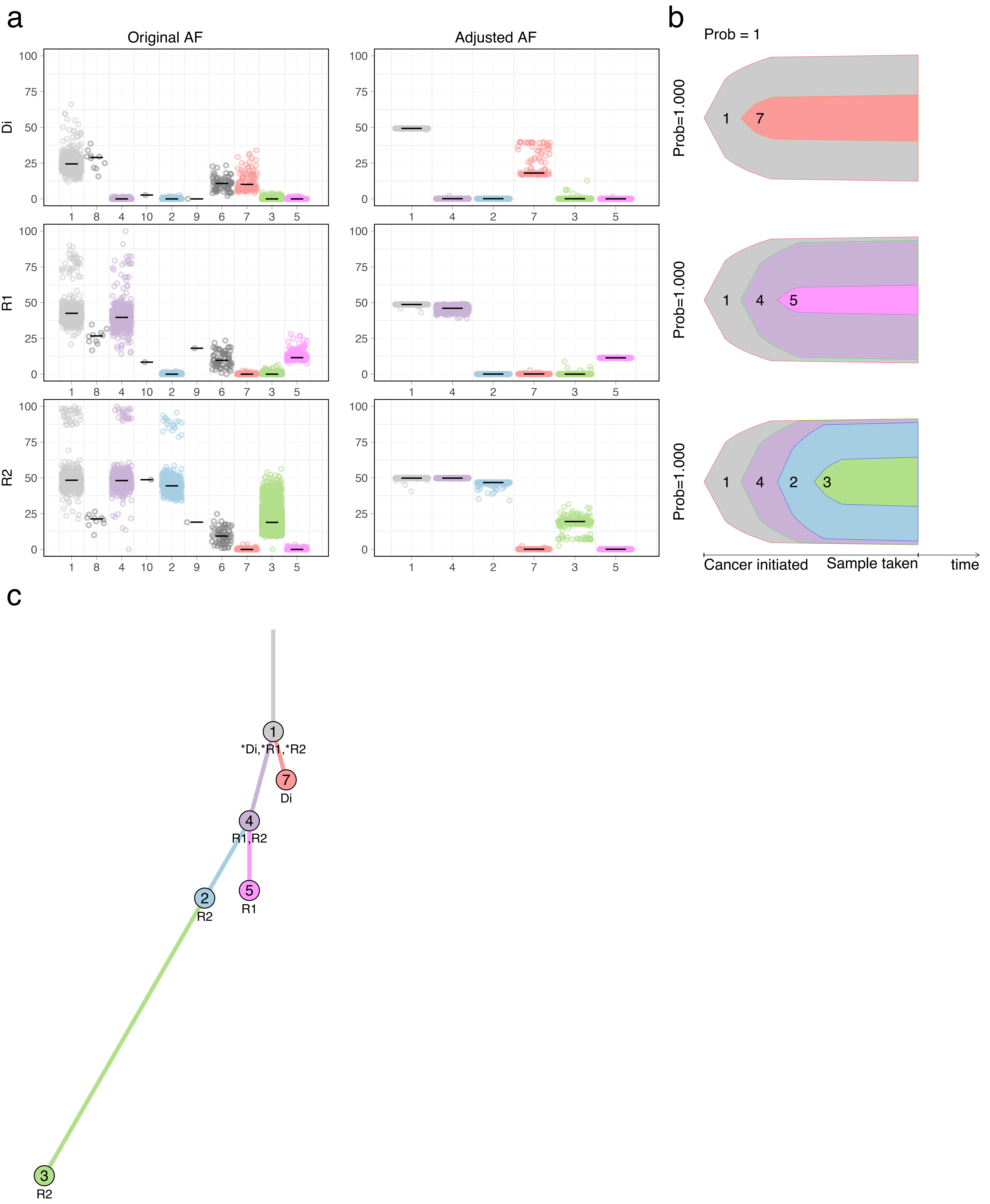

ALL_202 Founding clone

Excluding clusters: 8, 9, 7, 2
Model: monoclonal

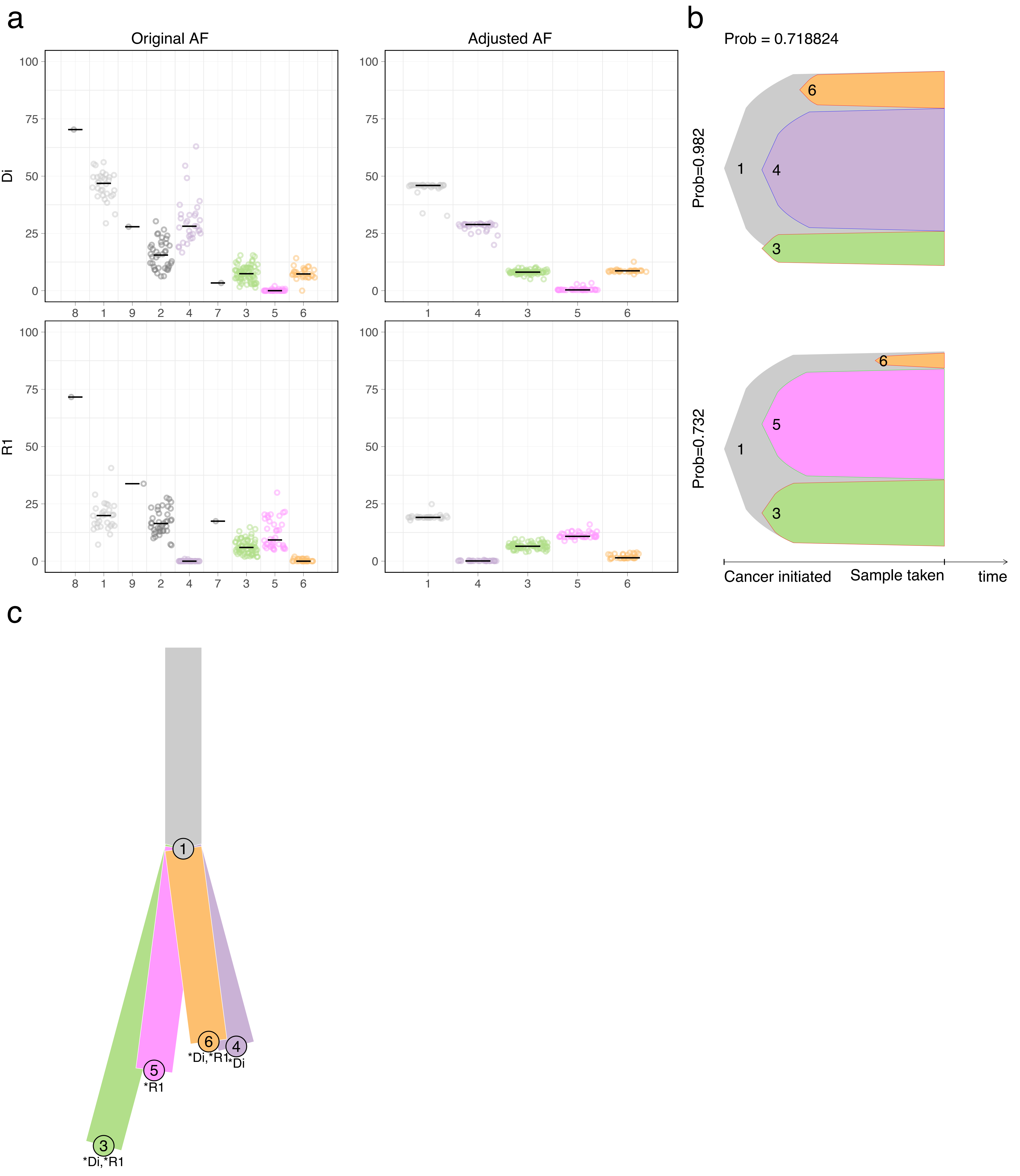

ALL_205 Rising clone

Excluding clusters: 7, 8, 4, 2
Model: monoclonal

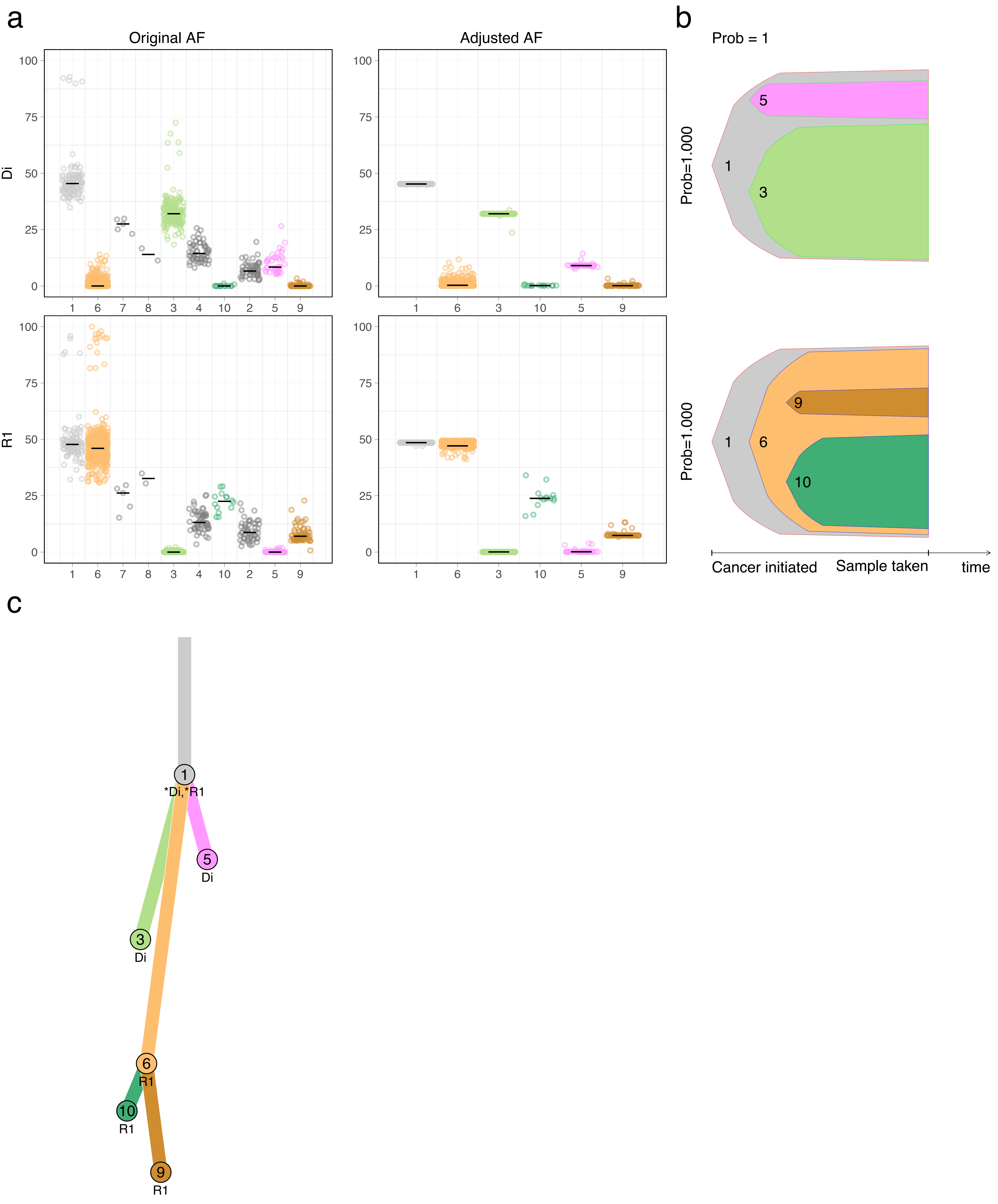

ALL_210 Founding clone

Excluding clusters: 10, 7, 6, 9
Adjusting cluster medians: R1 2:−2
Model: monoclonal

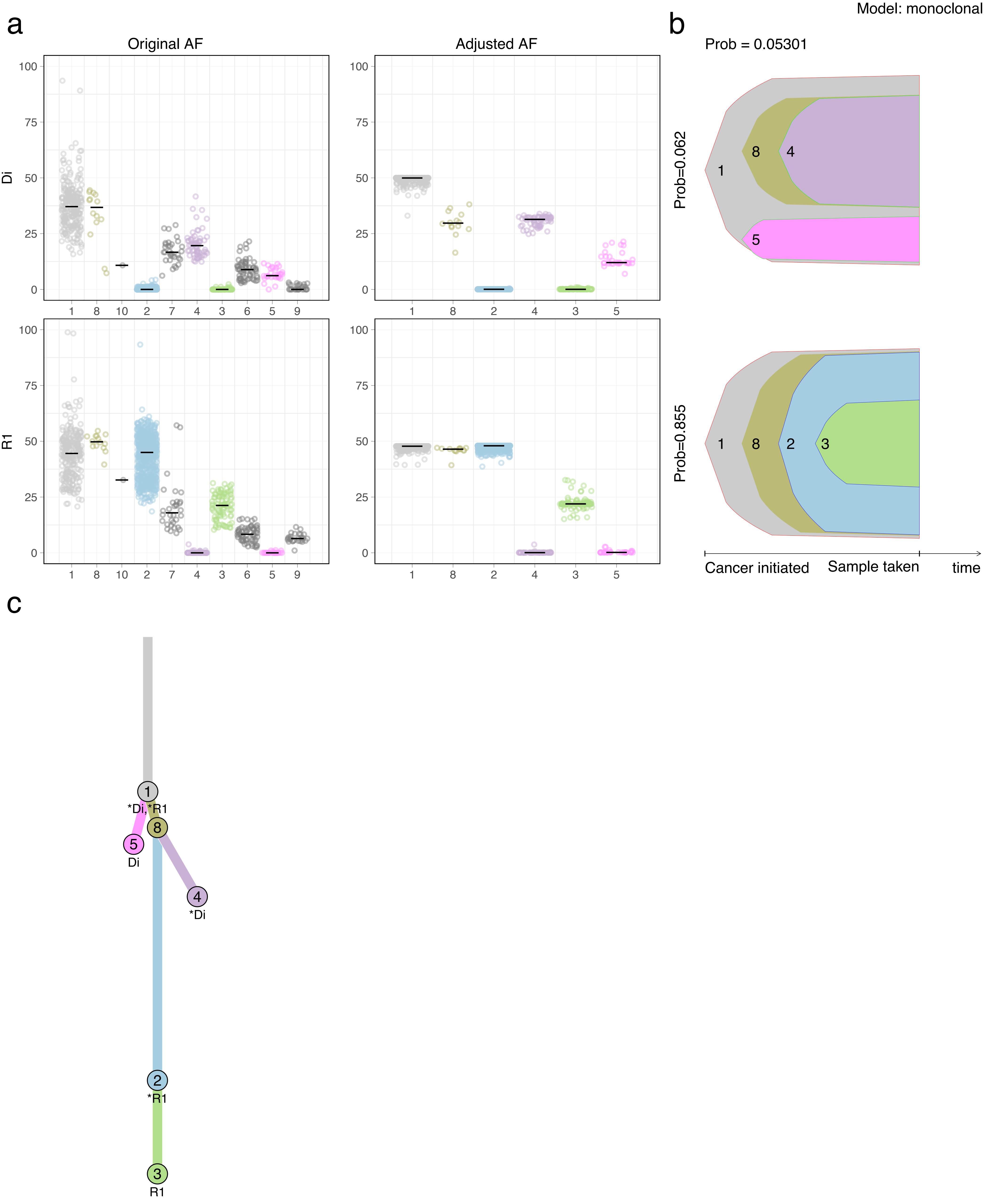

ALL_244 Rising clone

Excluding clusters: 10, 9, 8, 5
Model: monoclonal

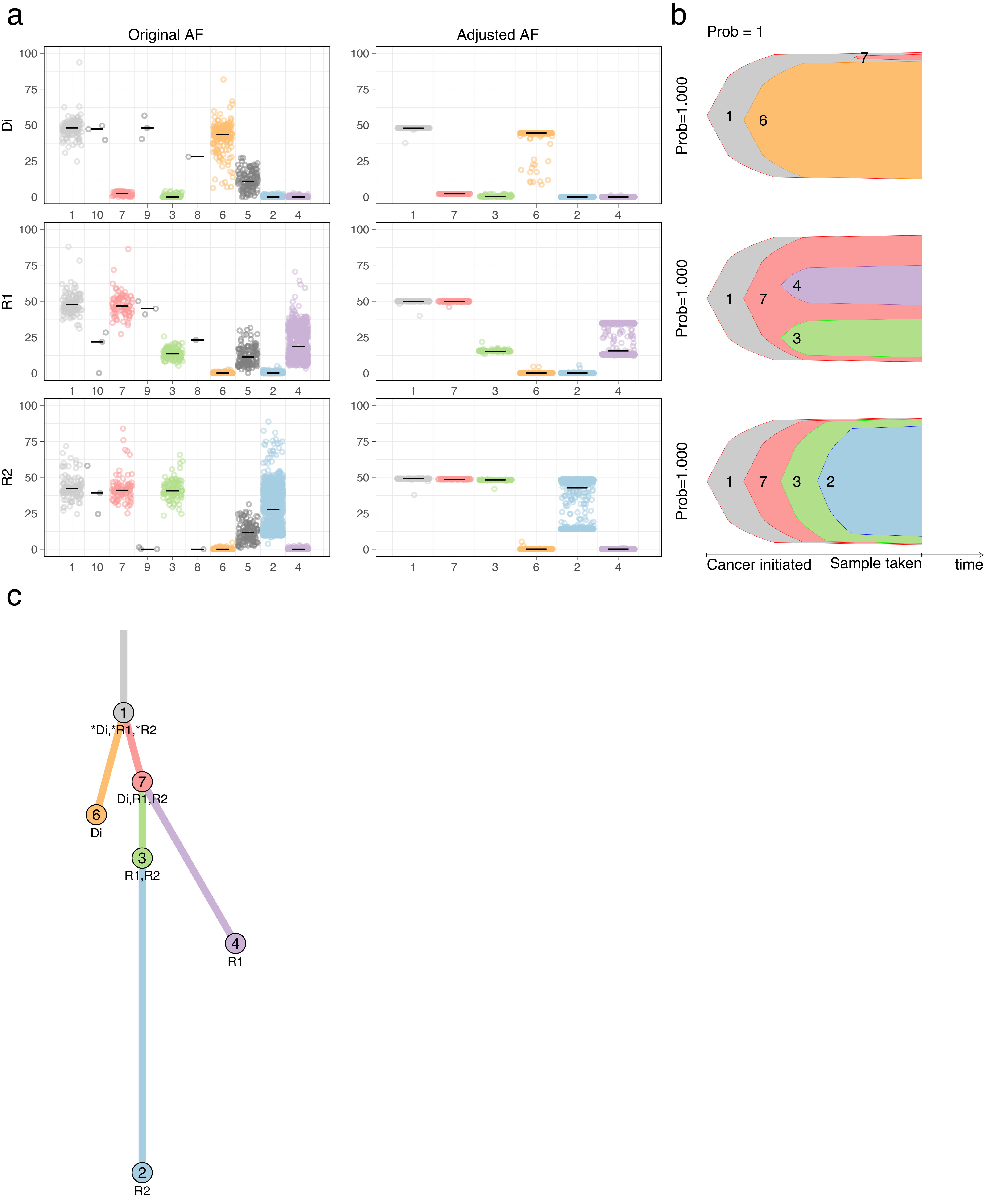

ALL_839 Rising clone

Excluding clusters: 10, 9, 8, 7, 4
Adjusting cluster medians: R1 2:−1.5 5:−1
Model: monoclonal

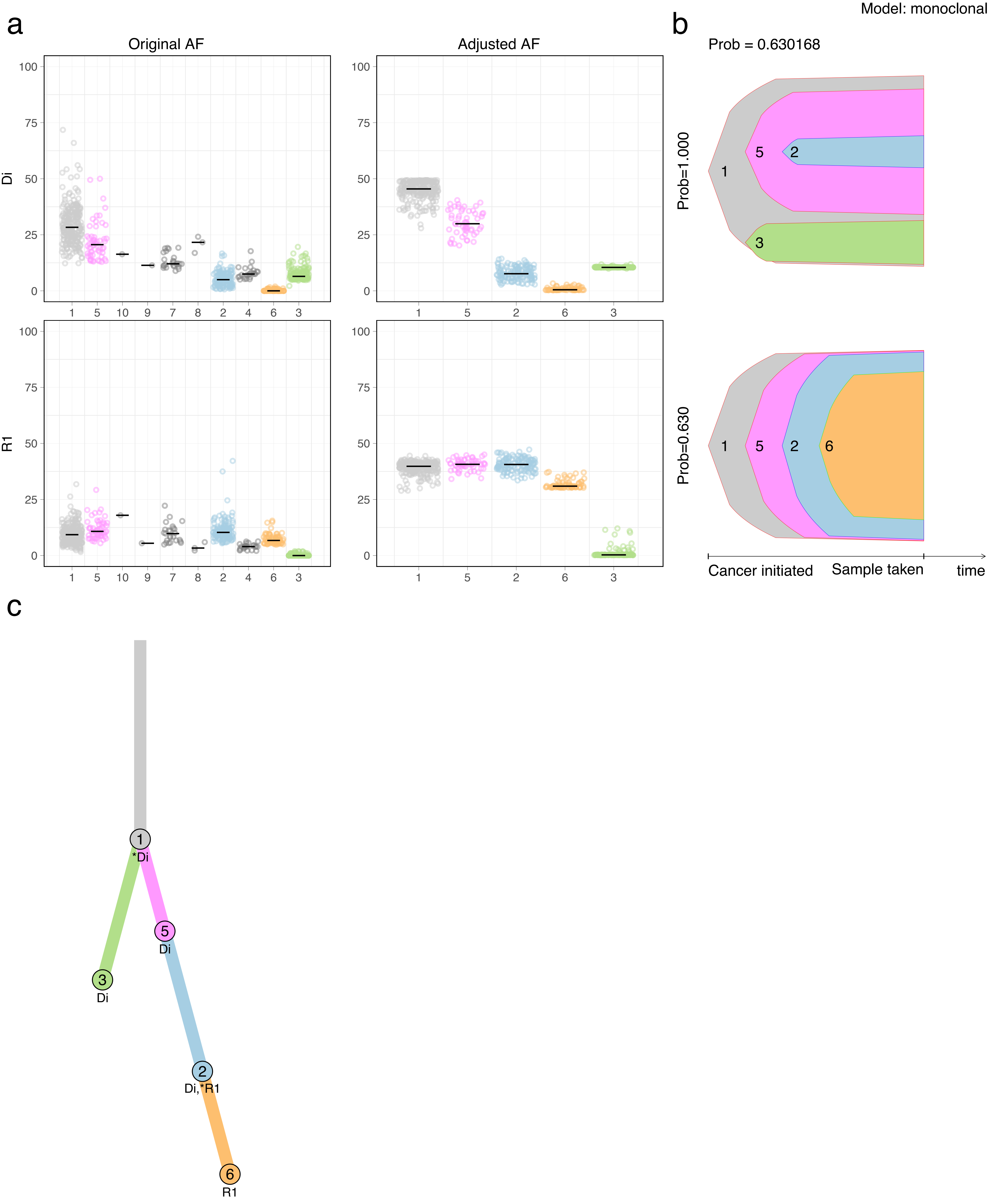

ALL_257 Rising clone

Excluding clusters: 10, 4, 6
Adjusting cluster medians: R1 2:−0.5
Model: monoclonal

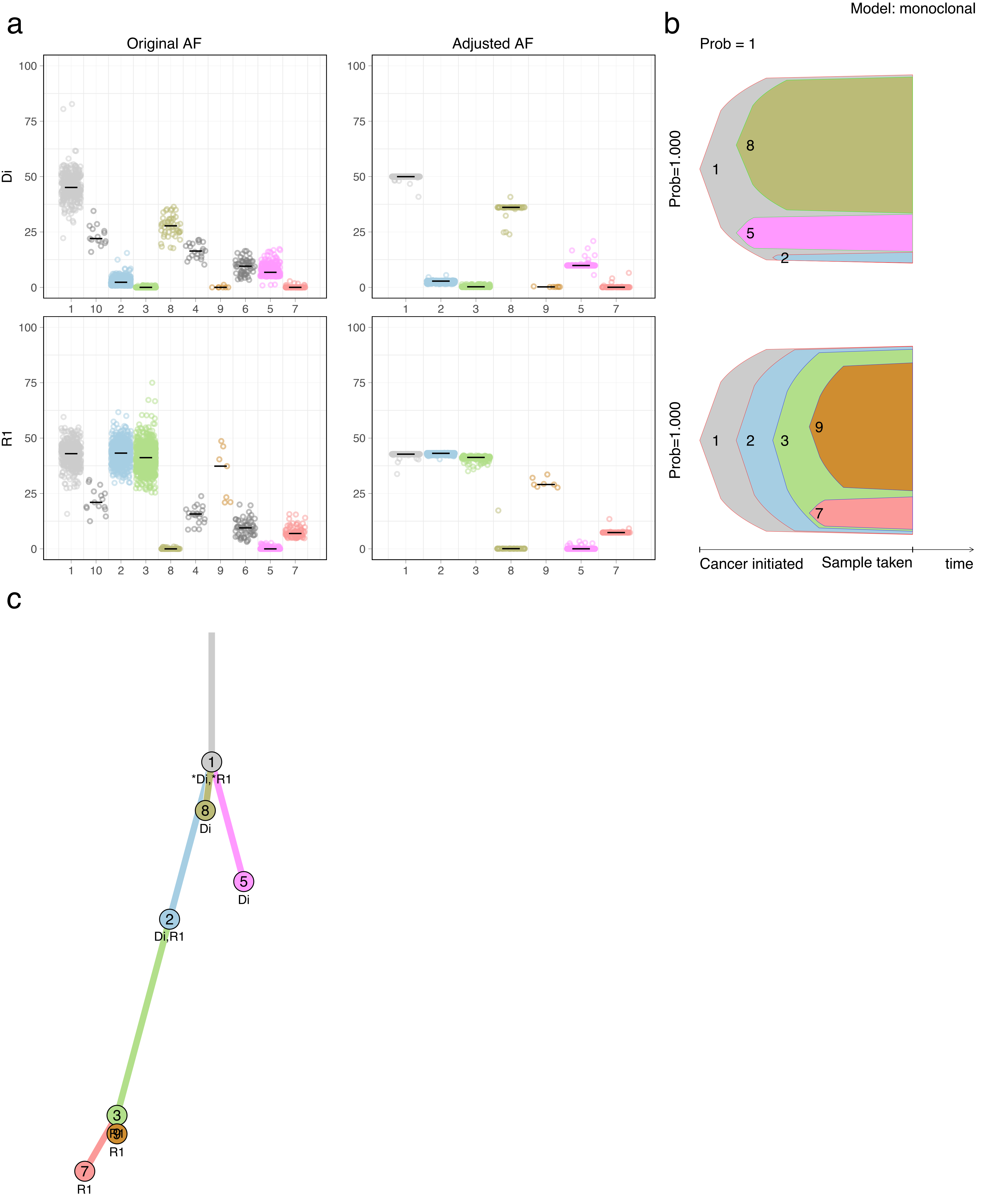

ALL_317 Founding clone

Excluding clusters: 7, 4, 10, 9, 8, 6
Model: monoclonal

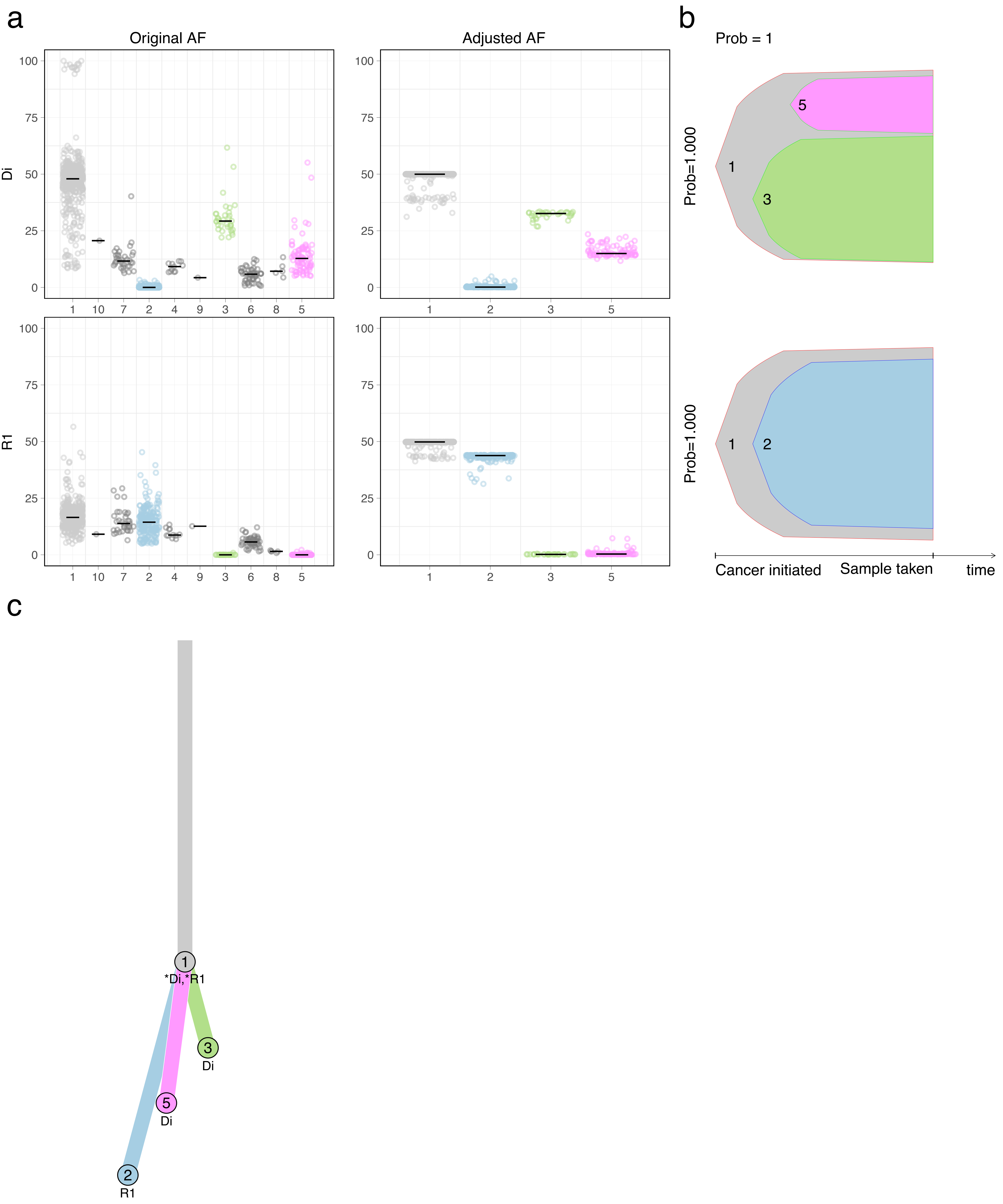

ALL_358 Rising clone

Excluding clusters: 7, 8, 10, 9, 5
Model: monoclonal

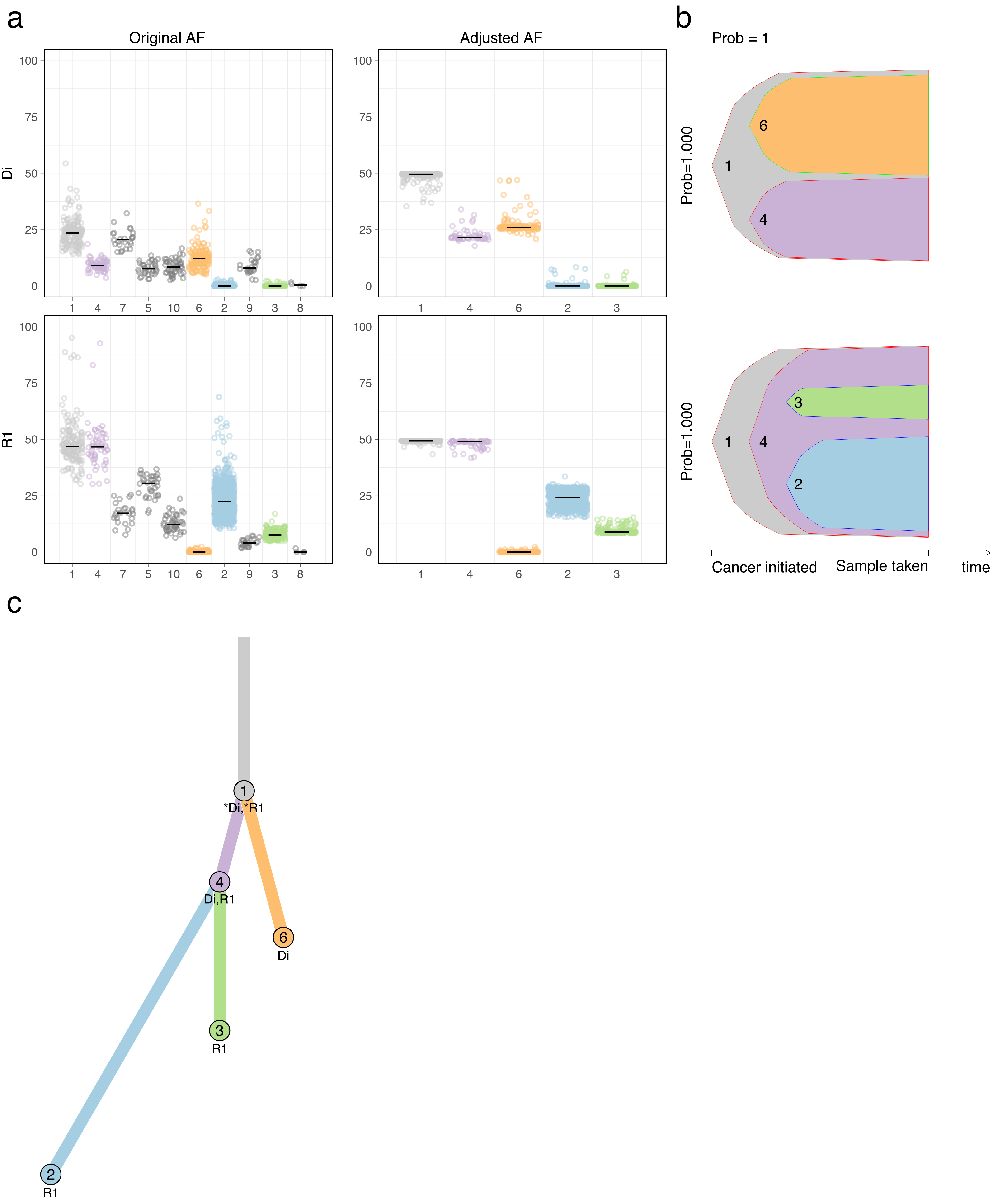

ALL_380 Founding clone

Excluding clusters: 5, 7, 10, 9, 8, 6
Model: monoclonal

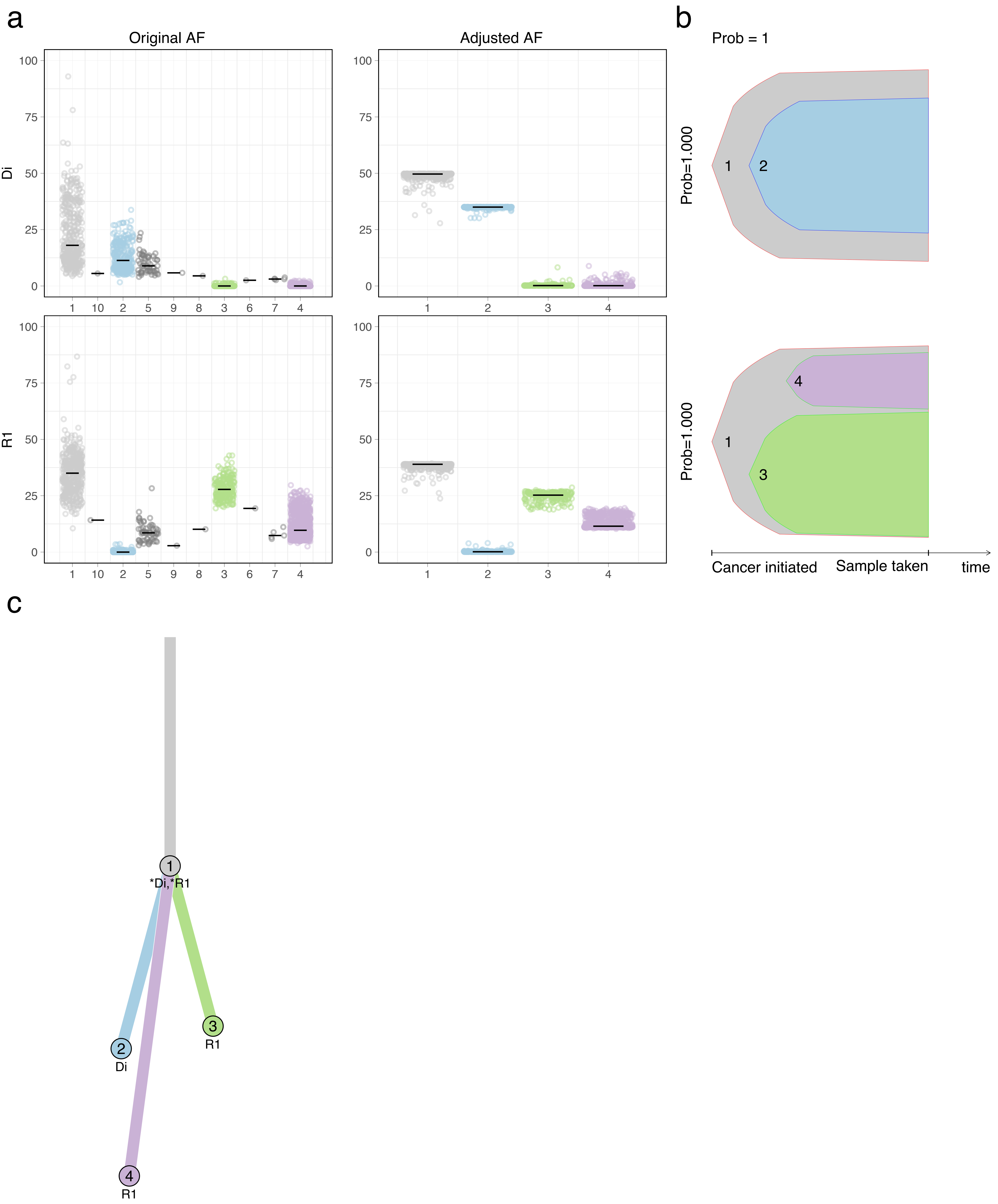

ALL_388 Rising clone

Excluding clusters: 10, 7, 5
Model: monoclonal

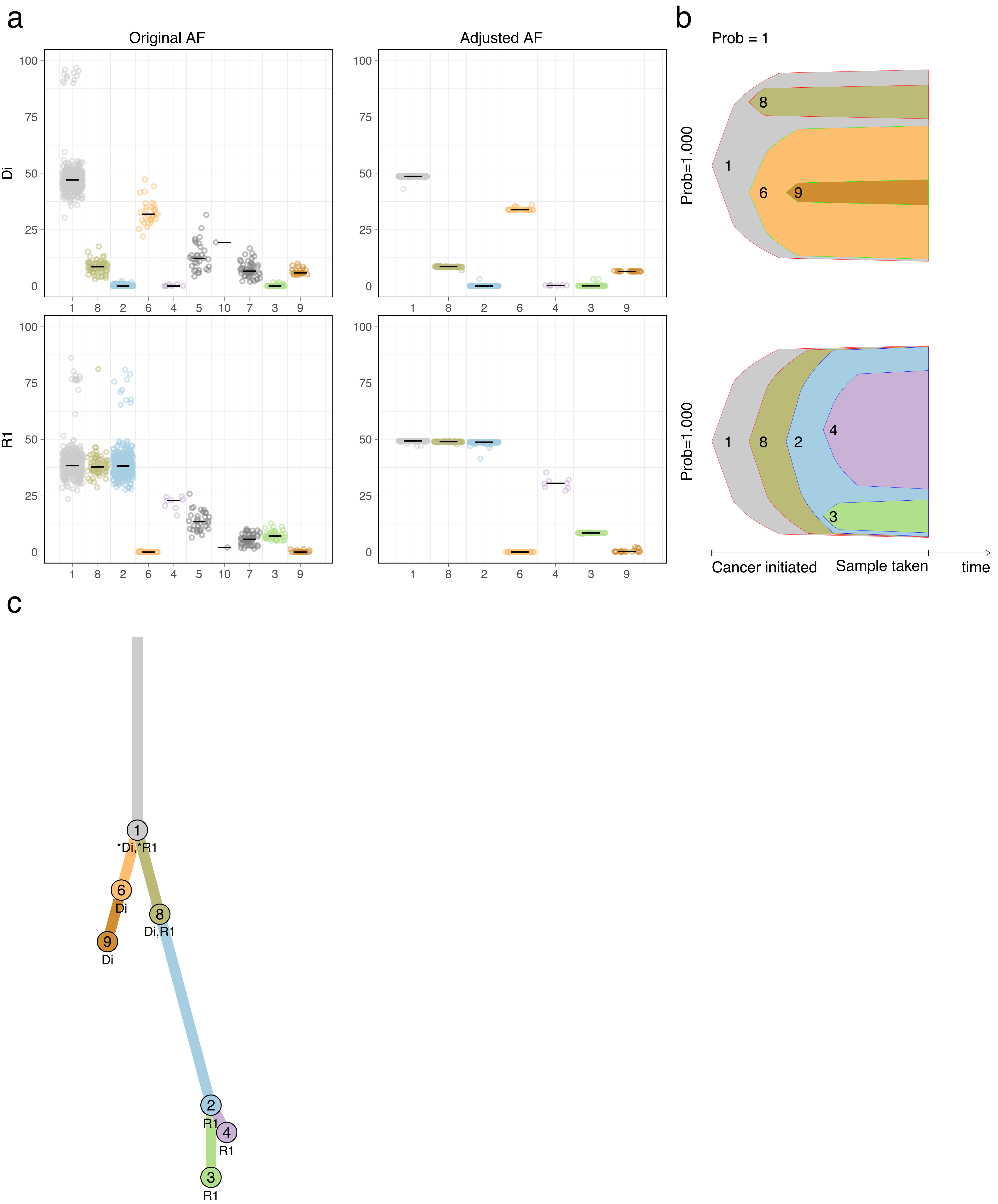

ALL_464 Persistent clone

Excluding clusters: 8, 10, 9
Model: monoclonal

a

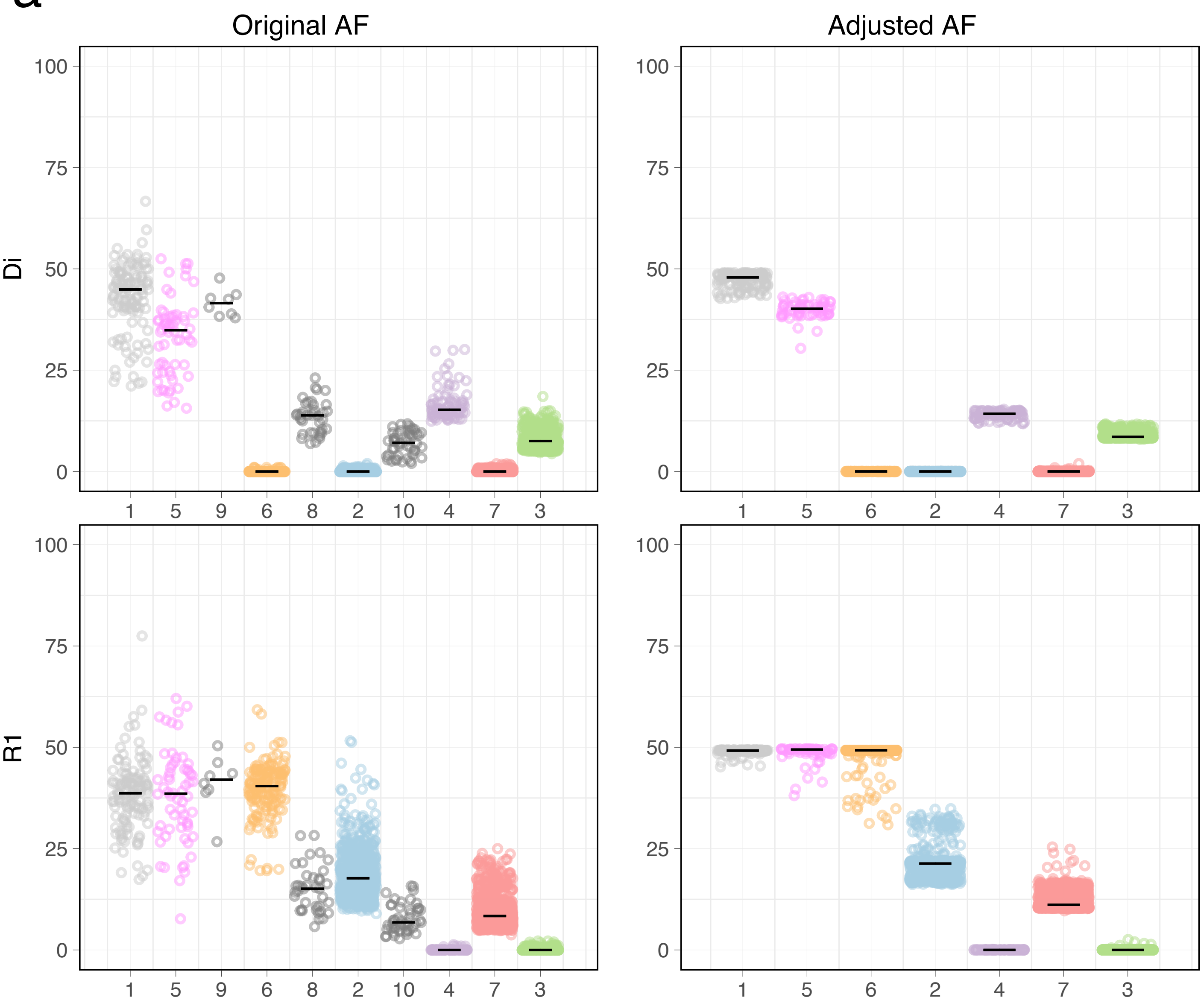


b

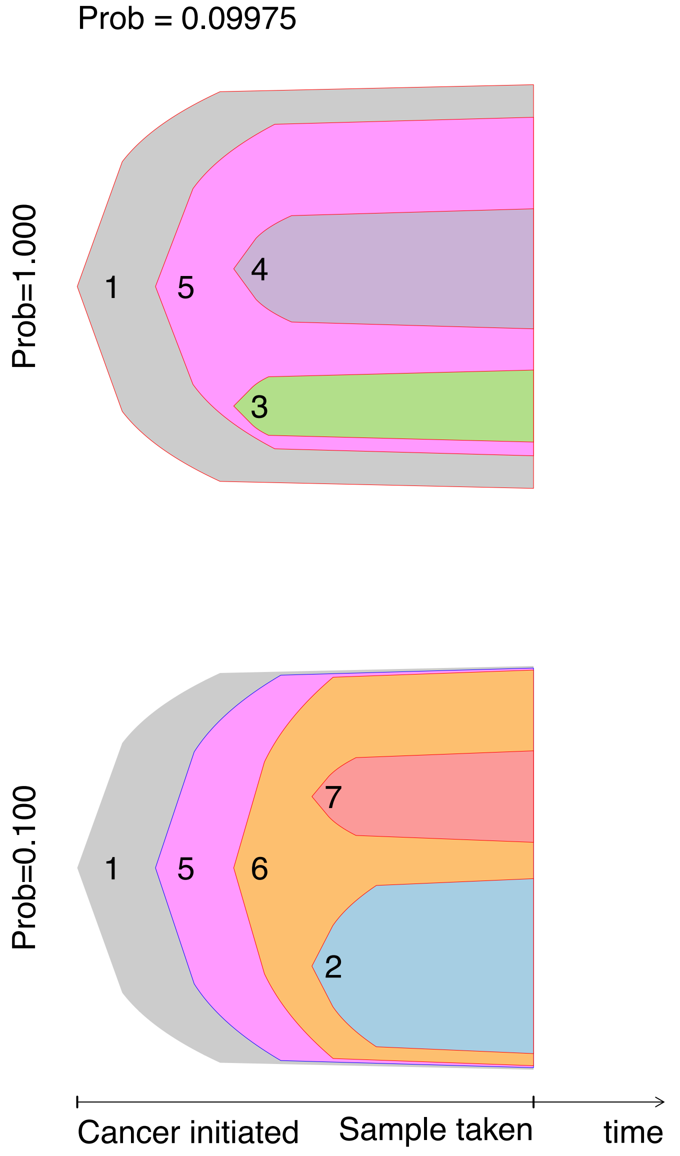


c

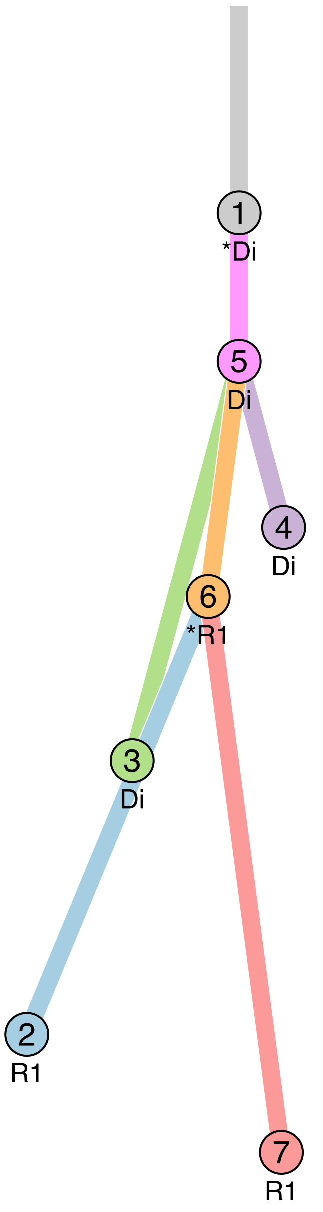

ALL_827 Rising clone

Excluding clusters: 8, 5, 10, 7
Adjusting cluster medians: R1 2:−1
Model: monoclonal

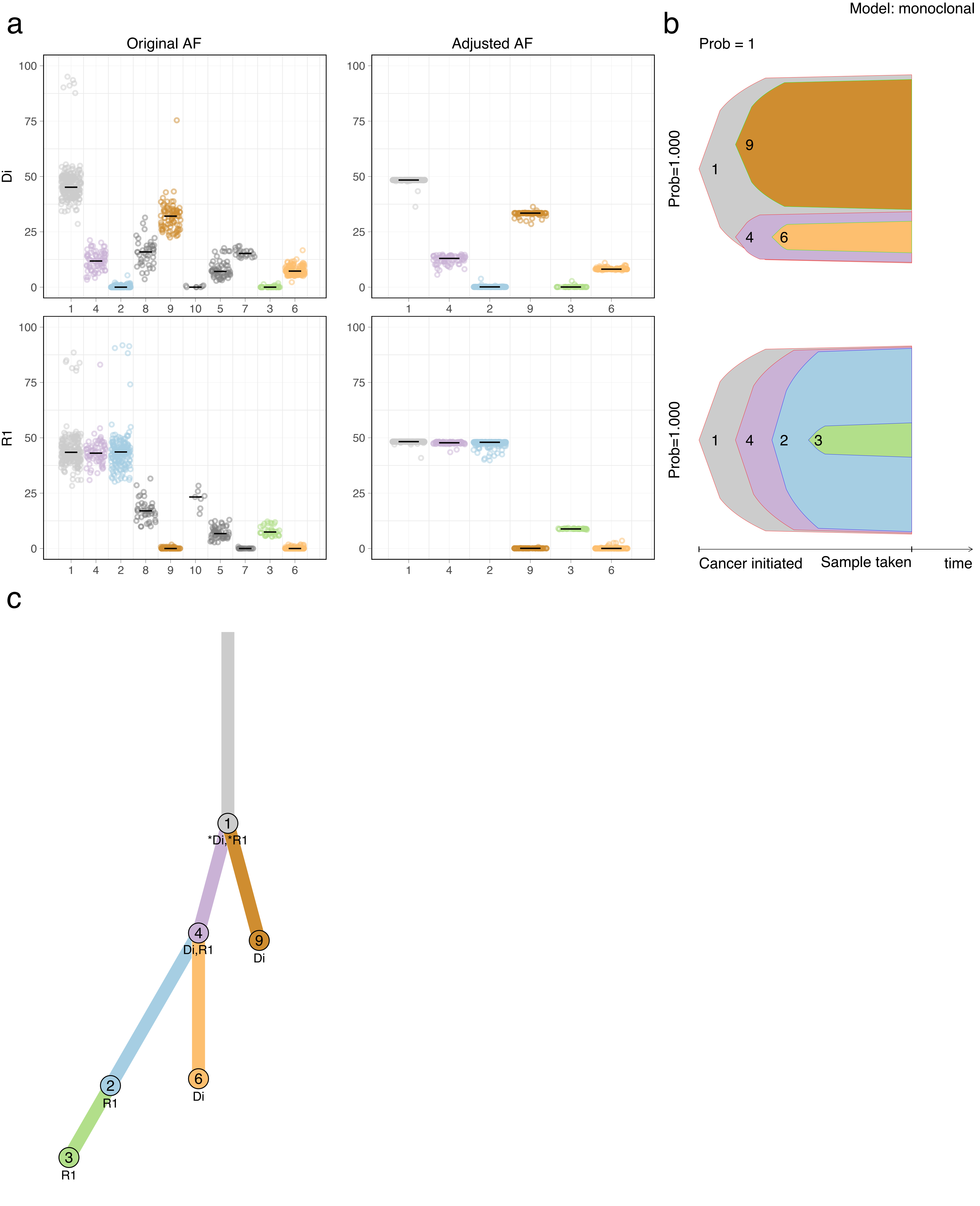

ALL_680 Rising clone

Excluding clusters: 7, 6
Adjusting cluster medians: Di 4:−2, R1 5:−2
Model: monoclonal

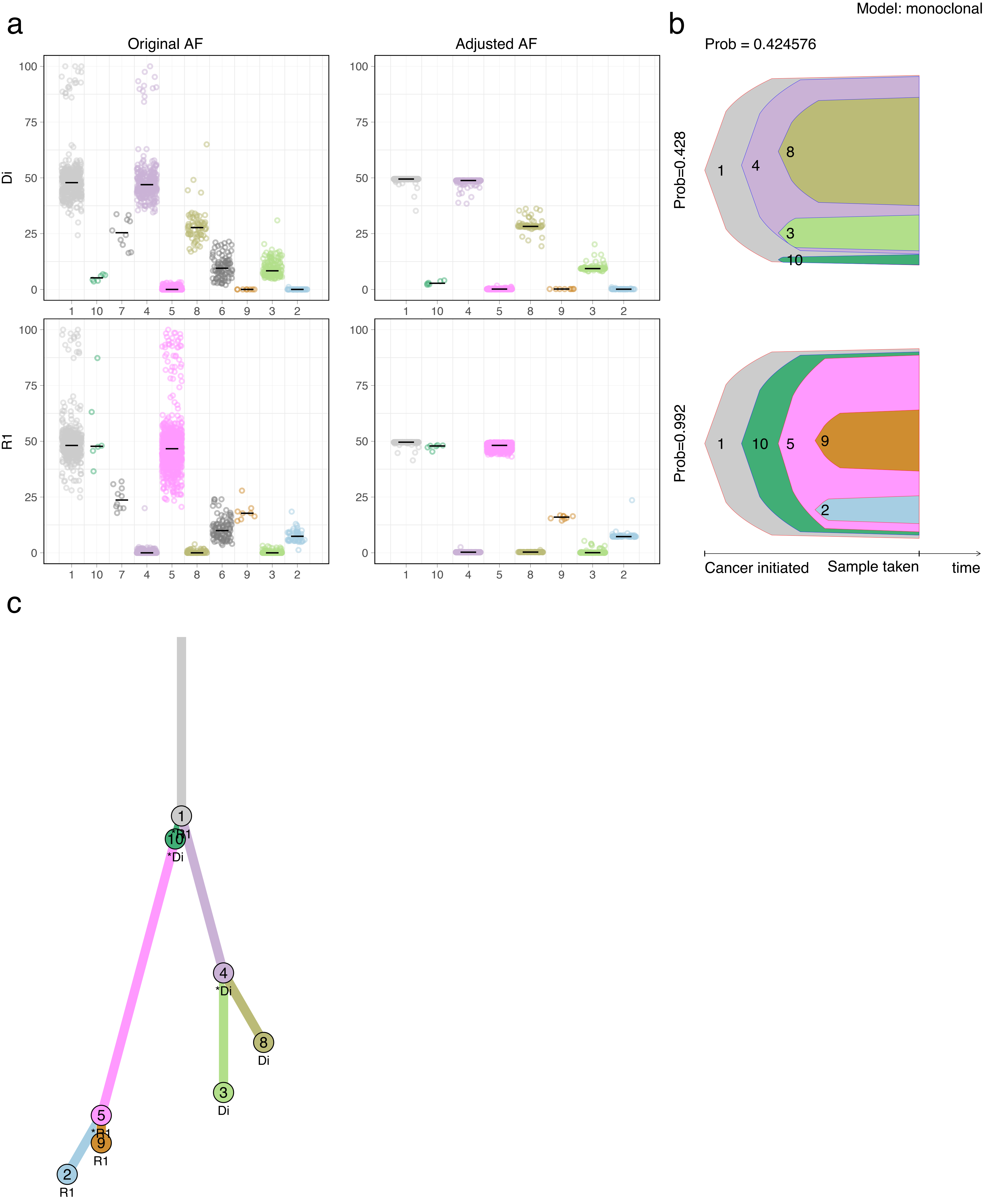

**Supplementary Fig. S5. Time from diagnosis to relapse in ALL patients in different clonal evolutionary trajectories.**

The risk group of the patients in relationship to the three clonal evolutionary trajectories is plotted. Each color represents a patient in one of four risk groups (HR=high risk; infant; IR=Intermediate risk; SR=standard risk) and each shape (circle, square, triangle) represents the immunophenotype (BCP-ALL, T-ALL and MPAL) of the patients. The Y-axis shows the time to first relapse. The horizontal dashed line is shown at 36 months from diagnosis.

# Figure S5

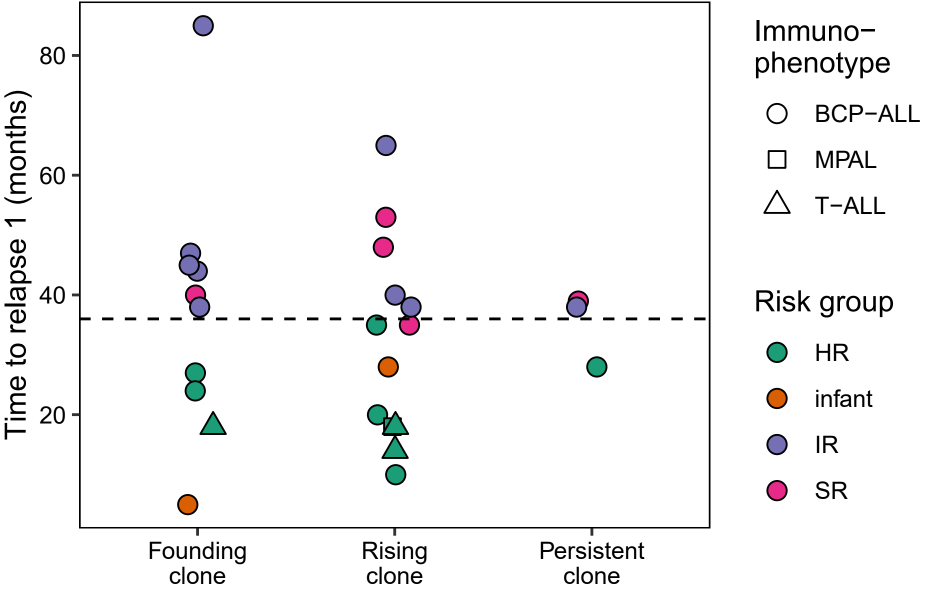

Time to relapse 1 (months)
80
60
40
20
Founding clone
Rising clone
Persistent clone
Immuno−phenotype
BCP−ALL
MPAL
T−ALL
Risk group
HR
infant
IR
SR

**Supplementary Fig. S6. Distribution of overlapping variant allele calls between triplicate sequencing libraries from each of the 67 patients with ALL**

The proportion of variant alleles called in triplicates sequencing libraries from each of the 67 ALL samples are shown on the horizontal axis and the ALL sample ID are shown on vertical axis. The alternative allele detected in all three libraries is shown in green, detected in two libraries is shown in blue, and detected in one library is shown in orange. ALL_244r shown in orange was only sequenced in one library due limitations in the amount of DNA available.

# Figure S6

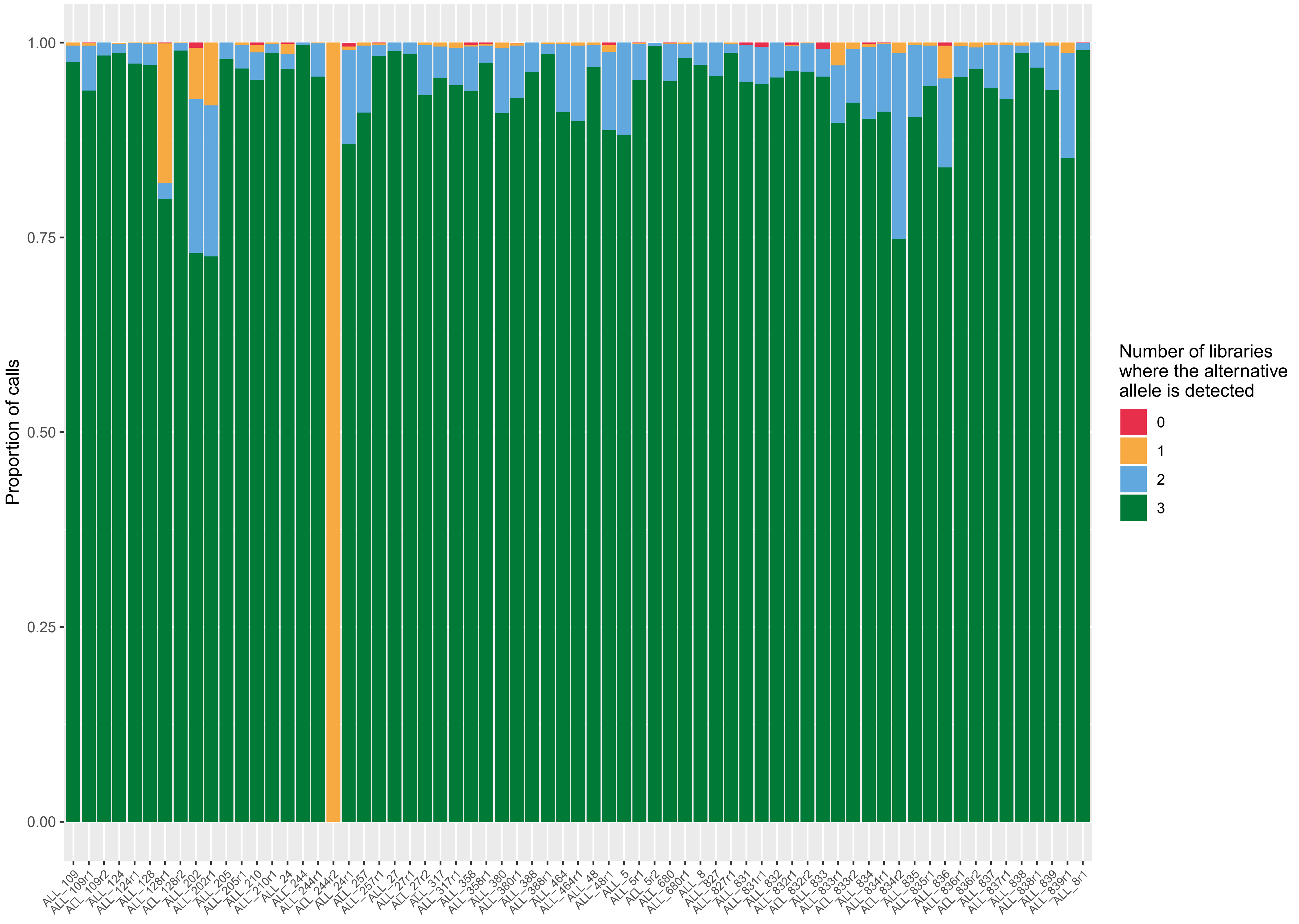
Proportion of calls
1.00
0.75
0.50
0.25
0.00
Number of libraries where the alternative allele is detected
0
1
2
3
ALL_109
ALL_109r1
ALL_109r2
ALL_124
ALL_124r1
ALL_128
ALL_128r1
ALL_128r2
ALL_202
ALL_202r1
ALL_205
ALL_205r1
ALL_210
ALL_210r1
ALL_24
ALL_244
ALL_244r1
ALL_244r2
ALL_24r1
ALL_257
ALL_257r1
ALL_27
ALL_27r1
ALL_27r2
ALL_317
ALL_317r1
ALL_358
ALL_358r1
ALL_380
ALL_380r1
ALL_388
ALL_388r1
ALL_464
ALL_464r1
ALL_48
ALL_48r1
ALL_5
ALL_5r1
ALL_5r2
ALL_680
ALL_680r1
ALL_8
ALL_827
ALL_827r1
ALL_831
ALL_831r1
ALL_832
ALL_832r1
ALL_832r2
ALL_833
ALL_833r1
ALL_833r2
ALL_834
ALL_834r1
ALL_834r2
ALL_835
ALL_835r1
ALL_836
ALL_836r1
ALL_836r2
ALL_837
ALL_837r1
ALL_838
ALL_838r1
ALL_839
ALL_839r1
ALL_8r1
